# Binding assays enable discovery of Tet(X) inhibitors that combat tetracycline destructase resistance†

**DOI:** 10.1039/d5sc00964b

**Published:** 2025-05-07

**Authors:** Matthew J. Beech, Edmond C. Toma, Helen G. Smith, Maria M. Trush, Jit H. J. Ang, Mei Y. Wong, Chung H. J. Wong, Hafiz S. Ali, Zakia Butt, Viha Goel, Fernanda Duarte, Alistair J. M. Farley, Timothy R. Walsh, Christopher J. Schofield

**Affiliations:** a Chemistry Research Laboratory, Department of Chemistry and the Ineos Oxford Institute for Antimicrobial Research, University of Oxford Oxford OX1 3TA UK christopher.schofield@chem.ox.ac.uk; b Department of Biology and the Ineos Oxford Institute for Antimicrobial Research, University of Oxford Oxford OX1 3RE UK; c Experimental Drug Development Centre (EDDC), Agency for Science, Technology and Research (A*STAR) 10, Biopolis Road Singapore 138670 Singapore

## Abstract

The Tet(X) flavin-dependent monooxygenases enable tetracycline antibiotic resistance by catalysing inactivating hydroxylation, so preventing inhibition of bacterial ribosomes. Tet(X) resistance is growing rapidly, threatening the efficacy of important last-resort tetracyclines such as tigecycline. Tet(X) inhibitors have potential to protect tetracyclines in combination therapies, but their discovery has been hampered by lack of high-throughput assays. We report the development of an efficient fluorescence polarisation Tet(X) binding assay employing a tetramethylrhodamine-glycyl-minocycline conjugate that enables inhibitor discovery. The assay was applied to tetracycline substrates and reported inhibitors, providing insight into their binding modes. Screening of a bioactive molecule library identified novel Tet(X) inhibitors, including psychoactive phenothiazine derivatives and the 5-HT_4_ agonist tegaserod, the activities of which were validated by turnover assays. Crystallographic studies of Tet(X4)-inhibitor complexes reveal two new inhibitor binding modes, importantly providing evidence for active site binding of Tet(X) inhibitors that do not share structural similarity with tetracycline substrates. In some cases, potentiation of tigecycline activity was observed in bacteria expressing Tet(X4). The combined results provide non-tetracycline scaffolds for development of potent Tet(X) inhibitors and highlight the need to evaluate the impact of non-antibiotics on antimicrobial resistance.

## Abstract 2

A fluorescence polarisation binding assay for Tet(X), an emerging tetracycline antibiotic resistance mechanism, was developed. Application of the assay resulted in the discovery of inhibitors with novel binding modes.

## Graphical abstract

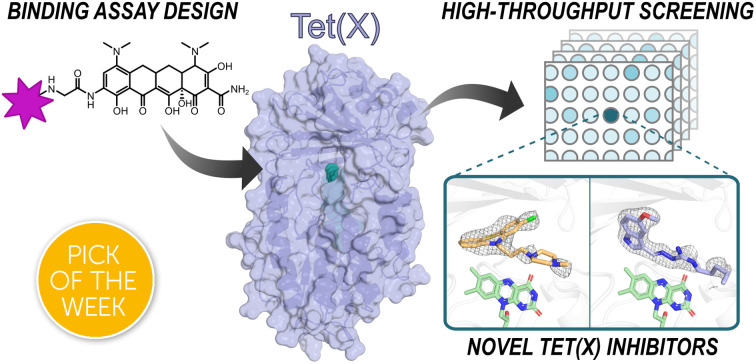

## Introduction

Tetracyclines are a medicinally vital family of polyketide antibiotics with broad-spectrum antibacterial activity; they are one of the most utilised classes of antibiotics in humans^1^ and are on the World Health Organisation's list of essential medicines.^2^ Tetracyclines are also extensively administered to livestock worldwide and used in feed as growth promoters.^3^ As a consequence of their vast clinical and agricultural use, the therapeutic effectiveness of tetracyclines is increasingly being compromised by resistance.^4^

**Fig. 1:**
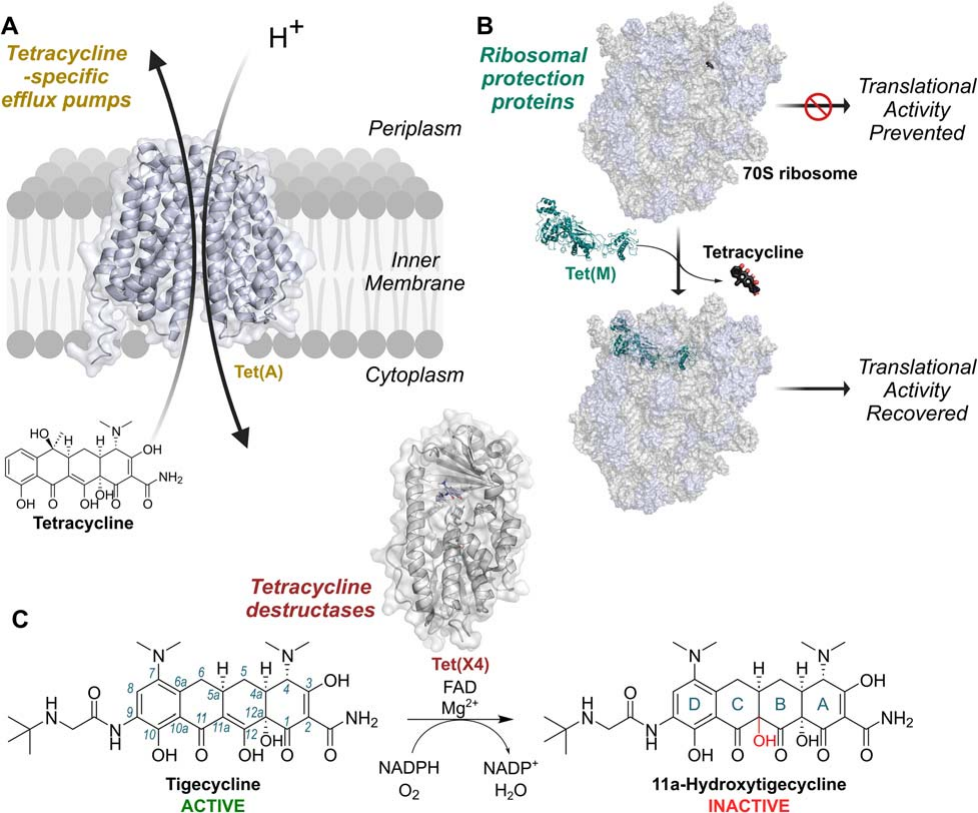
Mechanisms of resistance to tetracycline antibiotics found on mobile genetic elements. (A) Tetracycline-specific efflux pumps reduce intracellular concentrations of tetracyclines by utilising proton-motive force. (B) Ribosomal protection proteins displace tetracyclines from the ribosomal decoding centre (PDB: 3J9Y and 8CF1).^6,7^ (C) Tetracycline destructases, such as Tet(X4),^8^ are flavin-dependent monooxygenases catalysing hydroxylation of tetracyclines at the 11a-position, resulting in decreased affinity for Mg^2+^ and lower potency. Tet(X) enzymes are active against 3rd generation tetracyclines, such as tigecycline, to which the other mechanisms do not usually confer resistance. Conventional tetracycline scaffold carbon numbering (1–12) and ring nomenclature (A–D) are illustrated with tigecycline and its hydroxylation product, respectively.

The two best characterised mechanisms of resistance to tetracyclines in clinical isolates are tetracycline-specific efflux pumps, such as Tet(A) and Tet(K)^5^ (Fig. 1a), and ribosomal protection proteins, such as Tet(M) and Tet(O)^5,9^ (Fig. 1b). The emergence of these mechanisms inspired the development of clinically-used third-generation tetracyclines, that is the semisynthetic minocycline derivatives tigecycline (Tigacyl™) and omadacycline (Nuzyra™),^10,11^ as well as the fully synthetic derivative eravacycline (Xerava™).^12^ These tetracyclines possess substitutions at the C9 position of the D-ring, which extend interactions with the ribosome, thereby improving potency.^13^ Tigecycline, in particular, is a widely used last-resort antibiotic for treatment of multidrug-resistant infections.^14^ Importantly, the common efflux pump and ribosomal protection resistance mechanisms confer minimal resistance to third-generation tetracyclines.^15^

Tetracycline destructases (TDases) are an emerging family of flavin-dependent monooxygenase (FMO) enzymes providing resistance to tetracyclines by catalysing C11a hydroxylation^16^ (Fig. 1c). C11a hydroxylation is proposed to hinder chelation of the tetracycline core to Mg^2+^ ions, thereby blocking binding to ribosomes and abolishing antibiotic activity.^16^ There are two proposed classes of TDases:^17,18^ the type 2 TDases, which are predominantly found in soil-derived bacteria^17^ and are typified by the well-studied Tet(50) enzyme;^19,20^ and the type 1 TDases, encompassing the Tet(X) enzymes.^16,21^ The type 1 TDases represent the most immediate threat to the long-term efficacy of all tetracycline antibiotics. Plasmid-borne *tet(X)* genes have rapidly emerged in pathogens including *Escherichia coli*,^22,23^*Acinetobacter baumanii*,^24^*Klebsiella pneumoniae*^25^ and *Pseudomonas aeruginosa*,^26^ and co-occur with β-lactam and colistin resistance genes.^27,28^ This has resulted in the identification of *tet(X)*-harbouring resistant bacteria in hospitals across the world.^29–32^ Importantly, Tet(X) enzymes, unlike their type 2 TDase counterparts, degrade third-generation tetracyclines, including tigecycline.^17,21,33,34^

A strategy to overcome type 1 TDase resistance mechanisms is to develop specific small molecule inhibitors to be administered in combination with tetracycline antibiotics. An analogous approach has proved remarkably successful for combatting β-lactam antibiotic resistance caused by β-lactamase-catalysed degradation, with several combination therapies containing a β-lactam antibiotic and β-lactamase inhibitor currently in clinical use.^35,36^ A limited number of TDase inhibitors are reported. Anhydrotetracycline (aTC), a naturally-occurring biosynthetic intermediate and decomposition product of tetracycline, and several semi-synthetic anhydrotetracycline derivatives thereof, have shown activity in biochemical inhibition assays^17,33,37^ and are able to rescue the activity of tetracycline antibiotics against *E. coli* expressing type 1 TDases.^17,33,34^ Other reported type 1 TDase inhibitors include the naphthoquinone-based natural products plumbagin^38^ and 2-methoxy-1,4-naphthoquinone (2-MNQ),^39^ the anti-retroviral drug azidothymidine (AZT),^40^ and various bismuth salts.^41^ The exact mechanisms of inhibition by these molecules have not been identified, although all have been proposed to bind to the Tet(X) active site based on docking studies.^38–41^

Presently, the only biochemical assays that have been implemented for characterising inhibitors of TDases rely on optical absorbance measurements.^17,33,34,37,38,40^ Due to the chromophores of both the conjugated phenyl-enol moiety of the tetracyclines and the cofactor NADPH, the TDase-catalysed reaction can be monitored by measuring the decrease in optical density of either absorbance maxima; 380–400 nm for the tetracycline substrate or 340 nm for NADPH. However, assays with an absorbance-based readout are prone to interference from inhibitors that absorb light of a similar wavelength and are inherently difficult to miniaturise into sensitive, high-throughput assays.

Here we report on the development of a fluorescence polarisation (FP) binding assay and its application for the discovery of new type 1 TDase inhibitors. A minocycline-fluorophore conjugate probe was designed based on analysis of a crystal structure of Tet(X4) in complex with tigecycline. FP measurements show that the probe binds type 1 TDases with low nanomolar affinity and that substrates and inhibitors of TDases can displace the probe, enabling identification of active site-binding compounds in a robust, high-throughput manner. Use of the assay to screen an approved drug library identified six hit compounds; inhibition of Tet(X4) by them was validated through further biochemical and crystallographic studies. The combined studies reveal the binding modes of two new classes of type 1 TDase inhibitors, providing scaffolds for the development of potent compounds that can restore tetracycline activity against resistant bacterial strains and highlighting the need to explore the effects of non-antibiotic treatments on AMR.

## Results and discussion

### Design and synthesis of fluorescence polarisation probes

**Fig. 2:**
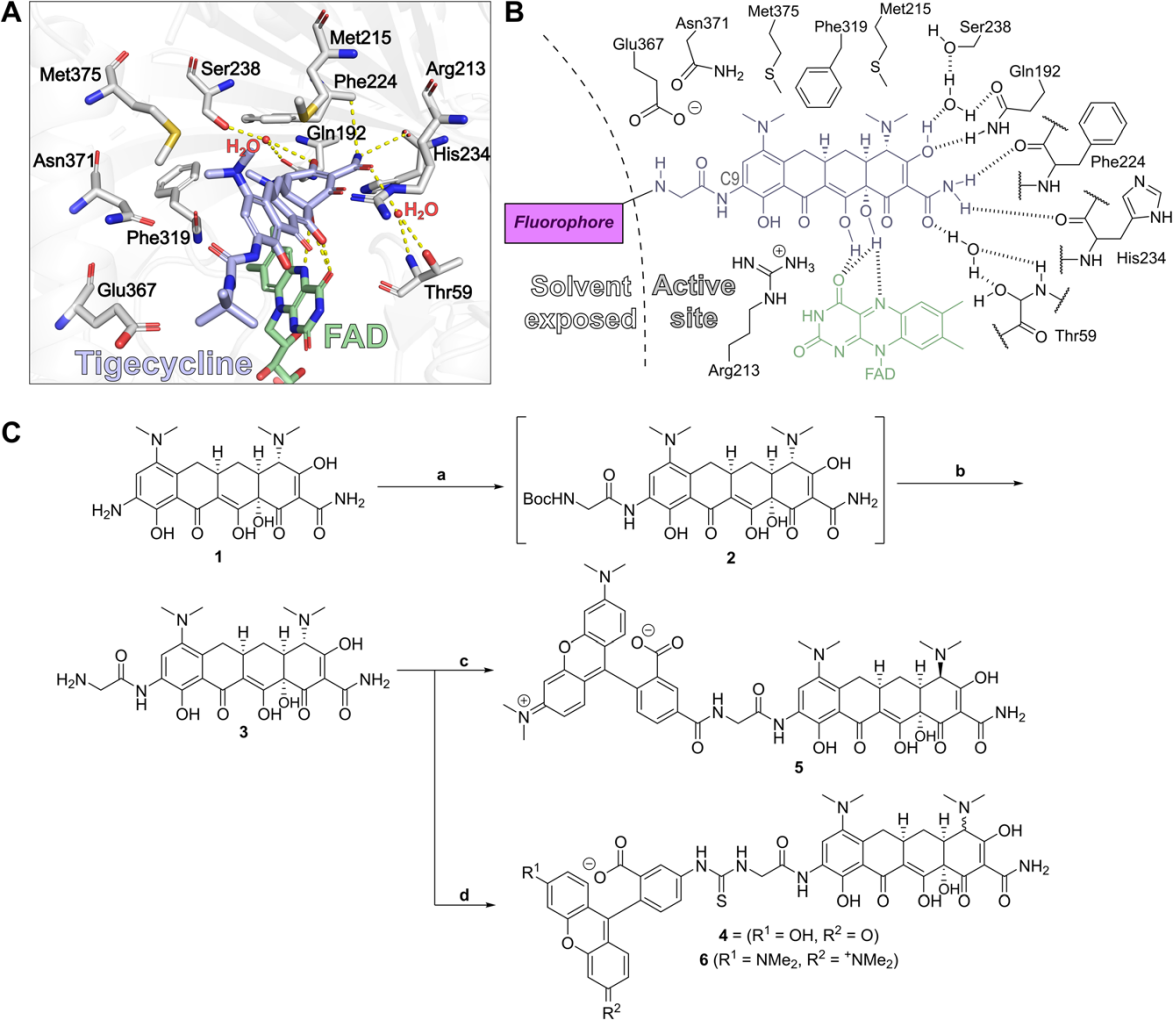
The binding mode of tigecycline in complex with Tet(X4) informed the design of fluorophore-minocycline conjugates as fluorescence polarisation probes. (A) View of tigecycline in the active site of Tet(X4)^8^ (PDB: 7EPW), highlighting key residues predicted to interact with the substrate. C9 of the tetracycline scaffold is directed towards bulk solvent, allowing attachment of a fluorophore conjugates for FP experiments. (B) Schematic of the Tet(X4) active site architecture illustrating design considerations for development of a fluorescent probe. (C) Synthesis of fluorescent probes 4–6. Reagents: (a) DIPEA, HATU, Boc-Gly; (b) 4N HCl, dioxane, 95% over 2 steps; (c) 5-TAMRA, HATU, DIPEA, DMF, 44%; (d) FITC, NEt_3_, DMF, 74% or 5-TRITC, DIPEA, DMF, 64%.

Crystal structures of tigecycline complexed with Tet(X2) (PDB ID: 4A6N)^42^ and Tet(X4) (PDB ID:7EPW)^8^ served as a basis for the design of potential fluorescent probes (4–6) for type 1 TDases. Examination of the structures showed that the 9-*tert*-butylglycylamido group of tigecycline projects out of the active site towards bulk solvent (Fig. 2a and b), and therefore was a plausible vector for attachment of a fluorophore. 9-Glycylamido-minocycline (3) was thus synthesised from 9-amino minocycline (1) following a reported route^43^ and coupled with fluorescein isothiocyanate (FITC) to form the desired fluorescein-5-thiourea probe 4 (Fig. 2c); interestingly, the coupling conditions yielded predominantly the (4*R*)-epimer. However, whilst initial FP experiments suggested 4 may bind to recombinant Tet(X4), substantial background fluorescence was observed in controls lacking the fluorescent molecule (ESI Fig. S1†); experiments showed this is likely due to the intrinsic fluorescence of the flavin cofactor overlapping with that of fluorescein (ESI Fig. S2†).

We therefore investigated red-shifted tetramethylrhodamine dyes as fluorophores because they possess relatively long excitation and emission wavelengths,^44^ decreasing potential for interference. 5-Carboxytetramethylrhodamine (5-TAMRA) and tetramethylrhodamine-5-isothiocyanate (5-TRITC) dyes were reacted with 9-glycylamido minocycline to give fluorescent conjugates 5 and 6, respectively (Fig. 2c). Probe 5 was synthesised using standard amide coupling conditions with hexafluorophosphate azabenzatriazole tetramethyl uronium (HATU) as the coupling agent and *N*,*N*-diisopropylethylamine (DIPEA) as the base. As with 4, following purification, only the C4-(*R*) epimer was observed. Probe 6 was synthesised utilising 5-TRITC and DIPEA and, after purification, the product was obtained as an ∼1 : 1 mixture of (4*S*) and (4*R*) epimers.

### Binding of fluorescent probes to tetracycline destructases

The equilibrium binding dissociation constants, *K*_d_, for probes 5 and 6 with the Tet(X) isoforms Tet(X2), Tet(X3), Tet(X4), Tet(X5) and Tet(X7), as well as homologous Tet(50), were determined by titrating increasing concentrations of recombinantly overproduced and highly purified enzymes (>90% purity by SDS-PAGE analysis, ESI Fig. S3†) with a constant concentration of fluorescent probe in the presence of excess MgCl_2_ and fixed concentration of FAD (Table 1, ESI Fig. S4†). NADPH was not included to prevent turnover of the probe to the hydroxylated product, which would likely have a lower binding affinity than the initial probes. Screens were conducted in pH 7.0 buffer with addition of 0.01% Triton-X100 detergent in non-binding plates to minimise adsorption to the plate wells.^45^

**Table 1:** Equilibrium binding constants (*K*_d_) measured for FP probes 5 and 6 with a selection of purified TDases show a low nanomolar binding affinity for Tet(X) variants

Enzyme	*K*_d_ a/nM	
	Probe 5	Probe 6
Tet(X2)	66.9 ± 10.3	22.4 ± 4.0
Tet(X3)	24.3 ± 2.2	7.7 ± 0.3
Tet(X4)	46.5 ± 4.1	17.3 ± 1.9
Tet(X5)	29.9 ± 2.7	10.6 ± 1.3
Tet(X7)	126 ± 15	40.5 ± 5.1
Tet(50)	>5000	>5000

a Using 100 mM Tris (pH 7.0) and 0.01% Triton X-100 with 25 nM 5 or 6, 5 mM MgCl_2_ and 1 μM FAD. Fluorescence polarisation was measured with *λ*_ex_ = 540 ± 20 nm, *λ*_em_ = 590 ± 20 nm.

With both 5 and 6, the binding affinities were strongest for Tet(X3) and weakest for Tet(X7). The difference however was relatively small, with a ∼5-fold difference in *K*_d_ between Tet(X3) and Tet(X7) for both probes, consistent with the high Tet(X3) and Tet(X7) sequence identity and conserved active site residues and topology of Tet(X) enzymes^8,17,34,46^ (ESI Fig. S5–S7†). Notably, probe 6 appeared to bind to all Tet(X) enzymes tested with greater affinity than 5, and was therefore chosen for further experiments. We focused on Tet(X4) as a model type 1 TDase because it has a particularly high prevalence,^47^ has been well-characterised biochemically and structurally,^8^ and can be produced in high yields (∼60 mg purified protein per litre of culture).

In contrast to the results with all tested type 1 TDases, 5 and 6 displayed no detectable binding to type 2 TDase Tet(50) up to a concentration of 5 μM of enzyme (ESI Fig. S8†). Lack of Tet(50) binding is likely due to the large C9 substituents of 5 and 6 sterically clashing with the C-terminal helix found in Tet(50) and other type 2 TDases, which is not present in the Tet(X) enzymes (ESI Fig. S9†). This proposal agrees with the observed lack of turnover of tigecycline by Tet(50),^19,34^ which also possesses a C9-substituted D-ring.

To investigate whether fluorescent probe 6 binds to the active site of the Tet(X) enzymes, we carried out docking of the probe with Tet(X4)^8^ (ESI Fig. S10†). Inspection of the docked complex suggested that probe 6 binds similarly to tigecycline, with the reactive minocycline core binding proximal to the FAD cofactor. We thus considered that probe 6 may be hydroxylated by Tet(X4) when NADPH was added to initiate turnover. We monitored turnover of probe 6 using an absorbance-readout assay with Tet(X4) adapted from previous studies.^33^ A decrease in absorbance at 400 nm was observed, consistent with hydroxylation, but was not seen in a control reaction lacking Tet(X4) (ESI Fig. S11†). This observation was corroborated by LC-MS analysis of the reaction (ESI Fig. S12†). Appearance of an apparently mono-hydroxylated species with mass 989.348 Da (calculated mass: 989.3504 Da) was observed, +16 Da relative to the starting material (measured mass: 973.355 Da; calculated mass: 973.3555 Da). These experiments demonstrate 6 can be turned over by Tet(X4) and therefore likely binds to the active site in a similar manner to other tetracyclines. Subsequent competitive binding studies showed that tetracycline substrates can displace 6 (see below), providing further evidence for this proposal.

### Assay optimisation

**Fig. 3:**
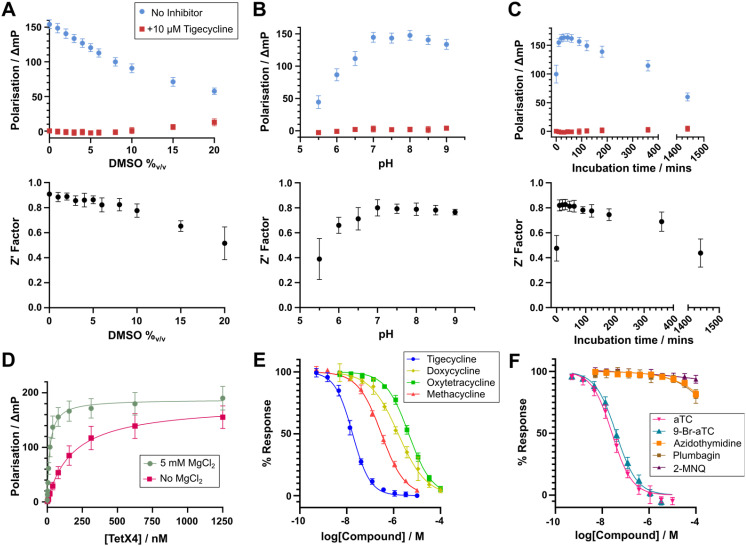
Optimisation of competitive binding experiments with probe 6 enabled high-throughput and quantitative measurement of substrate and inhibitor binding. All experiments were conducted in buffer containing 100 mM Tris (pH 7.0), 0.01%_v/v_ Triton X-100 with 25 nM 6, 40 nM Tet(X4), 5 mM MgCl_2_ and 1 μM FAD. See materials and methods for details. (A) DMSO tolerance of the fluorescence polarisation assay (*n* = 48). (B) pH tolerance of the fluorescence polarisation assay (*n* = 66). (C) Dependency of the fluorescence polarisation assay on incubation time (*n* = 396). For (A)–(C), the top graph shows the average and standard deviation for positive and negative controls for each condition, the bottom graph shows the *Z*′ factor calculated from three independent replicates for each condition. (D) Apparent *K*_d_ curves for the binding of Tet(X4) with probe 6 in the presence and absence of 5 mM MgCl_2_ demonstrating a clear dependence of binding on presence of Mg^2+^. The apparent *K*_d_ values measured were 18.1 ± 2.4 nM and 188 ± 33 nM, with and without MgCl_2_, respectively. (E) Dose–response curves for competitive binding experiments with select tetracycline derivatives (*n* = 12). (F) Dose–response curves for competitive binding experiments with selected Tet(X) inhibitors described in previous studies (*n* = 12).^33,38,39^ aTC = anhydrotetracycline, 9-Br-aTC = 9-bromoanhydrotetracycline, 2-MNQ = 2-methoxy-1,4-naphthoquinone. In all cases, error bars represent standard deviations across 3 independent experiments.

We optimised the conditions to give a robust FP binding assay for measuring Tet(X4) binding in a 384-well format. The assay showed good tolerance for the inclusion of DMSO (Fig. 3a). The difference in signal between the positive and negative control remained high (>100 mP) up to a concentration of 5% DMSO; the *Z*′ factor for the assay remained >0.8 over this same range with minimal variability in triplicate independent repeats, indicating robustness over the typical DMSO concentrations used for compound screening.^48^ Over a pH range of 5.5–9, values of >7.0 were preferred, with a substantial decrease in the positive control response and *Z*′ factor observed at acidic pH values (Fig. 3b). The incubation time with probe 6 was varied to determine the optimal time to incubate the components prior to measuring the FP response (Fig. 3c). The positive control response and *Z*′ factor rapidly increased to a plateau at the 10 min timepoint and remained relatively stable over the course of 6 hours before significantly decreasing. The combination of high *Z*′ factors, 384-well format and low enzyme and probe requirements make this assay amenable to high-throughput screening approaches.

MgCl_2_ is required for TDase activity,^16,21,33^ though to our knowledge, the mechanism for the Mg^2+^ dependence of TDases is unknown. With a fixed concentration of probe 6 and Tet(X4), titration of MgCl_2_ increased the polarisation response in a dose-dependent manner (ESI Fig. S13†). Furthermore, measuring the *K*_d_ of Tet(X4) for probe 6 with and without the addition of 5 mM MgCl_2_ manifested a 10-fold increase in binding affinity when Mg^2+^ was present (18.1 ± 2.5 nM *versus* 188 ± 33 nM, Fig. 3d). These results suggest that one or more Mg^2+^ ions facilitates binding of tetracyclines to the Tet(X4) active site and, possibly, that TDases act preferentially upon a tetracycline-Mg^2+^ complex, in an analogous manner to the isocitrate-Mg^2+^ complex binding to isocitrate dehydrogenase.^49^ However, as no Mg^2+^ ions have been assigned in crystal structures of TDases in complex with tetracyclines, further biochemical and structural biology experiments are required to understand the exact function of Mg^2+^ in TDase catalysis, which may be important to inform inhibitor design. By contrast, varying the added FAD concentration had a minimal effect on the polarisation response (ESI Fig. S14†).

### Competitive binding assays with Tet(X4)

To evaluate the utility of probe 6 for inhibitor discovery, we first tested the ability of known TDase substrates and inhibitors to displace it, which should be accompanied by a dose-dependent reduction in the FP response enabling calculation of apparent half-maximal inhibitory concentration (IC_50_^app^) values. We observed clear displacement of the probe with a selection of marketed tetracyclines in dose–response competitive binding experiments (Fig. 3e). Across nine assay plates measuring IC_50_^app^ values in triplicate for these compounds, we measured average *Z*′ factors of 0.85 ± 0.03, well above the accepted cut-off limit of 0.4,^48^ indicating a robust assay amenable to high-throughput screening approaches. Most tetracyclines gave IC_50_^app^ values in the low nanomolar range (Table 2), indicating a high affinity for Tet(X4). The lowest IC_50_^app^ values were observed for tigecycline and eravacycline (18.6 ± 4.9 and 13.7 ± 1.6 nM, respectively), which both possess a C9-glycylamido group. A crystal structure of tigecycline complexed with Tet(X2) (PDB: 4A6N) reveals the C9 amine may be positioned to interact with the conserved active site residue Glu367,^42^ which may account for the higher affinity of these substrates.

**Table 2:** Half maximal inhibitory concentration (IC_50_) values measured for known inhibitors and substrates of Tet(X) enzymes in fluorescence polarisation and orthogonal activity assays

	Compound^a^	IC_50_ values, Tet(X4)^b^		
		FP assay^c^/nM	Absorbance assay^d^/μM	UPLC assay^e^/μM
Tetracycline substrates	Tigecycline	18.6 ± 4.9^f^	—	—
	Minocycline	51.5 ± 3.6	—	—
	Tetracycline	69.6 ± 8.8	—	—
	Chlortetracycline	39.0 ± 9.6	—	—
	Oxytetracycline	4430 ± 260	—	—
	Doxycycline	1580 ± 150	—	—
	Demeclocycline	29.0 ± 4.6	—	—
	Methacycline	311 ± 36	—	—
	Omadacycline	24.3 ± 4.0	—	—
	Eravacycline	13.7 ± 1.6	—	—
	Sarecycline	31.5 ± 10.0	—	—
	Sancycline	47.0 ± 7.1	—	—
Tet(X) inhibitors	Anhydrotetracycline	26.8 ± 5.3	7.6 ± 2.0	2.6 ± 0.3
	9-Bromo-anhydrotetracycline	40.4 ± 10.5	4.5 ± 0.6	2.7 ± 0.5
	Plumbagin	>100 000	49.1 ± 4.9	37.2 ± 8.2
	2-Methoxy-1,4-naphthoquinone	>100 000	421 ± 50	>100
	Azidothymidine	>100 000	>1000	>100

a Structures for compounds are given in ESI Fig. S15 and S16.

b Mean ± standard deviations for three independent replicates.

c Using 25 nM 6, 40 nM Tet(X4), 5 mM MgCl_2_ and 1 μM FAD in 100 mM Tris (pH 7.0) with 0.01%_v/v_ Triton X-100.

d Using 25 μM tigecycline, 50 nM Tet(X4), 250 μM NADPH, 5 mM MgCl_2_ and 1 μM FAD in 100 mM TAPS (pH 8.5).^33,37^

e Using 20 μM tigecycline, 50 nM Tet(X4), 100 μM NADPH, 5 mM MgCl_2_ and 1 μM FAD in 100 mM TAPS (pH 8.5).

f Mean ± standard deviations for nine independent replicates.

**Fig. 4:**
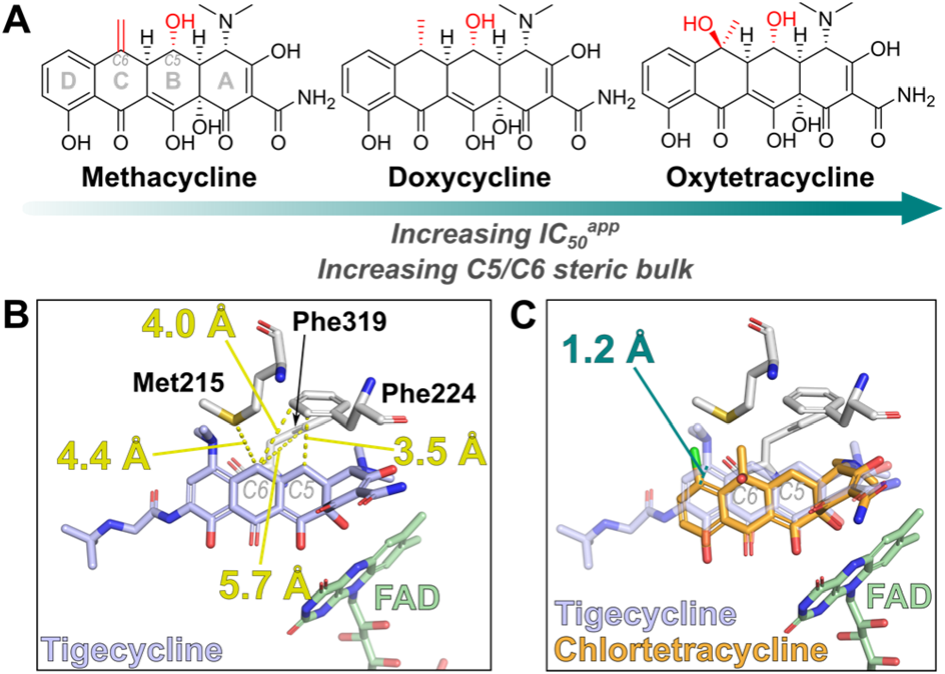
Increasing C5 and C6 steric bulk of tetracyclines results in weaker affinity, offering a strategy for development of tetracyclines that evade Tet(X) resistance. (A) Structure of C5- and C6-substiuted tetracyclines tested in this study. (B) Distance measurements between C5/C6 of tigecycline and nearby active site residues (PDB: 7EPW).^8^ (C) Displacement of chlortetracycline, a C6-substituted tetracycline, in complex with Tet(X2) relative to tigecycline (PDB: 2Y6R).^46^ This displacement is likely necessary to avoid steric clashes of the C6-substitutions with active site residues. Superimposition of structures was performed in PyMOL v2.5.0 (Schrödinger, LLC).

Of interest within the tested tetracycline set, methacycline, doxycycline and oxytetracycline displayed substantially weaker affinity for Tet(X4) than the other tetracyclines, with IC_50_^app^ values of 310 ± 36 nM, 1.58 ± 0.15 μM and 4.43 ± 0.26 μM, respectively. These tetracyclines share a (5*R*)-hydroxyl group on their B-ring, as well as substitution at the C6 position of the C-ring (Fig. 4a). In the structures of type 1 TDases complexed with a tetracycline antibiotic, C5 is proximal to Phe224 (Fig. 4b).^8^ We propose that addition of an (*R*)-hydroxyl group at C5 may cause a steric clash with Phe224, reducing the affinity of these tetracyclines for Tet(X4). The IC_50_^app^ also increases with increasing steric bulk at the C6 position, with C6-di-substituted oxytetracycline having the lowest affinity for Tet(X4). The only type 1 TDase complex structure with a C6-substituted tetracycline is that of Tet(X2) complexed with chlortetracycline (PDB: 2Y6R),^46^ the binding mode of which is shifted by 1.2 Å relative to other tetracycline substrate complexes (Fig. 4c), likely to accommodate the substitution. These observations suggest that a potential strategy for developing a tetracycline that evades type 1 TDase-mediated resistance is to explore the structure–activity relationships of substituents at the tetracycline C5 and C6 positions, which could hinder binding to the active site. There is precedent for tetracyclines with substituents at these positions^50–52^ with some manifesting antibacterial activity,^50^ suggesting retention of potency whilst evading TDase-mediated resistance may be possible.

**Fig. 5:**
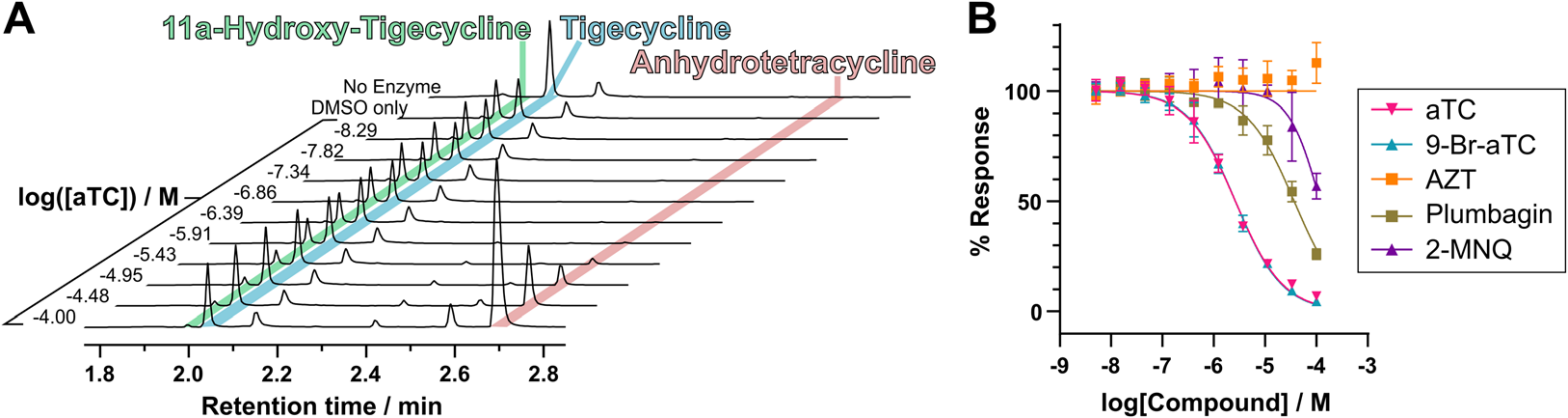
A novel chromatography-based activity assay allows measurement of IC_50_ values with reduced interference from UV-active compounds. (A) UPLC chromatograms demonstrating dose-dependent inhibition of Tet(X4) using known inhibitor anhydrotetracycline as an example. Chromatograms show the absorbance at 254 nm in arbitrary units. (B) Dose–response curves for inhibition of Tet(X4) activity, monitored by UPLC peak integrals to calculate turnover, with previously reported inhibitors.^33,38,39^ Conditions were: 20 μM tigecycline, 50 nM Tet(X4), 10 μM FAD, 5 mM MgCl_2_ and 100 μM NADPH in 100 mM TAPS buffer (pH 8.5) with a final concentration of 1%_v/v_ DMSO.

We then tested the binding of reported type 1 TDase inhibitors to Tet(X4) (Fig. 3f, ESI Fig. S17†).^33,38,39^ We observed clear dose-dependent displacement of probe 6 with aTC and 9-Br-aTC. These compounds gave similar apparent IC_50_^app^ values, in accord with trends in IC_50_ measurements performed by Markley *et al.*^33^ With the other reported Tet(X) inhibitors plumbagin, 2-MNQ and AZT, minimal competitive binding was observed up to a maximum concentration of 100 μM. To validate these results, we performed orthogonal activity assays of Tet(X4) with tigecycline by monitoring changes in absorbance at 400 nm utilising a protocol adapted from that of Markley *et al.*,^33^ miniaturised to a 384-well format (Table 2, ESI Fig. S18†). We also developed a complementary endpoint activity assay where the turnover of tigecycline by Tet(X4) in a 96-well plate was quenched by addition of 1%_v/v_ formic acid and analysed chromatographically. The peak integrals of tigecycline and its hydroxylated degradation product at 254 nm following UPLC analysis were measured to determine turnover (Fig. 5a). As this method separates components by chromatography, it enables IC_50_ measurements independent of potential interference from UV-active inhibitors (Fig. 5b). No inhibition was seen up to 1 mM for AZT in the absorbance assay or up to 100 μM in the UPLC assay, suggesting that AZT is not a potent Tet(X4) inhibitor (Table 2). The apparent synergy of AZT with tetracyclines against whole cells expressing Tet(X4)^40^ is therefore interesting and may derive from the proposed ability of AZT to interfere with DNA synthesis in bacteria,^40,53^ or other unidentified mechanisms. Plumbagin and 2-MNQ displayed moderate and weak inhibition, respectively, in both activity assays (Table 2). That the naphthoquinone derivatives inhibit Tet(X4) but do not readily outcompete probe 6 suggests an inhibition mechanism that does not involve binding to the Tet(X4) tetracycline-binding pocket, such as binding to the proposed NADPH binding site. Plumbagin was observed to bind to isolated Tet(X4) through bio-layer interferometry measurements,^38^ supporting this hypothesis. Note, however, that plumbagin and 2-MNQ are naphthoquinones, which are well-characterised redox-active pan-assay interference (PAINS) moieties.^54^

### High-throughput screening and hit validation

**Fig. 6:**
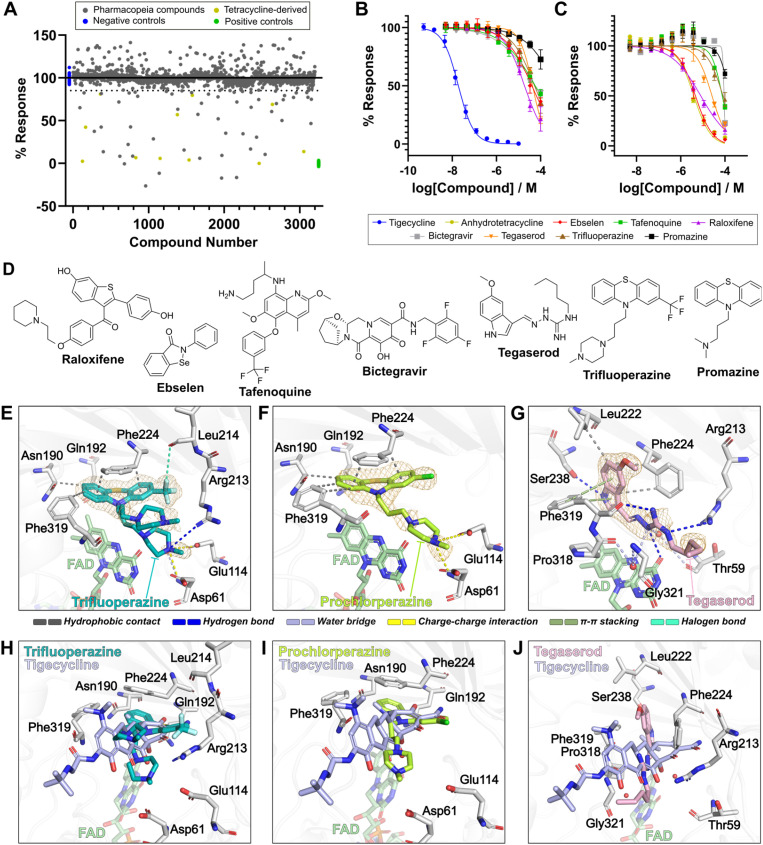
High-throughput screening identifies known pharmacologically-active molecules as Tet(X4) inhibitors with novel active site binding modes. (A) Results of screening of pharmacopeia reference drugs. Binding was measured by monitoring the FP response of compounds at a fixed concentration (10 μM), normalised to positive (no inhibitor) and negative controls (10 μM tigecycline). Conditions were 25 nM 6, 80 nM Tet(X4), 5 mM MgCl_2_ and 1 μM FAD in 100 mM Tris (pH 7.0) with 0.01%_v/v_ Triton X-100. (B) Dose–response validation of selected hit compounds using the FP binding assay. Conditions: 25 nM 6, 40 nM Tet(X4), 5 mM MgCl_2_ and 1 μM FAD in 100 mM Tris (pH 7.0) with 0.01%_v/v_ Triton X-100. (C) Dose–response validation of selected hit compounds using a UPLC-readout activity assay. Conditions: 20 μM tigecycline, 50 nM Tet(X4), 100 μM NADPH, 5 mM MgCl_2_ and 1 μM FAD in 100 mM TAPS (pH 8.5). (D) Structures of validated hit compounds. (E) Active site view of two alternate conformations of trifluoperazine in complex with Tet(X4) (PDB: 9HKE). (F) Active site view of prochlorperazine in complex with Tet(X4) (PDB: 9HJV). (G) Active site view of tegaserod in complex with Tet(X4) (PDB: 9HJW). Orange mesh in (E)–(G) represent *mF*_obs_–*DF*_model_ polder OMIT maps^55^ contoured to 3*σ* (trifluoperazine) or 4*σ* (prochlorperazine and tegaserod) and carved around ligands at 1.8 Å. Enzyme-ligand interactions were predicted using protein-ligand interaction profiler.^56^ (H) Active site view of the trifluoperazine-Tet(X4) complex compared to the tigecycline-Tet(X4) binding mode (PDB: 7EPW).^8^ (I) Active site view of the prochlorperazine-Tet(X4) complex compared to the tigecycline-Tet(X4) binding mode. (J) Active site view of the tegaserod-Tet(X4) complex compared to the tigecycline-Tet(X4) binding mode. Visualisation and superimposition of structures was performed using PyMOL v2.5.0 (Schrödinger, LLC).

To evaluate the utility of our FP assay for discovery of novel Tet(X) inhibitors, we screened 3200 Pharmacopeia reference drugs across 10 assay plates at 10 μM final concentration (Fig. 6a). We selected a 15% inhibition cut-off so as not to lose potential weak-binding compounds which could serve as starting points for further optimisation. We prioritised hits by removing those with known fluorescence or long-wavelength absorbance, which interfered with the fluorescence readout. We discounted tetracyclines that had already been tested and compounds that were not commercially accessible. Binding of the resultant 21 compounds was investigated by dose–response experiments in our FP readout assay (Fig. 6b, ESI Table S1†). Of these 21 compounds, 11 demonstrated evidence for competitive binding (ESI Fig. S20†) and were subjected to the orthogonal UPLC-based assay (ESI Fig. S21†) to determine whether they inhibited activity of Tet(X4). Dose-dependent inhibition of Tet(X4) activity was observed for 8 compounds (Fig. 6c), with 6 having a measured IC_50_ < 100 μM. These compounds included: raloxifene, a hormone replacement therapy^57^ (IC_50_^app^ = 19.8 ± 5.4 μM, IC_50_ = 8.3 ± 2.1 μM); ebselen, a thiol-reactive organoselenium compound with several bioactivities^56^ (IC_50_^app^ = 40.9 ± 11.4 μM, IC_50_ = 4.7 ± 1.5 μM); tafenoquine, an antimalarial^58^ (IC_50_^app^ = 94.9 ± 64.8 μM, IC_50_ = 76.0 ± 2.4 μM); bictegravir, an anti-viral integrase inhibitor^59^ (IC_50_^app^ = 54.8 ± 10.6 μM, IC_50_ = 76.0 ± 14.0 μM); tegaserod, a 5-hydroxytryptamine receptor 4 (5-HT_4_) agonist^60^ (IC_50_^app^ = 43.2 ± 2.2 μM, IC_50_ = 25.9 ± 2.4 μM); and trifluoperazine, a phenothiazine drug with anti-psychotic activity^61^ (IC_50_^app^ = 55.6 ± 14.2 μM, IC_50_ = 83.8 ± 21.4 μM) (Fig. 5d).

### Phenothiazine derivatives as inhibitors of Tet(X4)

Trifluoperazine possessed moderate activity against Tet(X4), with apparent IC_50_ values of 55.6 ± 14.2 μM and 83.8 ± 21.4 μM in FP- and UPLC-based assays, respectively. Another phenothiazine drug, promazine, also displayed a weak dose-dependent response against Tet(X4) (ESI Fig. S20 and S21†), although the IC_50_ values were above the 100 μM highest concentration tested. Based on this evidence, we evaluated other phenothiazine drugs for Tet(X4) inhibition. Several phenothiazine derivatives were observed to elicit dose-dependent responses in binding and activity assays (ESI Fig. S22 and S23, ESI Table S2†). Prochlorperazine displayed improved activity compared to trifluoperazine, differing by replacement of the 2-trifluoromethyl-substituent with a chlorine, with an IC_50_^app^ of 39.9 ± 7.0 and IC_50_ 49.5 ± 24.9 μM in the FP- and UPLC-based assays, respectively. Perazines, with a 3-(4-methylpiperazin-1-yl)propyl group attached to their 10-phenothiazinyl amine, bound with greater affinity than their promazine counterparts, which possess a *N*,*N*-dimethylpropan-1-amine group at the same position. These structure–activity relationships suggest that phenothiazines are promising scaffolds for further development of Tet(X) inhibitors.

### Crystallographic studies reveal novel Tet(X) inhibitor binding modes

To investigate interactions between the validated inhibitor compounds and Tet(X4), we carried out co-crystallisation with a Tet(X4) construct with the N-terminal tag cleaved. Recombinant Tet(X4) was crystallised in the presence of excess FAD, MgCl_2_ and >3 molar equivalents of the compounds. Structures were obtained for Tet(X4) complexes with trifluoperazine (Fig. 6e, PBD: 9HKE, 1.9 Å resolution), prochlorperazine (Fig. 6f, PBD: 9HJV, 2.2 Å resolution) and tegaserod (Fig. 6g, PBD: 9HJW, 1.9 Å resolution). These complexes crystallised in the *P*6_5_22 space group with one copy per asymmetric unit, consistent with reported Tet(X4) structures.^8^ The overall fold was in good agreement with both the *apo*-Tet(X4) structure and its tigecycline-bound structure (*C*_α_ RMSD < 0.2 Å in all cases).^8^ The three compounds bind to the active site in a manner overlapping with the tigecycline binding site (Fig. 6h–j), validating their proposed mechanism of action as non-covalent competitive inhibitors. With all three compounds, the FAD cofactor appears in the so-called FAD-IN position (ESI Fig. S24†), wherein the catalytically-important isoalloxazine FAD ring is directed toward the tetracycline binding pocket, rather than the FAD-OUT conformation, where the isoalloxazine is instead directed towards the putative NADPH binding site.^62^ Amongst TDases, the FAD-OUT conformation has so far only been observed in the type 2 aTC-Tet(50) complex, whilst all substrate and inhibitor complex structures with type 1 TDases display the FAD-IN conformation. It is therefore likely that inhibitor design should be focused around active site binding with the FAD-IN conformation of type 1 TDases.

Clear electron density in the active site of Tet(X4) is observed for the tricyclic phenothiazine group in both the trifluoperazine and prochlorperazine complexes (Fig. 6e and f), including their 2-trifluoromethyl- and 2-chloro-substituents, respectively. The phenothiazine rings adopt near identical conformations (ESI Fig. S25†), suggesting a conserved binding mode. They occupy a similar space in the active site as does the A-ring of tigecycline (Fig. 6h and i) and have high shape complementarity to the Tet(X4) substrate binding pocket (ESI Fig. S26†). Analysis of the binding mode using Protein–Ligand Interaction Profiler (PLIP)^63^ reveals the phenothiazine groups make conserved hydrophobic contacts with residues Asn190, Gln192, Phe224 and Phe319 (Fig. 6e and f). A fluorine of the trifluoromethyl-group in trifluoperazine can potentially form a halogen bond with the Leu214 backbone carbonyl (Fig. 6e).

In contrast to the phenothiazine rings, the 3-(4-methylpiperazin-1-yl)propan-1-amine tails of both trifluoperazine and prochlorperazine molecules display weaker electron density (Fig. 6e and f) and higher B-factors (ESI Fig. S27†), which can likely be attributed to their intrinsic conformational lability. Trifluoperazine potentially binds in two conformations, with one appearing similar to the observed binding mode of prochlorperazine (ESI Fig. S25†). In the prochlorperazine complex and equivalent conformation with trifluoperazine, the protonated quaternary amine of the 4-methyl-piperazine group is proximal to Asp61 and Glu114 near the active site entrance, allowing for formation of a charge–charge interaction. In the case of trifluoperazine, it is predicted that this amine also forms a water bridge with the backbone amide of Asp61 and a hydrogen bond with Arg213.

In the tegaserod-Tet(X4) complex structure, clear electron density was observed for the 5-methoxyindole and guanidine moieties (Fig. 6g). The flexible *n*-pentyl terminus is directed towards the active site entrance and has weak electron density. The indole amine forms a hydrogen bond with Ser238, a residue which forms a water bridge with the A-ring of tetracyclines. The indole also makes several hydrophobic contacts and an edge-to-face π-stacking interaction with Phe319. Remarkably, the Phe224 side chain is rotated through nearly 180° to accommodate the 5-methoxyindole group of tegaserod (ESI Fig. S28†), with which it would sterically clash if it remained in the conformation found in the tigecycline-bound structure. This conformation of Phe224 has not previously been observed in any Tet(X) crystal structures. The guanidine group is positioned to form multiple polar interactions, forming hydrogen bonds with Arg213, the backbone carbonyl of Phe319 and the C4-carbonyl of the FAD isoalloxazine moiety. The latter is of particular interest, as it mimics the binding modes of hydroxyl groups on A and B rings of the tetracycline scaffold to the FAD C4-carbonyl.^8,42,46^ Additionally, the tegaserod guanidine group is positioned to plausibly forms water bridged interactions with Thr59 and the backbone amides of Pro318 and Gly321 which may also contribute to its binding.

### Microbiological studies

To investigate whether biochemical inhibition of Tet(X4) translates to restoration of tetracycline activity against bacterial cells, we tested the compounds against *E. coli* cells expressing Tet(X4). Tet(X4) encoding DNA from the pET28b-(+) expression vector was subcloned into a pBAD vector *via* TOPO cloning. This vector contains the *araBAD* operon, allowing strict control of expression through the addition of l-arabinose. Tigecycline MIC values for commercial TOP10 *E. coli* transformed with pBAD-TOPO-Tet(X4) were found to be dependent on the l-arabinose concentration, with 0.2%_w/v_l-arabinose being optimal for further experiments (ESI Table S4†).

We then performed checkerboard broth microdilution antibiotic susceptibility assays for tigecycline in combination with selected compounds against our model *E. coli* strain (Table 3). Anhydrotetracycline was used as a reference and gave a fractional inhibitory concentration index (FICI) of 0.4 indicating synergy,^64^ consistent with previous reports.^17,20,33^ Tafenoquine, tegaserod, prochlorperazine and trifluoperazine are able to reduce the tigecycline MIC 2–4 fold at high concentrations of inhibitor, though this only corresponds to FICIs between 0.5-1, a value which is indicative of no interaction between the combination.^64^ Note that all four compounds alone possess weak activity against this strain (16–64 μg mL^−1^), consistent with previous reports,^65,66^ meaning that MICs of combinations could be a contribution of both Tet(X) inhibition and antibacterial activity. Checkerboard assays with the same strain without presence of l-arabinose gave identical MICs for the compounds alone but resulted in increases in FICI values (all ≥1, ESI Table S5†), suggesting that Tet(X) inhibition is at least partially involved in restoration of tigecycline activity, although further experiments are necessary to validate this. Comparable antibiotic activity and FICIs in combination with tigecycline were observed for the compounds with a panel consisting of tigecycline resistant *Salmonella*, *E. coli̧ Acinetobacter* and *Proteus* isolates containing *tet(X)* genes (ESI Table S6†). Tegaserod, in particular, displayed a consistent ability to reduce the tigecycline MIC by 2–8 fold in combination against all of the strains tested. Similar results were obtained in combination with tetracycline and doxycycline (ESI Table S7†), although FICIs indicating synergy were not observed.

**Table 3:** Broth microdilution checkerboard assay results for selected Pharmacopeia active compounds and tigecycline against *E. coli* TOP10 containing plasmid pBAD-TOPO-Tet(X4), induced with 0.2%_w/v_l-arabinose

Antibiotic MIC (μg mL^−1^)		Tet(X) inhibitor MIC (μg mL^−1^)		Combination^a^ (antibiotic/inhibitor) MIC (μg mL^−1^)	FICI^b^	Outcome^c^
Tigecycline	4	Anhydrotetracycline	8	0.5/2	0.4	Synergy
		Prochlorperazine	64	2/16	0.8	Indifferent
		Raloxifene	>128	0.5/128	1.1	Indifferent
		Tegaserod	16	1/8	0.8	Indifferent
		Trifluoperazine	64	2/8	0.6	Indifferent
		Tafenoquine	16	2/4	0.8	Indifferent

a Combination MICs reported are the combinations which gave the lowest FICI value.

b FICI was calculated as FIC_antibiotic_ + FIC_inhibitor_, where each FIC = MIC_combination_/MIC_alone_.

c Outcomes are defined by the FICI value as follows: synergy (≤0.5), indifferent (0.5–4.0) or antagonistic (≥4.0).^58^

## Conclusions

Type 1 TDases are a rapidly emerging AMR mechanism with clear clinical consequences, including for last-resort tetracyclines such as tigecycline;^29–32^ however, no strategies to combat TDase resistance have progressed towards clinical development. Although the global spread of TDase-mediated resistance is perhaps not yet at the stage that justifies substantial commercial investment, the history of AMR suggests waiting to implement interventions is a risk. This is evidenced by the dissemination of metallo-β-lactamases, which have progressed within a few decades from an academic curiosity to the point where they threaten the use of all penicillins and cephalosporins.^35,36^ Despite the clinical success of serine β-lactamase inhibitors, analogous inhibitors have not yet been developed for TDases. In part this likely reflects the (to date) relatively rare occurrence of TDase-mediated resistance and in part that the nature of both the tetracyclines and the flavin/O_2_/NADP(H)-dependent TDases are more experimentally challenging than β-lactamases.

To help us and others to identify starting points to progress towards first-in-class TDase inhibitors, we developed and optimised a robust FP-based competition assay using probe 6 that has enabled the discovery of new active-site binding inhibitors of type 1 TDases. A screen of known bioactive compounds utilising our FP assay revealed promising new scaffolds for Tet(X) inhibition. Six structurally-diverse compounds were identified with <100 μM IC_50_ values in both the FP and the UPLC activity assays, providing new scaffolds for inhibitor development. Of the hit compounds, psychoactive phenothiazine derivatives and the 5-HT_4_ agonist tegaserod were co-crystallised successfully with Tet(X4); the resultant structures enabled the discovery of two previously unidentified reversible binding modes of Tet(X4). Importantly, this is the first structural evidence for active site binding of Tet(X) inhibitors that do not share structural similarity with the tetracycline substrates.

These compounds have micromolar potency but do not restore tigecycline activity in bacteria to a level of synergy, hence optimisation will be necessary to improve potency, improve accumulation in bacterial cells, enhance restoration of tigecycline activity in cells and obtain selectivity over their human targets. The latter will be important to eliminate known off-target anti-psychotic^61^ and motility stimulant^60^ activities of the phenothiazines and tegaserod, respectively. Our results further highlight the potential for drug-repurposing approaches to combat AMR to overcome resistance mechanisms, an approach which has shown considerable promise in the discovery of novel antibiotics and modes of action.^67,68^ Note, however, that the bioactive molecules tested, including the phenothiazines and tegaserod, also show antimicrobial activity alone. Taken together with their inhibition of type 1 TDases, the moderate activity of these ‘non-antibiotics’ emphasises the need to evaluate the impact of non-antibiotics on the evolution of AMR.^68^ It may be that long-term treatments with such drugs can promote AMR in often vulnerable patients.

## Materials and methods

### Compound synthesis

Compounds 2–6 were synthesised as described in the ESI.† 9-Bromoanhydrotetracycline was synthesised according to the reported procedure.^33^ All other compounds were purchased from commercial suppliers.

### Recombinant protein purification

TDase genes (Genbank accessions: Tet(X2), AJ311171.1; Tet(X3), KU547176.1; Tet(X4), MK134376.1; Tet(X5), CP044520.1; Tet(X7), KU547185.1; Tet(50), KR857684.1) were synthesised and cloned into the pET28b-(+) vector using restriction enzymes NdeI and BamHI (GenScript, UK). The resulting constructs contained a thrombin-cleavable N-terminal His_6_-tag. For Tet(X2), the full-length protein was prepared rather that the Δ1–10 mutant used previously for crystallographic studies.^42,69^ Plasmids were transformed into chemically-competent *E. coli* BL21(DE3) cells (NEBiolabs) and grown on plates containing 2xYT agar supplemented with kanamycin (30 μg mL^−1^) overnight (37 °C). Single colonies were picked and cultured in 100 mL liquid 2xYT media overnight (37 °C, 180 rpm). Overnight cultures were used to inoculate large-scale growths in 2xYT media (12 × 600 mL, 1 : 100_v/v_) supplemented with kanamycin (30 μg mL^−1^). Cultures were incubated (37 °C, 180 rpm) until an OD_600_ of 0.6–0.8 was recorded. Expression was induced by the addition of isopropyl β-d-1-thiogalactopyranoside (final concentration of 0.1 mM for Tet(X3), 0.5 mM for all other constructs) and cultures were subsequently incubated overnight (18 °C 180 rpm). Cells were harvested by centrifugation (11 325×*g*, 10 min, 4 °C) and cell pellets were stored at −80 °C prior to purification.

Tet(X2) was prepared as reported with minor modifications.^33^ The cell pellet was thawed and resuspended in lysis buffer containing 50 mM K_2_HPO_4_ (pH 8.0), 500 mM NaCl, 20 mM imidazole, 5 mM β-mercaptoethanol and 10%_v/v_ glycerol (100 mL) supplemented with a cOmplete EDTA-free protease inhibitor cocktail tablet (Roche), DNAase I (0.1 mg mL^−1^, Roche), lysozyme (1 mg mL^−1^, Sigma Aldrich) and FAD (1 mg mL^−1^, Sigma Aldrich). Cells were lysed at 25 kPSI using a continuous flow cell disruptor (Constant Systems). Cell debris was removed by centrifugation (58 500×*g*, 30 min, 4 °C) and filtration of the resulting supernatant through a 0.45 μM filter (Sartorius). The filtrate was loaded onto a 5 mL HisTrap HP column (GE Healthcare) previously equilibrated with lysis buffer using an ÄKTApure fast-liquid purification chromatography system (GE Healthcare). The column was washed with lysis buffer (15 CV), then eluted by applying a gradient of 20–300 mM imidazole (12 CV) with the other buffer components kept constant. Fractions containing recombinant protein were identified using SDS-PAGE analysis, concentrated in a 30 kDa molecular weight cut-off centrifugal filter (Merck Millipore), loaded onto a size exclusion column (Superdex 75 HiLoad 26/600, 320 mL, GE Healthcare) and eluted with storage buffer containing 50 mM K_2_HPO_4_ (pH 8.0), 150 mM NaCl, 1 mM DTT (1.2 CV). Fractions containing recombinant protein were pooled and concentrated. Concentrations were estimated using a NanoDrop One spectrophotometer (Thermo Fisher), assuming full occupancy of the FAD cofactor (*ε*_280_ = 22 869 M^−1^ cm^−1^). Protein was flash frozen in single-use aliquots and stored at −80 °C prior to use. Purity was determined by SDS-PAGE analysis. Tet(X3) and Tet(50) were purified using identical conditions to Tet(X2), except using a gradient of 20–500 mM imidazole over 12 CV for elution from the 5 mL HisTrap HP column. Tet(X4) and Tet(X5) were purified as for Tet(X2), except that the lysis buffer contained 20 mM Tris (pH 8.5), 300 mM NaCl, 20 mM imidazole, 2 mM β-mercaptoethanol and the storage buffer contained 20 mM Tris (pH 7.5), 150 mM NaCl and 2 mM β-mercaptoethanol. Tet(X7) was purified identically to Tet(X2), except that the lysis buffer contained 50 mM Tris (pH 8.0), 100 mM NaCl, 20 mM imidazole, 2 mM β-mercaptoethanol and that the storage buffer contained 10 mM Tris (pH 8.0), 150 mM Tris and 2 mM β-mercaptoethanol.

For crystallisation, DNA encoding Tet(X4) was subcloned from the pET28b-(+) construct into a modified pRSETa vector,^70^ incorporating an N-terminally His_6_-tagged lipoyl domain from *B. stearothermophilus* dihydrolipoamide acetyl transferase at the N-terminus of the *tet(X4)* gene, with a TEV protease cleavage site located between the lipoyl domain and the Tet(X4) domain. Overexpression and purification were performed as described for PBP3 ^70^ with minor modifications. The recombinant protein was overproduced in *E. coli* C41(DE3) cells grown in 2xYT media, with expression induced by treatment with 1 mM IPTG overnight (18 °C, 180 rpm). Cells were harvested by centrifugation, resuspended in buffer containing 20 mM Tris (pH 8.0), 100 mM NaCl, 20 mM imidazole, 5 mM β-mercaptoethanol and 1 mg mL^−1^ FAD, then lysed by sonication (30 min, 70% amplitude, pulse programme 2 s on, 7 s off). Following clarification by centrifugation, the lysates were loaded onto a 50 mL Ni-NTA column and eluted with a gradient of 20–500 mM imidazole. The purified protein was digested with His-tagged TEV protease, then dialysed overnight into buffer containing 20 mM Tris (pH 8.0), 100 mM NaCl and 5 mM β-mercaptoethanol. The digestion was passed through a pre-equilibrated Ni-NTA column, removing proteins with an intact His-tag. Tet(X4) was concentrated to 15 mg mL^−1^ in a 30 kDa molecular weight cut-off centrifugal filter (Merck Millipore), flash-frozen in liquid N_2_ and stored at −20 °C prior to crystallisation.

### Tetracycline destructase binding assays

Aliquots of TDases were thawed and serially diluted with the assay buffer (100 mM Tris, pH 7.0 with 0.01% Triton X-100) in a V-bottom 96-well plate (Greiner Bio-One). 12.5 μL of each concentration of enzyme was transferred in quadruplicate to a 384-well black, non-binding microtiter plate (Greiner Bio-One). Aliquots of 2 mM stock of fluorescent probes 5 and 6 in DMSO were thawed and diluted to 50 nM in the assay buffer with 2 μM FAD and 10 mM MgCl_2_. 12.5 μL of this solution was added to wells containing enzyme. A negative control of 12.5 μL of the probe solution with 12.5 μL of the assay buffer was included on each plate. The plate was briefly centrifuged (1000 rpm), then incubated at ambient temperature for 30 min before measuring the FP response using a PHERAstar FS microplate reader (BMG Labtech) equipped with an FP optic module (excitation = 540 ± 20 nm, Emission = 590 ± 20 nm, 200 flashes per well). ΔmP values were calculated by subtracting the average mP of the negative controls from the mP measured for each well. To calculate *K*_d_ values, the derived ΔmP values were plotted as a function of enzyme concentration and a one-site specific binding model was applied (GraphPad Prism 9.4.1). To assess the effect of MgCl_2_ on the *K*_d_ of the interaction between the probe and Tet(X4), the above procedure was repeated with the probe solution containing 50 nM probe 6 and 2 μM FAD with or without the addition of 10 mM MgCl_2_ in the Tet(X4) enzyme solution.

### Competitive displacement experiments

A 2× solution containing 80 nM Tet(X4), 2 μM FAD and 10 mM MgCl_2_ in the assay buffer was dispensed into the first 22 columns of a 384-well black, non-binding microtiter plate (Greiner Bio-One), 12.5 μL per well, using a microplate reagent dispenser. A solution of 2 μM FAD and 10 mM MgCl_2_ in the assay buffer was dispensed into the remaining columns to serve as a no enzyme control. A 3-fold, 10-point dilution series of substrates and inhibitors was made in DMSO in a V-bottom 96-well plate (Greiner Bio-One) with a highest concentration of 5 mM or 0.5 mM. Into one column was added DMSO with no inhibitor or substrate, to serve as a positive control. Tigecycline was included as a reference on every plate to ensure reproducibility. 0.5 μL of each dilution was transferred in quadruplicate to the 384-well plate containing enzyme solution using a CyBi-Well liquid-handling robot (CyBio). A solution of 50 nM 6 in the assay buffer was then dispensed, with 12.5 μL per well. The plate was briefly centrifuged (1000 rpm), then incubated at ambient temperature for 30 min prior to measuring the FP. Data points were normalised between the positive and negative controls. Normalised % response was plotted as a function of log_10_([Inhibitor]), and the IC_50_^app^ was determined by fitting the data to the equation: Y = Bottom + (top-bottom)/(1 + 10^Log(IC50)^ − X) × HillSlope in GraphPad Prism 9.4.1, where Y = % response and X = log_10_([Inhibitor]). Wells with unusual fluorescence intensities were manually identified and removed from the analysis.

For screening of the Pharmacopeia library, the above method was used with minor modifications. Compounds were dispensed into assay plates using a LabCyte Echo 650, 25 nL per well (final DMSO concentration = 0.1%_v/v_). The Tet(X4) solution concentration was increased to 160 nM (final concentration in well = 80 nM). The 0% response was defined as the response with addition of 10 μM tigecycline. The final concentration for all compounds screened was 10 μM. One technical replicate was performed for each compound; hits identified were retested; the purities of the hit compounds were determined to be >80% by LC-MS analysis.

### Optimisation of assay parameters

Optimisation of assay parameters (DMSO %_v/v_, pH and incubation time) was performed using the above method with modifications. The positive controls contained final concentrations of 25 nM probe 6, 40 nM Tet(X4), 1 μM FAD and 5 mM MgCl_2_ in the assay buffer, 25 μL final volume, whilst varying the parameter to be optimised. Negative controls contained the same solution with the addition of 10 μM tigecycline. Detailed methodology can be found in the ESI.†

### UPLC activity assays

A solution containing 100 mM TAPS (pH 8.5), 20 μM FAD, 10 mM MgCl_2_ and 100 nM Tet(X4) was dispensed into a 96-well skirted PCR plate (Sarstedt), 50 μL per well, using a microplate reagent dispenser. A 3-fold, 10-point dilution series of the substrates and inhibitors was made in DMSO with a top concentration of 10 mM, unless otherwise stated, and transferred to the enzyme solution using a CyBi-Well liquid-handling robot (CyBio), 1 μL per well. On each plate, a column of no enzyme negative controls and no inhibitor (DMSO only) positive controls were included. The plate was incubated at 30 °C for 15 min. A solution containing 100 mM TAPS (pH 8.5), 40 μM tigecycline and 200 μM NADPH was then dispensed, with 49 μL per well. The plate was incubated at 30 °C for 20 min at 600 rpm. 10 μL of 10%_v/v_ formic acid solution was then dispensed into all wells. Samples were analysed using an ACQUITY H-Class PLUS UPLC instrument (Waters) equipped with a pre-equilibrated ACQUITY BEH C18 column (20 × 50 mm, 1.7 μM pore size, Waters) using a gradient of 1–50%_v/v_ acetonitrile with 0.1%_v/v_ formic acid in water with 0.1%_v/v_ formic acid over 1.83 minutes at a flow rate of 0.5 mL min^−1^. Instrument control and data processing were performed using MassLynx V4.1 software. The peaks representing tigecycline and 11a-hydroxy-tigecycline were integrated, used to calculate % conversion and then normalised between positive and negative controls. The normalised % response was plotted as a function of log_10_[Inhibitor], and the IC_50_ determined as described above.

### Crystallisation

An aliquot of the Tet(X4) crystallography construct was thawed and stored on ice prior to set up of crystallisation trays. Tet(X4) at 15 mg mL^−1^ was mixed with each inhibitor dissolved in DMSO, with a final concentration of 2–5 mM inhibitor and 2%_v/v_ DMSO. Sitting-drop crystal plates were dispensed using a Mosquito Xtal3 machine (SPT Labtech) in 300 nL drops. Well solutions were used as purchased from Hampton Research or Molecular Dimensions. Co-crystals with prochlorperazine formed with a well solution containing 27 %_w/v_ PEG3350, 0.1 M bis–tris propane (pH 7.0), 0.2 M lithium sulfate. Co-crystals with trifluoperazine formed with a well solution containing 29 %_w/v_ PEG4000, 0.1 M sodium citrate (pH 6.5), 0.1 M magnesium acetate, 0.1 M ammonium sulfate. Co-crystals with tegaserod formed with a well solution containing 30%_w/v_ PEG4000, 0.2 M ammonium acetate, 0.1 M sodium citrate (pH 5.6). Prior to harvesting, crystals were cryoprotected by addition of 600 nL of 15 %_v/v_ glycerol in the mother liquor to the drops. Single crystals were harvested with nylon loops and flash frozen in liquid nitrogen. X-ray datasets were collected at Beamline I03 or I04 at Diamond Light Source, United Kingdom. Diffraction data were integrated and scaled using xia2 with DIALS.^71^ Tet(X4) crystals were in the *P*6_5_22 space group with one copy per asymmetric unit, consistent with previous reports.^8^ Structures were solved by molecular replacement with Phaser using the reported *apo*-Tet(X4) structure (PDB: 7EPV) as the search model. Iterative rounds of refinement in Phenix^72^ and manual model building in Coot^73^ were performed until convergence of the *R*_free_ and *R*_work_ was observed. Data collection and refinement statistics are given in ESI Table S3.†

### Construction of a model Tet(X4)-bearing *E. coli* strain

Tet(X4) was sub-cloned from the Tet(X4) pET28b-(+) expression vector *via* TOPO cloning. The insert was amplified using the taq polymerase with the forward primer 5′- ATGAGCAATAAAGAAAAACAAATGAATTTACTTAGTG-3′ and the reverse primer 5′- TTATACATTTAACAATTGCTGAAACGTAAAGTCG-3’. Following PCR purification (Thermo Fisher Scientific), the A-tailed PCR product was ligated into the pBAD-TOPO vector using a TOPO cloning kit (Invitrogen) according to the manufacturer's protocol. Following amplification in *E. coli* NEB5α cells (NEBiolabs), the fidelity of the insert was confirmed by Sanger sequencing (Eurofins). For microbiological experiments, *E. coli* TOP10 cells were used as the recipient strain. Cells were plated on Mueller-Hinton agar (MHA) plates containing ampicillin at 100 μg mL^−1^. The introduction of pBAD-TOPO-Tet(X4) was confirmed by colony PCR. Strains were stored in a freezer at −80 °C in cryopreservation beads.

### Antibiotic susceptibility assays

MIC tests and checkerboard assays were performed in U-bottom, 96-well microtiter plates using a modified broth microdilution protocol following CLSI guidelines. Cation-adjusted Mueller-Hinton Broth (CAMHB) was prepared fresh on the day of testing.^74^ In brief, assays were set up in a total volume of 100 μL per well and titrations comprised seven 2-fold dilutions of inhibitors and ten 2-fold dilutions of tigecycline in CAMHB. Bacterial colonies were selected from an overnight culture on MHA plates (supplemented with 0.2%_w/v_l-arabinose for *E. coli* TOP10 containing pBAD-TOPO-Tet(X4)) and were suspended in 0.9% saline solution to achieve a 0.5 McFarland standard. A 100 μL aliquot of this suspension was then diluted into 9.9 mL of CAMHB to yield approximately 1.5 × 10^6^ cfu mL^−1^. 50 μL of the suspension was then added to each well of a microtiter plates. Plates were incubated at 37 °C for 18 h. At least 2 replicates were conducted for each combination. *E. coli* TOP10 + pBAD-TOPO-Tet(X4) cells were grown in the absence and presence of 0.2%_w/v_l-arabinose. Analysis of the MIC and checkerboard results were carried out by visual inspection of growth, with and without resazurin dye, an oxidation–reduction indicator.^75^ As a measure of synergy, fractional inhibitory concentration index (FICI) was calculated as FIC_A_ + FIC_B_, where FIC_A_ and FIC_B_ are the MIC_combination_/MIC_alone_ for each agent, respectively.^76^ Results were interpreted as follows: synergistic (<0.5), indifferent (1.0–4.0) or antagonistic (>4.0).^64^ In cases where the MIC exceeded or was below the measured range, the borderline value was taken to calculate the FIC index.

### Statistical analyses

All *K*_d_, IC_50_ and IC_50_^app^ values are presented as the mean value from three independent experiments with errors representing the standard deviation from the mean. All enzymatic experiments were performed in at least technical quadruplicate on the same plate, apart from UPLC-based assays which were conducted with one technical replicate.

## Data availability

The authors declare that all relevant data that supports the findings of this study are available within the paper and its ESI.† Crystallographic data supporting the findings of this study are available in the Protein Data Bank (PDB) with accession codes: 9HKE (trifluoperazine-Tet(X4) complex), 9HJV (prochlorperazine-Tet(X4) complex) and 9HJW (tegaserod-Tet(X4) complex). Requests for further materials or data should be made to Christopher J. Schofield (christopher.schofield@chem.ox.ac.uk).

## Author contributions

M. J. B. produced proteins, developed and performed biochemical assays with assistance from H. G. S, Z. B. and V. G.; E. C. T. performed chemical synthesis; H. G. S. collected and processed crystallographic data with assistance from M. J. B.; M. M. T. performed microbiological experiments with assistance from M. J. B.; J. H. J. A., M. Y. W. W. and C. H. J. W. performed screening of the pharmacopeia library; H. S. A. performed molecular docking; M. J. B., E. C. T., A. J. M. F. and C. J. S. conceptualised and designed the study; M. J. B., E. C. T., A. J. M. F., F. D., T. R. W. and C. J. S. supervised the study; M. J. B. and C. J. S. wrote the manuscript with input from all other authors.

## Conflicts of interest

The authors declare that there are no conflicts of interests.

## Supplementary Material

SC-016-D5SC00964B-s001
