# Supplementary material for: Binding assays enable discovery of Tet(X) inhibitors that combat tetracycline destructase resistance
Source: Chem Sci. 2025 May 7;16(22):9691–704. doi: 10.1039/d5sc00964b (PMC12056667; doi:10.1039/d5sc00964b)
Supplement: SC-016-D5SC00964B-s001 [file SC-016-D5SC00964B-s001.pdf]

Supplementary Information (SI) for Chemical Science.
This journal is © The Royal Society of Chemistry 2025

Binding Assays Enable Discovery of Tet(X) Inhibitors that Combat Tetracycline Destructase Resistance

Matthew J. Beech[a], Edmond C. Toma[a], Helen G. Smith[a], Maria M. Trush[b], Jit H. J. Ang[c], Mei Y. Wong[c], Chung H. J. Wong[c], Hafiz S. Ali[a], Zakia Butt[a], Viha Goel[a], Fernanda Duarte[a], Alistair J. M. Farley[a], Timothy R. Walsh[b], and Christopher J. Schofield[a*]

[a] Chemistry Research Laboratory, Department of Chemistry and the Ineos Oxford Institute for Antimicrobial Research, University of Oxford, Oxford, OX1 3TA, United Kingdom.

[b] Department of Biology and the Ineos Oxford Institute for Antimicrobial Research, University of Oxford, Oxford, OX1 3RE, United Kingdom.

[c] Experimental Drug Development Centre (EDDC), Agency for Science, Technology and Research (A*STAR), 10, Biopolis Road, Singapore 138670, Singapore.

Table of Contents

Supplementary Methods 3
General Procedures and Materials 3
Chemical Synthesis 4
Biochemical Experiments 9
Supplementary Figures 14
Figure S1 14
Figure S2 15
Figure S3 16
Figure S4 17
Figure S5 18
Figure S6 19
Figure S7 20
Figure S8 21
Figure S9 22
Figure S10 23
Figure S11 24
Figure S12 25
Figure S13 26
Figure S14 27
Figure S15 28
Figure S16 29
Figure S17 30
Figure S18 32
Figure S19 33
Figure S20 34
Figure S21 36
Figure S22 38
Figure S23 39
Figure S24 40
Figure S25 42
Figure S26 43
Figure S27 44
Figure S28 45
Supplementary Tables 46
Table S1 46
Table S2 49
Table S3 51
Table S4 52
Table S5 53
Table S6 54
Table S7 55
NMR Spectra 56
UPLC-MS Chromatograms 63
References 67

Supplementary Methods

General Procedures and Materials

Reagents and solvents for chemical synthesis were used as received from commercial suppliers (Merck Life Science UK Ltd., Sigma-Aldrich Inc., Biosynth Ltd., Fluorochem Ltd., Activate Scientific GmbH, Apollo Scientific Ltd., and Alfa Aesar Inc.). A Biotage Isolera One was used for flash silica gel chromatography with pre-packed Biotage® Sfär C18 D – Duo 100 Å 30 µM flash chromatography cartridges using HPLC grade solvents. Melting points (MP) were determined by a Stuart SMP-40 automated melting point apparatus. High-resolution mass spectrometry (HRMS) employed a BioAccord Mass Spectrometer (Waters Corp.), using electrospray ionization (ESI) mass spectrometry in the positive ionization mode. Data are presented as a mass-to-charge ratio (*m*/z) and are calculated to 4 decimal places from the molecular formula to be within a measured tolerance of 5 ppm. Proton (^{1}H) and carbon (^{13}C) nuclear magnetic resonance (NMR) spectroscopy were recorded using a Bruker AVIII400 (400/101 MHz) or Bruker AVIII500 (500/126 MHz) machines. Chemical shifts (δ_H and δ_C) are reported as parts per million (ppm), referenced to the residual peak stated (DMSO-d_6 (δ = 2.50/39.5)) with peak splitting recorded as singlet (s), broad singlet (bs), doublet (d), triplet (t), quartet (q), quintet (quin), sextet (sex), septet (sept) and multiplet (m). Coupling constants (*J*) are reported to the nearest 0.5 Hz. The purity of final compounds was determined using an Acquity UPLC (Waters) instrument equipped with an XBridge® BEH C18 2.5 µM 2.1 x 50 mm column. A mixture of MeCN:H_2O with 0.1%$_{v/v}$ formic acid additive was used for the gradient of 5%$_{v/v}$ MeCN for 0.1 min at 0.5 mL/min, then ramped to 95%$_{v/v}$ MeCN over 2.9 min at 0.8 mL/min, held at 95%$_{v/v}$ MeCN for 0.5 min at 0.8 mL/min, and then decreased to 5%$_{v/v}$ MeCN over 0.5 min at 0.8 mL/min. The spectra were acquired at 280 nm with integrations.

Chemicals used for preparation of buffers and solutions for biochemical experiments were from commercial suppliers and were used without further purification. Ultrapurified water (>18 MΩ cm) was obtained from a PURELAB Chorus 1 Complete (ELGA Labwater) and was used to make all solutions. Dispensing of reagents into microplates was conducted using either a Multidrop™ Combi dispenser

(Thermo Fisher) equipped with a small tube, metal tipped cassette or an WELLJET dispenser (Integra Biosciences) equipped with an 8-channel cassette.

Chemical Synthesis

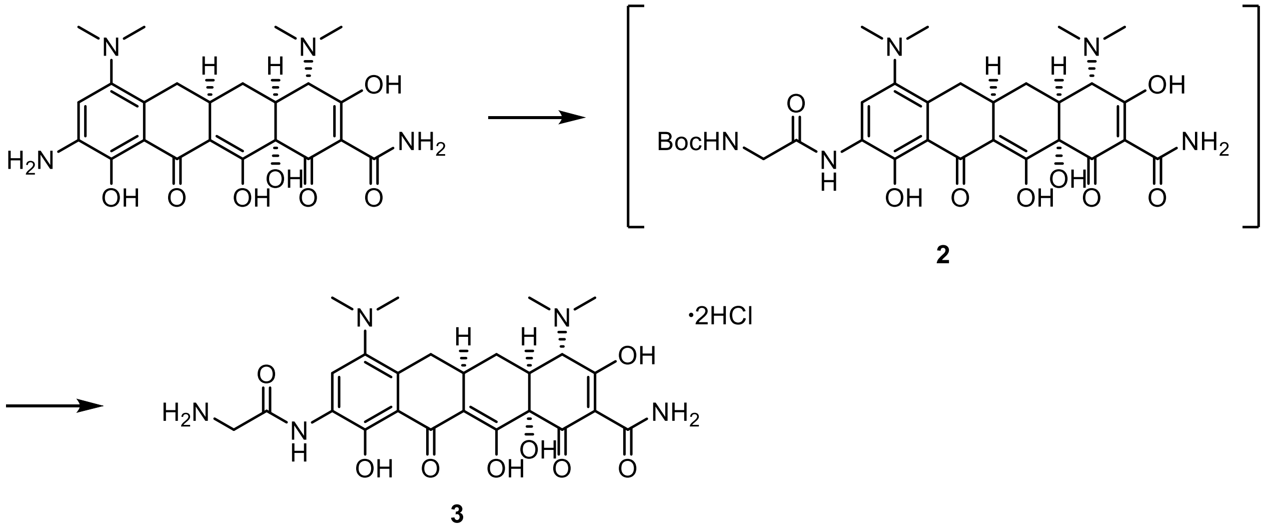

(4*S*,4a*S*,5a*R*,12a*S*)-9-[(Aminoacetyl)amino]-4,7-bis(dimethylamino)-3,10,12,12a-tetrahydroxy-1,11-dioxo-1,4,4a,5,5a,6,11,12a-octahydronaphthacene-2-carboxamide dihydrochloride salt (3)[1]

Intermediate **2** was prepared according to a modified version of the procedure of Chen *et al.*[1] To a solution of (*tert*-butoxycarbonyl)glycine (0.17 g, 0.79 mmol, 1.3 equiv.) and 1-[bis(dimethylamino)methylene]-1H-1,2,3-triazolo[4,5-b]pyridinium 3-oxide hexafluorophosphate (HATU, 0.33 g, 0.86 mmol, 1.1 equiv.) in anhydrous DMF (2.2 mL) was added *N*,*N*-diisopropylethylamine (DIPEA, 0.21 mL, 1.2 mmol, 1.5 equiv.) dropwise under an N_2 atmosphere. After stirring for 30 min, a solution of 9-amino minocycline·hydrochloride (0.40 g, 0.79 mmol, 1.0 equiv.) in anhydrous DMF (3.0 mL) was added dropwise. The reaction was stirred for 2 hr; the product was precipitated by addition of Et_2O (15 mL) that had been cooled to -10 °C. The precipitate was immediately filtered using a funnel jacket to maintain a temperature of -10 °C; the resultant yellow-orange precipitate was washed with Et_2O (2 × 10 mL) that had been cooled to -10 °C. **2** was carried forward to the next step without further purification. HRMS (ESI$^+$) calc. for $C_{30}H_{40}N_5O_{10}$ ([M+H]$^+$) 630.2770; found 630.2790.[1]

Synthesis of **3** employed a modified version of the procedure of Chen *et al.*[1] To a round bottom flask containing intermediate **2** at 0 °C, 4N HCl in dioxane (3.0 mL) was added slowly until the solid was fully dissolved. The solution was brought to room temperature and stirred for 30 min. Following complete removal of the Boc group, the mixture was cooled to -10 °C and precipitated by the addition of Et_2O (15 mL) that had been cooled to -10 °C. The precipitate was immediately filtered using a funnel jacket to maintain a temperature of -10 °C; the resultant yellow precipitate was washed with Et_2O (2 × 10 mL) that had been cooled to -10 °C. The product was dried *in vacuo* to afford 9-glycylamido minocycline dihydrochloride (**3**) as a bright yellow solid (0.48 g, 0.75 mmol, 95%, 4:1 (4(*S*):4(*R*))).[1]

MP: 186-187 °C. **HRMS** (ESI^+) calc. for $C_{25}H_{32}N_5O_8$ ($[M+H]^+$) 530.2250; found 530.2245.[1] **^{1}H NMR** (500 MHz, DMSO-d_6, 2 diastereomers, only reported major diastereomer where possible) δ_H 14.64 (s, 1H), 12.14 (s, 1H), 10.55 (s, 1H), 10.16 (s, 1H), 9.57 (s, 1H), 9.05 (s, 1H), 8.46 – 8.24 (m, 5H), 4.36 (s, 1H), 3.88 (m, 3H), 3.10 – 2.98 (m, 2H), 2.98 – 2.67 (m, 16H), 2.39 – 2.17 (m, 3H), 1.48 (apparent td, *J* = 13.6, 11.0 Hz, 1H). **^{13}C NMR** (126 MHz, DMSO-d_6) δ_C 171.9, 165.7, 131.8, 120.6, 115.9, 108.3, 95.2, 74.0, 67.8, 45.0, 41.4, 40.9, 34.4, 34.0, 31.5, 29.5.

Analytical data were in accord with those reported by *Chen et al.*[1]

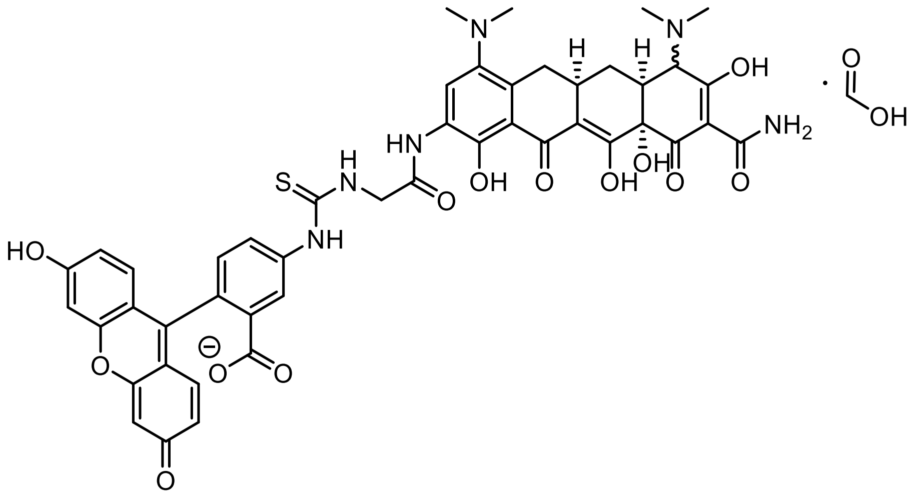

***N*-(2-{[(5a*R*,6a*S*,7*R*,10a*S*)-9-Carbamoyl-4,7-bis(dimethylamino)-1,8,10a,11-tetrahydroxy-10,12-dioxo-5,5a,6,6a,7,10,10a,12-octahydronaphthacen-2-yl]amino}-2-oxoethyl)-*N*'-(3',6'-dihydroxy-3-oxo-3*H*-spiro[[2]benzofuran-1,9'-xanthene]-5-yl)thiourea formate salt (4)**

To a solution of **3** (100 mg, 0.166 mmol, 1.00 equiv.) in anhydrous DMF (1.0 mL) , triethylamine (53.0 µL, 0.380 mmol, 2.29 equiv.) was added dropwise under a N_2 atmosphere at 0 °C. After stirring for 5 min, a solution of fluorescein-5-isothiocyanate (FITC, 116 mg, 0.90 mmol, 2.29 equiv.) in anhydrous DMF (0.6 mL) was added dropwise to the reaction mixture at 0 °C. The reaction was warmed up to rt and stirred for 2 hr. The solution was concentrated using a stream of N_2 and purified by flash C18 column chromatography (MeCN:aq. 0.1%$_{v/v}$ formic acid, 20%$_{v/v}$ MeCN for 3 column volumes (CV), then a gradient of 20-95%$_{v/v}$ MeCN over 15 CV) affording **4** (119 mg, 0.123 mmol, 74% yield) as a dark yellow solid.

MP 197-199 °C Decomposition. **HRMS** (ESI$^+$) calc. for $C_{46}H_{43}N_6O_{13}S$ ([M+H]$^+$) 919.2603; found 919.2641. **^{1}H NMR** (400 MHz, DMSO-*d*$_6$, observable peaks) δ_H 10.36 (s, 1H), 10.11 (s, 2H), 9.51 (s, 1H), 8.36 – 8.24 (m, 2H), 8.14 (s, 1H), 7.78 (d, *J* = 8.5 Hz, 1H), 7.22 (d, *J* = 8.0 Hz, 1H), 6.67 (d, *J* = 2.5 Hz, 2H), 6.62 (d, *J* = 8.5 Hz, 2H), 6.56 (dd, *J* = 8.5, 2.5 Hz, 2H), 4.46 (s, 2H), 3.19 – 3.09 (m, 1H), 3.04 – 2.98 (m, 1H), 2.84 (s, 1H), 2.54 (s, 12H), 2.45 (s, 6H), 2.15 – 2.04 (m, 1H), 1.64 – 1.56 (m, 1H), 1.18 – 1.05 (m, 2H).

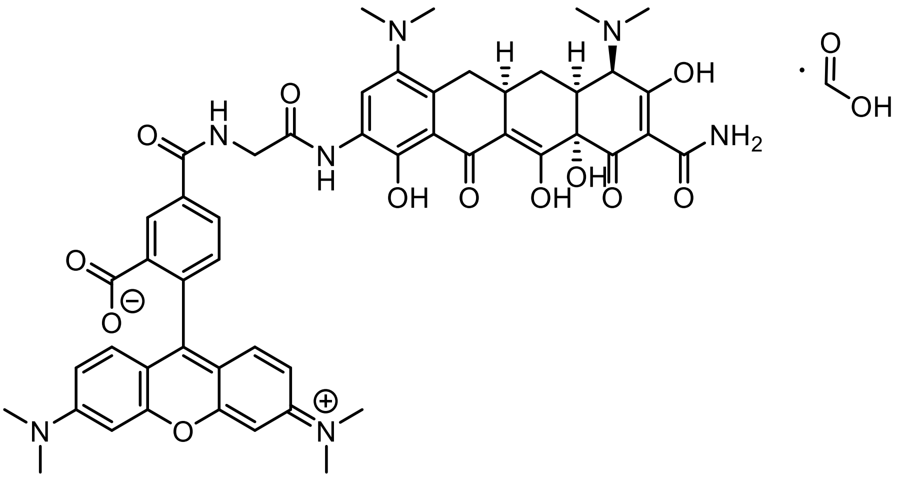

5-[(2-{[(5a*R*,6a*S*,7*R*,10a*S*)-9-Carbamoyl-4,7-bis(dimethylamino)-1,8,10a,11-tetrahydroxy-10,12-dioxo-5,5a,6,6a,7,10,10a,12-octahydronaphthacen-2-yl]amino}-2-oxoethyl)carbamoyl]-2-[6-(dimethylamino)-3-(dimethylazaniumylidene)-3*H*-xanthen-9-yl]benzoate formate salt (5)

To a solution 5-carboxytetramethylrhodamine (5-TAMRA, 20 mg, 47 µmol, 1.0 equiv.) and HATU (11 mg, 47 µmol, 1.0 equiv.) in anhydrous DMF (0.1 mL) was added DIPEA (16 µL, 93 µmol, 2.0 equiv.),

dropwise under a N_2 atmosphere. After stirring for 1 hr, a solution of **3** (39 mg, 70 µmol, 1.5 equiv.) in anhydrous DMF (0.1 mL) was added dropwise. The reaction was stirred for 16 hr, then concentrated under a stream of N_2. Purification by flash C18 column chromatography (MeCN:aq. 0.1%$_{v/v}$ formic acid, 5% $_{v/v}$ MeCN for 3 CV, then a gradient of 5-95% $_{v/v}$ MeCN over 15 CV) afforded **5** (20 mg, 20 µmol, 44% yield) as a purple solid.

MP: 210-211 °C Decomposition. **HRMS** (ESI$^+$) calc. for $C_{50}H_{51}N_7O_{12}Na$ ([M+Na]$^+$) 964.3488; found 964.3446. **^{1}H NMR** (400 MHz, DMSO-*d*$_6$, observable peaks) δ_H 9.44 (d, *J* = 8.5 Hz, 1H), 9.25 (s, 1H), 8.52 (s, 1H), 8.28 (dd, *J* = 8.0, 1.5 Hz, 1H), 8.15 (d, *J* = 6.0 Hz, 1H), 7.37 (d, *J* = 8.0 Hz, 1H), 6.59 – 6.44 (m, 6H), 4.21 (d, *J* = 6.0 Hz, 2H), 3.23 – 3.11 (m, 2H), 2.96 (s, 12H), 2.89 – 2.69 (m, 3H), 2.54 (s, 6H), 2.46 (s, 3H), 2.23 – 2.07 (m, 2H), 1.61 (s, 1H), 1.43 – 1.31 (m, 1H).

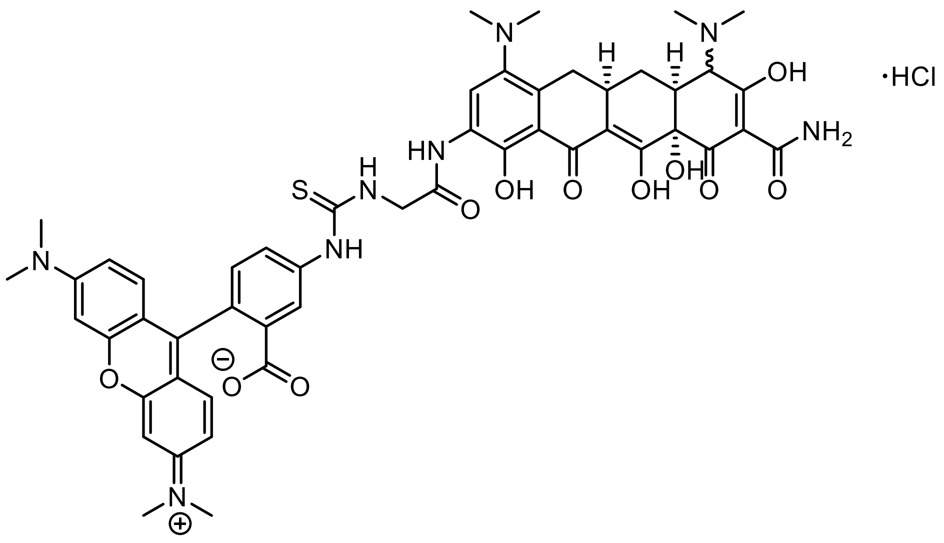

5-{[(2-{[(5a*R*,6a*S*,10a*S*)-9-Carbamoyl-4,7-bis(dimethylamino)-1,8,10a,11-tetrahydroxy-10,12-dioxo-5,5a,6,6a,7,10,10a,12-octahydronaphthacen-2-yl]amino}-2-oxoethyl)carbamothioyl]amino}-2-[6-(dimethylamino)-3-(dimethylazaniumylidene)-3*H*-xanthen-9-yl]benzoate hydrochloride salt (6)

To a solution of **3** (28 mg, 50 µmol, 1.1 equiv.) in anhydrous DMF (0.5 mL) was added DIPEA (31 µL, 180 µmol, 4.0 equiv.) dropwise under a N_2 atmosphere at 0 °C. After stirring for 5 min, a solution of tetramethylrhodamine-5-isothiocyanate (5-TRITC, 20 mg, 45 µmol, 1.0 equiv.) in anhydrous DMF (0.6 mL) was added dropwise into the reaction at 0 °C. The reaction was warmed up to rt and stirred for 2

hr. The solution was loaded directly onto a C18 column and purified by flash C18 column chromatography (MeCN:aq. 0.005N HCl, 5% MeCN for 3 CV, then → 95% MeCN over 15 CV) affording **6** (29 mg, 29 µmol, 64%, 1:1 (4(*S*):4(*R*))) as a purple solid.

MP: 207-209 °C Decomposition. **HRMS** (ESI$^+$) calc. for $C_{50}H_{53}N_8O_{11}S$ ([M+H]$^+$) 973.3549; found 973.3545. **^{1}H NMR** (400 MHz, DMSO-*d*$_6$, observable peaks, 2 diastereomers, separated diastereomer where possible) δ_H 13.12 (bs, 1H), 11.85 – 11.67 (m, 1H), 11.00 – 10.77 (m, 1H), 9.54 (s, 2H), 9.32 (s, 1H), 8.54 (s, 2H), 8.10 – 8.27 (m, 2H), 7.61 – 6.80 (m, 14H), 6.64 (s, 2H), 4.75 (s, 1H), 4.49 (s, 2H), 4.27 (s, 1H), 3.27 (s, 24H), 3.13 – 2.64 (m, 13H), 2.63 – 2.52 (m, 12H), 2.41 – 2.14 (m, 4H), 1.75 (s, 4H), 1.58 – 1.38 (m, 2H).

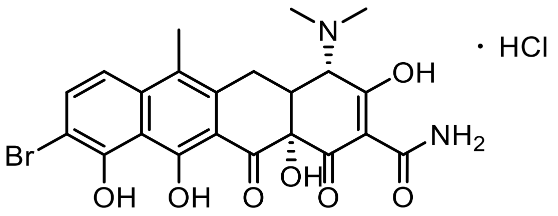

(4*S*,12a*S*)-9-Bromo-4-(dimethylamino)-3,10,11,12a-tetrahydroxy-6-methyl-1,12-dioxo-1,4,4a,5,12,12a-hexahydronaphthacene-2-carboxamide (9-Bromo-anhydrotetracycline, 9-Br-aTC)[2]

9-Bromo-anhydrotetracycline was prepared according to a modified procedure of that of Markley *et al.*[2] To a solution of trifluoroacetic acid (2.0 µL, 26 µmol, 0.12 equiv.) in acetic acid (10 mL), bromine (13 µL, 0.26 mmol, 1.2 equiv.) was added in a single portion under a N_2 atmosphere. The mixture was heated to 50 °C, after which time anhydrotetracycline hydrochloride (100 mg, 0.22 mmol, 1.0 equiv.) was added in one portion. The reaction mixture was stirred at 50 °C for 30 min, and then cooled to room temperature where the reaction mixture was stirred for a further 4 hr. The crude mixture was then concentrated *in vacuo* and reconstituted with aqueous 1 N HCl. The solution was concentrated *in vacuo* affording 9-bromo-anhydrotetracycline (9-Br-aTC, 92 mg, 0.22 mmol, 79% yield) as an orange-yellow solid.

MP: 202-203 °C. **HRMS** (ESI$^+$) calc. for $C_{22}H_{22}BrN_2O_7$ ([M+H]$^+$) 505.0605; found 505.0615. **^{1}H NMR** (400 MHz, DMSO-d_6) δ_H 9.61 (s, 1H), 9.23 (s, 1H), 7.84 (d, J = 9.0 Hz, 1H), 7.42 (d, J = 9.0 Hz, 1H), 4.35 (s, 1H), 3.51 (dd, J = 17.5, 4.5 Hz, 1H), 3.43 – 3.38 (m, 1H), 3.12 – 3.01 (m, 1H), 2.88 (s, 6H), 2.40 (s, 3H). **^{13}C NMR** (101 MHz, DMSO-d_6) δ_C 200.2, 192.7, 187.9, 172.1, 162.1, 153.6, 137.8, 135.7, 131.7, 122.2, 116.5, 112.7, 109.3, 104.4, 97.7, 76.3, 66.8, 42.1, 36.0, 14.0.

Analytical data were in accord with those reported by Markley *et al.*[2]

Biochemical Experiments

DMSO Tolerance Experiments

The assay buffer containing 66.7 nM Tet(X4), 8.33 mM $MgCl_2$ and 1.67 µM FAD, 15 µL per well, for the positive control, was dispensed into 8 rows of a 384-well black, non-binding microtiter plate (Greiner Bio-One). The assay buffer containing 66.7 nM Tet(X4), 8.33 mM $MgCl_2$, 1.67 µM FAD and 16.7 µM tigecycline, 15 µL per well, for the negative control, was dispensed into a separate 8 rows. A no enzyme control containing 8.33 mM $MgCl_2$ and 1.67 µM FAD in assay buffer was manually pipetted in quadruplicate into empty wells in the plate. A DMSO dilution series was prepared in a V-bottom 96-well plate (Greiner Bio-One) by diluting DMSO in the assay buffer (100 mM Tris, pH 7.0 with 0.01% Triton X-100) to five-fold of the final desired concentration. The dilution series was then transferred in twelve technical replicates, 5 µL per well, for each control using an Integra VIAFLO 16-channel electronic pipette. A solution of 125 nM probe (**6**) in the assay buffer was then dispensed into all of the wells, 5 µL per well. The plate was briefly centrifuged (1000 rpm), then incubated at ambient temperature for 30 mins prior to measuring the FP using a PHERAstar FS microplate reader (BMG Labtech) equipped with a TAMRA-wavelength FP optic module (Excitation = 540 ± 20 nM, Emission = 590 ± 20 nm, 200 flashes per well). ΔmP values were calculated by subtracting the average mP of the negative controls from the mP measured for each well. Z′ factors for each DMSO % was calculated using the equation: Z′ factor = $1 - (3(\sigma^+ + \sigma^-)/(\mu^+ - \mu^-))$, where σ^+ and σ^- are the standard deviations for the positive and negative controls, respectively, and μ^+ and μ^- are the means for positive and negative

controls, respectively. Means and standard deviations for the Z′ factor were calculated from three independent experiments.

pH Tolerance Experiments

Solutions containing 100 mM MES (for pH 5.5, 6.0 and 6.5) and 100 mM Tris (for pH 7.0, 7.5, 8.0, 8.5 and 9.0) buffers were prepared and 0.01%$_{v/v}$ Triton X-100 was added. Each buffer solution was dispensed into 2 rows of a 384-well black, non-binding microtiter plate (Greiner Bio-One), with 20 µL per well. A solution containing 2.5 µM Tet(X4), 10 µM FAD and 50 mM $MgCl_2$ in assay buffer was dispensed into 11 columns of the plate (2.5 µL per well) as the positive control. The same solution with 100 µM tigecycline in assay buffer was dispensed into 11 separate columns (2.5 µL per well) for the negative control. Into the remaining two columns, a solution containing 10 µM FAD and 50 mM $MgCl_2$ was dispensed. A solution of 250 nM probe in assay buffer was dispensed into all of the wells (2.5 µL per well). The plate was briefly centrifuged (1000 rpm), then incubated at ambient temperature for 30 mins prior to measuring the FP. ΔmP and Z′ factor values were calculated as described above. Means and standard deviations for the Z′ factor were calculated from three independent experiments.

Time Tolerance Experiments

The assay buffer containing 66.7 nM Tet(X4), 8.33 mM $MgCl_2$ and 1.67 µM FAD, was dispensed into 11 columns of a 384-well black, non-binding microtiter plate (Greiner Bio-One), (15 µL per well). The same solution with 16.7 µM tigecycline (15 µL per well) was dispensed into a separate 11 columns. Into the remaining 2 columns was dispensed assay buffer containing only 8.33 mM $MgCl_2$ and 1.67 µM FAD (15 µL per well) as no enzyme controls. A solution of assay buffer containing 62.5 µM probe **6** was dispensed into all wells (10 µL per well). The plate was briefly centrifuged (1000 rpm), then the FP response was measured at the stated timepoints (Fig. 4C). Between each timepoint, the plate was covered with a low-evaporation lid and incubated at room temperature. ΔmP and Z′ factor values were calculated as described above. Means and standard deviations for the Z′ factor and assay window were calculated from three independent experiments.

Absorbance Activity Assays

A solution containing 100 mM TAPS (pH 8.5), 2 μM FAD, 10 mM $MgCl_2$ and 100 nM Tet(X4) was dispensed into a 384 well μClear plate (Greiner Bio-One), 25 μL per well, using a microplate reagent dispenser. A 3-fold, 10-point dilution series of the substrates and inhibitors was made in DMSO with the highest concentration of 50 mM, unless otherwise stated, and transferred to the enzyme solution in technical quadruplicate using a CyBi-Well (CyBio) liquid-handling robot (1 μL per well). On each plate, a column of no enzyme negative controls and no inhibitor (DMSO only) positive controls were included. The plate was briefly centrifuged (1000 rpm), then incubated (ambient temperature,15 min). A solution containing 100 mM TAPS (pH 8.5), 50 μM tigecycline and 500 μM NADPH was then dispensed, with 25 μL per well. The plate was briefly centrifuged (1000 rpm), then transferred to a CLARIOstar Plus Microplate reader. The absorbance was monitored at wavelength 400 nM over the course of 2 hours (23 flashes / cycle, 180 s cycle time, 41 data points, 25 ° C). The initial rate was calculated by measuring the rate of change of A_{400} during the initial linear regime. Initial rates were normalised between positive and negative controls. The normalised % response was plotted as a function of $\log_{10}$([Inhibitor]) in molar units, and the IC_{50} was determined as above.

Spectrophotometric Assay for Turnover of Fluorescent Probe 6 by Tet(X4)

A solution containing 100 mM TAPS buffer (pH 8.5), 4 mM glucose-6-phosphate, 4 U/mL glucose-6-phosphate dehydrogenase and 4 mM $NADP^+$ was prepared and pre-incubated at 37 °C for 10 minutes. 5 mM $MgCl_2$, 10 μM FAD and 10 μM Tet(X4) were added, followed by 100 μM compound **6**. The reaction was monitored by recording the absorbance spectra between 200-800 nm at one-minute intervals with 800 rpm stirring and a temperature of 30 °C using a Cary UV-Vis Compact Peltier spectrophotometer (Agilent). For the negative control reaction, the volume of enzyme solution added was replaced by buffer.

LC-MS Assay for Turnover of Fluorescent Probe 6 by Tet(X4)

A 1 mL solution containing 100 mM TAPS buffer (pH 8.5), 10 μM FAD, 5 mM $MgCl_2$ and 100 μM NADPH was prepared. 50 nM Tet(X4) was then added; then reaction was initiated by the addition of

10 μM **6**. The reaction mixture was mixed vigorously then incubated (30 °C, 600 rpm). 100 μL aliquots were taken at specified timepoints and quenched by mixing with 10 μL 10%$_{v/v}$ formic acid solution. Quenched samples were stored at -20 °C prior to analysis. Samples were analysed using a Xevo G2-XS Q-TOF mass spectrometer equipped with an electrospray source (Waters) coupled to a ACQUITY UPLC system equipped with an ACQUITY BEH C18 column (20 x 50 mm, 1.7 μM pore size) (Waters). Instrument control and data processing were performed using MassLynx V4.1 software.

$MgCl_2$ Binding Assays

A two-fold, eleven-point dilution series of $MgCl_2$ (top concentration = 1 M) in purified water solutions were made in a V-bottom 96-well plate (Greiner Bio-One). Solutions containing 80 nM Tet(X4) and 2 μM FAD in buffer containing 100 mM Tris (pH 7.0) with 0.01% Triton X-100 were dispensed into all columns of a 384-well black, non-binding microtiter plate (Greiner Bio-One), 12.5 μL per well, using a microplate reagent dispenser. A 0.5 μL aliquot of each dilution was transferred in quadruplicate to the 384-well plate containing enzyme solution using a CyBi-Well liquid-handling robot (CyBio). A solution of 50 nM **6** in assay buffer was then dispensed, with 12.5 μL per well. The plate was briefly centrifuged (1000 rpm), then incubated at ambient temperature for 30 mins prior to measuring the FP using a PHERAstar FS microplate reader (BMG Labtech) equipped with a FP optic module (λ_{ex} = 540 ± 20 nm, λ_{em} = 590 ± 20 nm, 200 flashes per well). ΔmP values were calculated by subtracting the average mP of the negative control from the mP measured for each well.

FAD Binding Assays

Two-fold, eleven-point dilutions of FAD (top concentration = 0.5 mM) in water were made in a V-bottom 96-well plate (Greiner Bio-One). Solutions containing 80 nM Tet(X4) and 10 mM $MgCl_2$ in buffer containing 100 mM Tris (pH 7.0) with 0.01%$_{v/v}$ Triton X-100 were dispensed into all wells of a 384-well black, non-binding microtiter plate (Greiner Bio-One), 12.5 μL per well, using a microplate reagent dispenser. A 0.5 μL aliquot of each dilution was transferred in quadruplicate to the 384-well plate containing enzyme solution using a CyBi-Well liquid-handling robot (CyBio). A solution of 50 nM **6** in assay buffer was then dispensed, with 12.5 μL per well. The plate was briefly centrifuged (1000 rpm), then incubated at ambient temperature for 30 mins prior to measuring the FP using a PHERAstar

FS microplate reader (BMG Labtech) equipped with a FP optic module (λ_{ex} = 540 ± 20 nm, λ_{em} = 590 ± 20 nm, 200 flashes per well). ΔmP values were calculated by subtracting the average mP of the negative control from the mP measured for each well.

Molecular Docking

All molecular docking simulations were performed using the maximum common substrate docking model[3] implemented in AutoDock Vina (v1.2.0)[4]. A crystal structure of type 1 TDase Tet(X4) in complex with tigecycline (PDB:7EPW, 2.10 Å)[5], retrieved from the Protein Data Bank, was taken as the receptor protein. The crystallographic water and ligand molecules were removed; FAD was retained. Missing residues were added by homology modelling using Modeller[6] via the graphical interface in UCSF Chimera.[7] Protonation and conformational states of titratable residues were assigned at pH 7.4 based on MolProbity,[8] H++[9] and visual inspection: Lys and Arg residues were modelled in their positively charged forms, Asp / Glu residues in their anionic forms; histidine residues were all assigned to be neutral and singly protonated on either the N^d or N^e atoms. The initial structure of ligands was generated using RDKit[10] and hydrogen atoms were added using Dimorphite-DL[11] at pH 7.4. The ligand complex structure was then optimized using the B3LYP/6-31G* level of density functional theory (DFT) in Gaussian 16 package[12]. The receptor protein and ligands were further prepared using AutoDock Tools v4.2.[13] by adding Gasteiger charges and exported in the pdbqt format. The ligand was docked to the receptor protein by generating a receptor grid around the active site taking the centreof crystallographic ligand and applying a cubic box with dimensions 20 × 20 × 20 Å. The obtained results were in the form of ligand-protein binding pose and binding energy values in kcal mol^{-1} which were visualised in PyMOL (Schrödinger, LLC).

Supplementary Figures

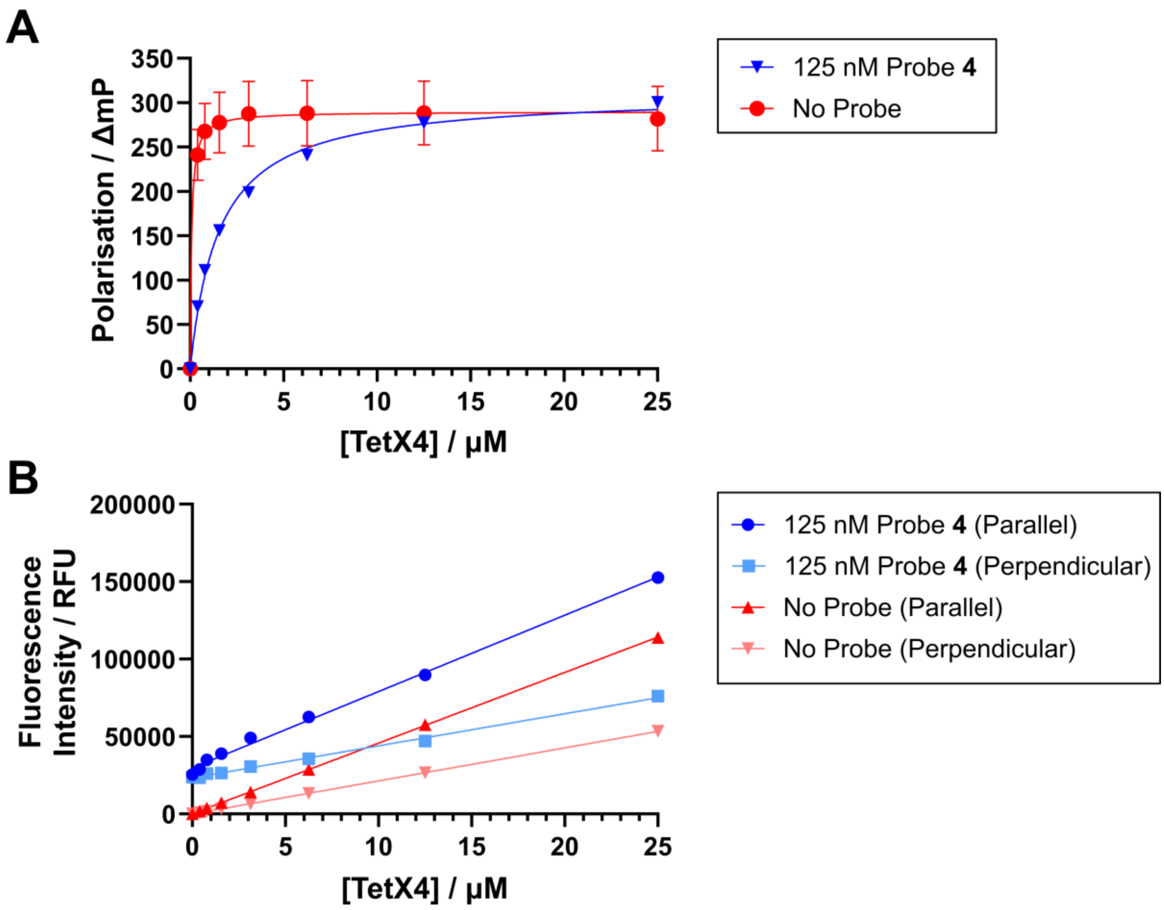

Figure S1 | Fluorescence polarisation binding experiments with the FITC-glycyl-minocycline probe 4 display significant fluorescent background. (A) Dose response curves for Tet(X4) with 125 nM of probe **4** and a control lacking **4**. High polarisation is observed with the Tet(X4) titration series alone, suggesting there is background fluorescence from Tet(X4), likely involving its UV-active FAD cofactor, which is tightly bound to and co-purifies with Tet(X4). (B) Raw fluorescence values for the parallel and perpendicular planes used to calculate polarisation in (A). The background measurement for Tet(X4) contributes substantially to the fluorescence measurement in both the parallel and perpendicular planes, suggesting it influences the polarisation response.

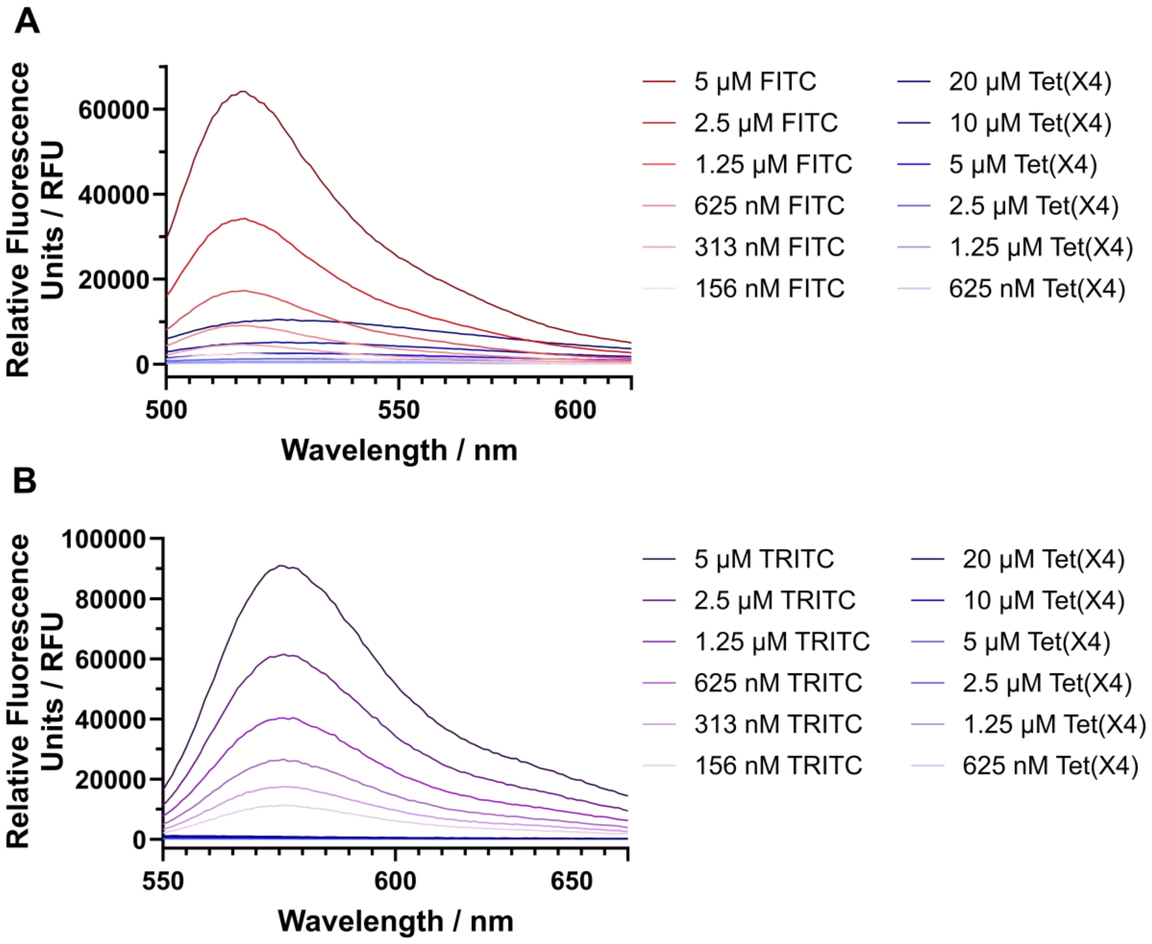

Figure S2 | Fluorescence emission spectra of fluorescent probes versus fluorescence from Tet(X)-bound FAD. (A) Fluorescence emission spectra of a dilution series of FITC-glycyl-minocycline probe **4** plotted versus Tet(X4) concentration. Excitation wavelength = 472 ± 16 nm; emission was measured with a 10 nm bandwidth. (B) Fluorescence emission spectra of a dilution series of TRITC-glycyl-minocycline probe **6** dilution series plotted versus Tet(X4). Excitation wavelength = 522 ± 16 nm; emission was measured with a 10 nm bandwidth. Tet(X4) displayed a significant degree of fluorescent emission at the excitation wavelength of probe **4**, likely from the UV-active FAD co-factor, which caused interference in the FP assay. At the longer excitation-emission wavelengths of **6**, no significant fluorescence was observed with Tet(X4).

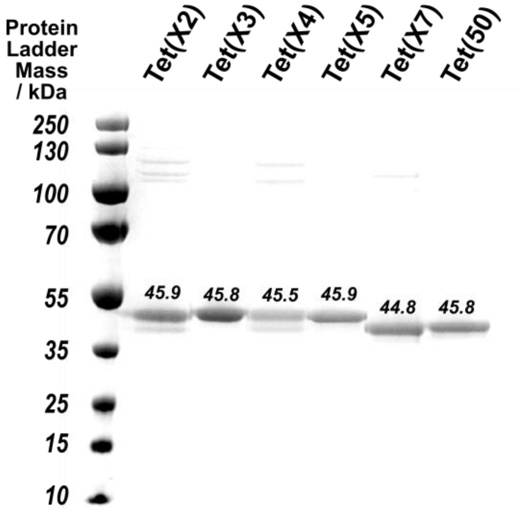

Figure S3 | SDS-PAGE gel analysis of the tetracycline destructases used in this study. Expected masses based on the protein sequence are given above the band of interest (in kDa). Approximately 2 µg of protein was run in each lane. The PageRuler™ Plus Prestained Protein Ladder (Thermo Fisher) was used. Proteins were separated on a 4-12% Novex™ Tris-glycine gel (Thermo Fisher) according to the manufacturer's protocol. Note, apparent minor impurities, not separated during gel filtration, for some Tet(X) enzymes are present between 100-130 kDa.

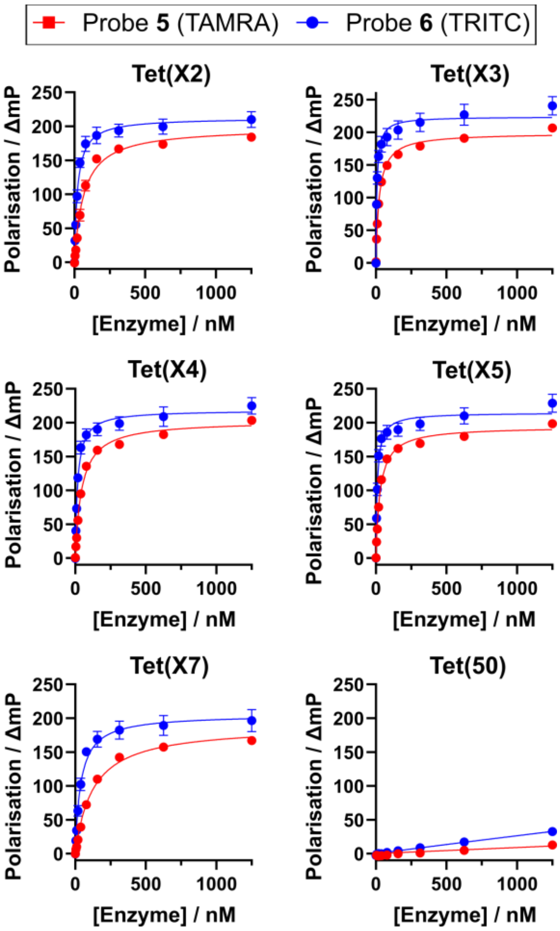

Figure S4 | Fluorescence polarisation measurements allow quantification of binding of tetramethylrhodamine-based fluorescent probes 5 and 6 bind to Tet(X) enzymes with low nanomolar K_D values. Dose-response curves for tetracycline destructases with 25 nM fluorescent probes **5** (red) and **6** (blue), each with 1 µM FAD and 5 mM $MgCl_2$. Both probes show binding with low nM K_D values to the five Tet(X)s tested; no substantial binding was detected with Tet(50). K_D measurements were performed by titrating dilutions of enzyme in buffer containing 100 mM Tris (pH 7.0) and 0.01 % Triton X-100 with 25 nM **5** or **6**, 5 mM $MgCl_2$ and 1 µM FAD. Fluorescence polarisation was measured at $\lambda_{ex} = 540 \pm 20$ nM, $\lambda_{em} = 590 \pm 20$ nm.

	Tet(X2)	Tet(X3)	Tet(X4)	Tet(X5)	Tet(X7)	Tet(50)
Tet(X2)	100					
Tet(X3)	85.82	100				
Tet(X4)	95.06	85.19	100			
Tet(X5)	89.95	85.05	91.17	100		
Tet(X7)	85.71	80.69	88.89	93.39	100	
Tet(50)	23.6	23.31	23.31	23.03	23.58	100

100%

0%

Figure S5 | Identity matrix of amino acid sequences of Tet(X) enzymes used in this study. Percent identities were calculated by performing multiple sequence alignment in the EMBL-EBI Job Dispatcher[14] webservice using MUSCLE[15] with default parameters.

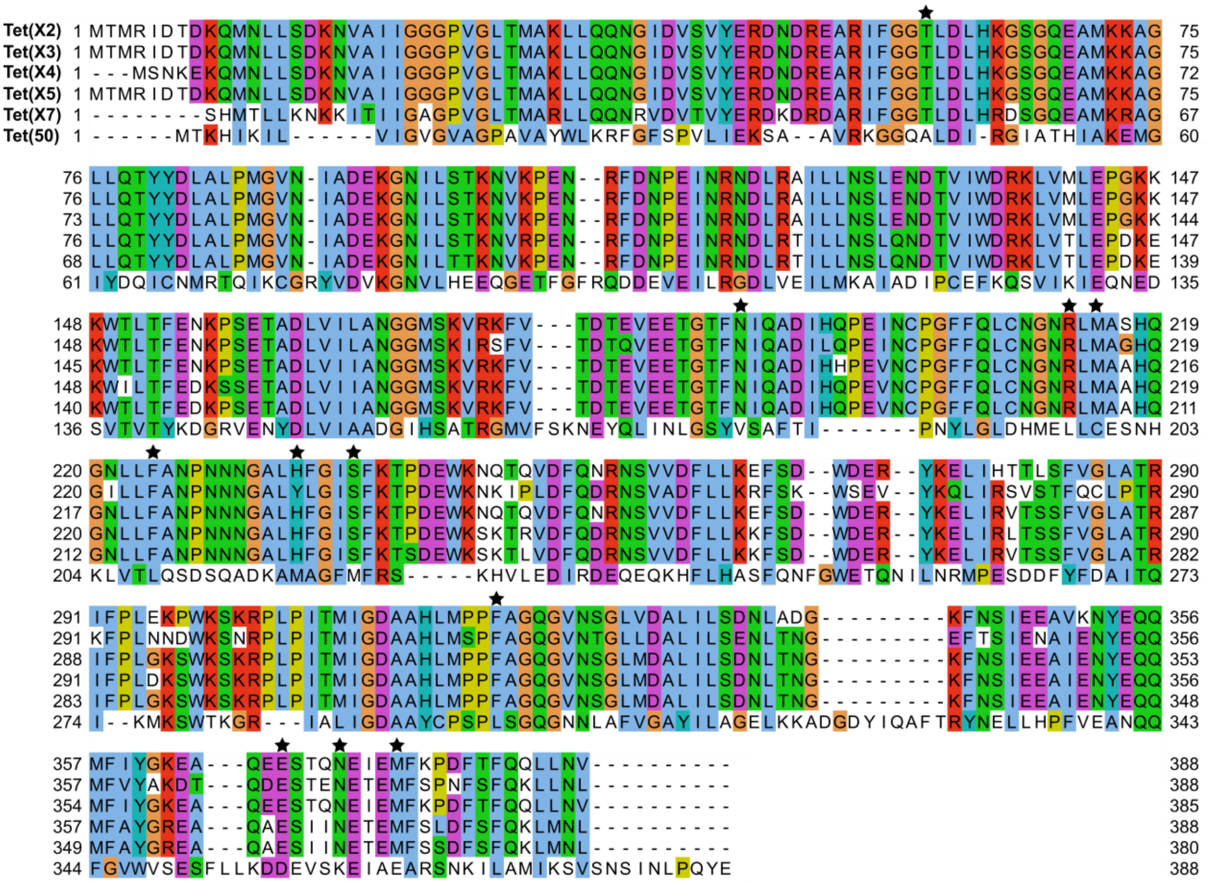

Figure S6 | Sequence alignment of Tet(X) enzymes used in this study. The *N*-terminal His_6-tag sequence derived from the pET-28b vector is not shown. Sequence numbering is provided relative to the *N*-terminal Met of each protein. Residues in the active site, as identified in the crystal structures seen in Figure 2a, are indicated with a star (★). Complete conservation of these residues is observed across the Tet(X) enzymes tested. Multiple sequence alignment was performed and visualised in Jalview v2.11.3.3[16] using MUSCLE[15] with default parameters.

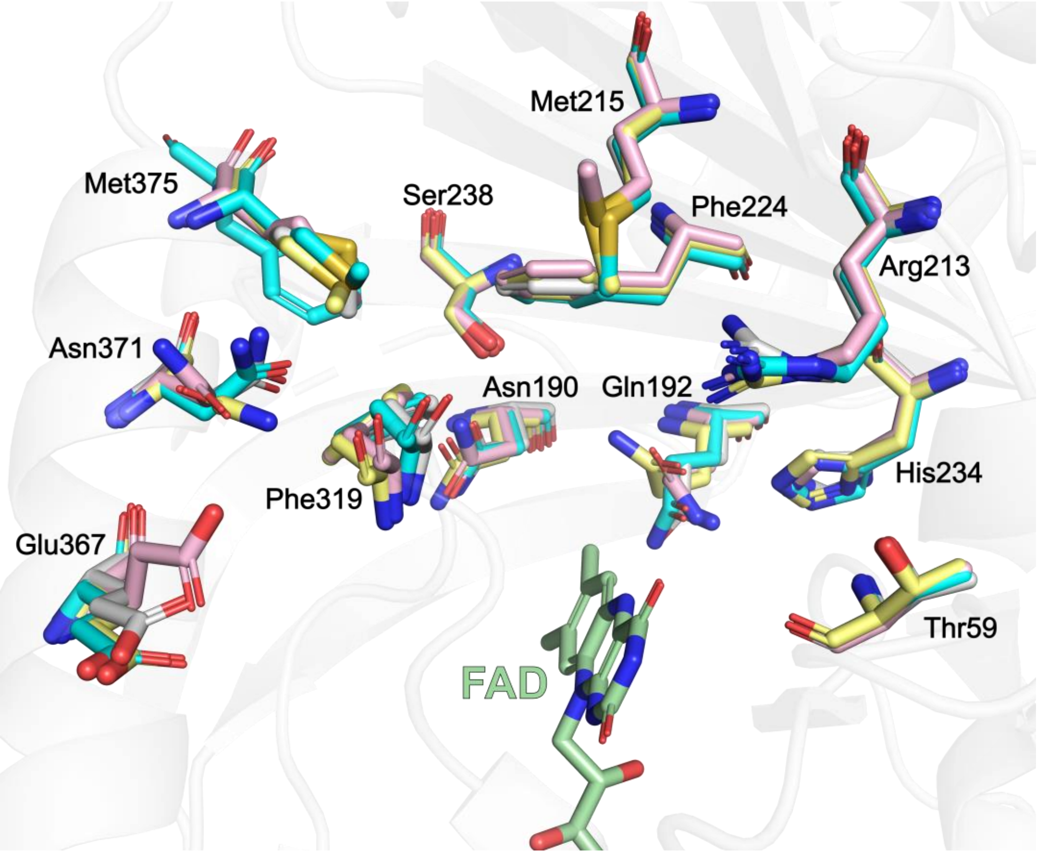

Figure S7 | Comparison of the active site architecture observed in reported Tet(X) structures. The included structures are: Tet(X2) with tigecycline (PDB: 4A6N, *pink*); Tet(X4) (PDB: 7EPV, *grey*); Tet(X6) with anhydrotetracycline (PDB accession: 8ER0, *yellow*); and Tet(X7) (PDB accession: 6WG9, *cyan*). Ligands have been omitted for clarity. All active sites possess identical residues with highly similar conformations. Superimposition of structures was performed using PyMOL v2.5.0 (Schrödinger, LLC).

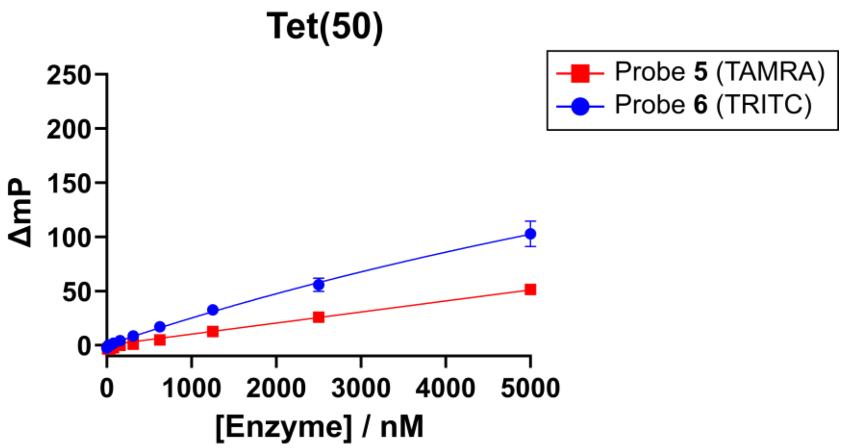

Figure S8 | Extended dose-response curves up to a highest enzyme concentration of 5 µM for the interaction of Tet(50) with fluorescent probes 5 (red) and 6 (blue). Assays contained 100 mM Tris (pH 7.0) with 0.01% Triton X-100, 25 nM of probe **5** or **6**, 1 µM FAD and 5 mM $MgCl_2$. All experiments contained 4 technical replicates for each datapoint in the assay plate. Error bars represent standard deviations derived from three independent trials; error bars not shown are smaller than the size of the data symbols. Up to a concentration of 5 µM of protein, the response of both probes is not fully saturated, indicating weak binding.

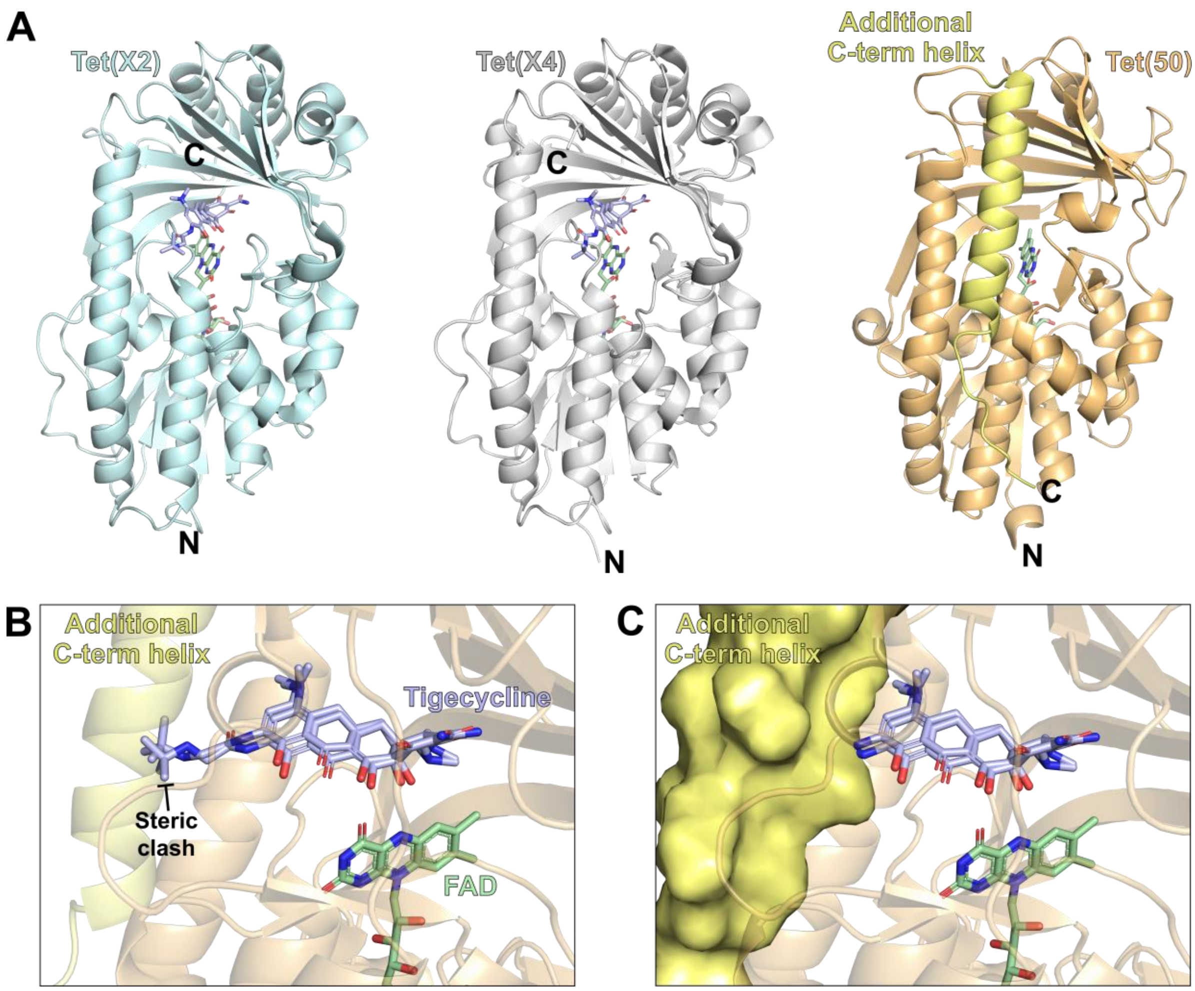

Figure S9 | Structural views of Tet(50) suggest the C-terminal helix may inhibit binding of 9-substituted tetracyclines. (A) Global surface and cartoon representations of: the tigecycline-Tet(X2) complex (*left*, grey, PDB: 4A6N)[17]; the tigecycline-Tet(X4) complex (*centre*, grey, PDB: 7EPW)[5]; and apo-Tet(50) (*right*, orange, PDB: 5TUE)[18]. The additional C-terminal portion of Tet(50), for which no homology is found in Tet(X) enzymes, is in yellow. (B) Active site view of apo-Tet(50) structure with tigecycline binding modes of Tet(X2) and Tet(X4) overlaid. (C) As in panel (B), with the additional C-terminal helix of Tet(50) highlighted with a Connolly surface representation.[19] Note that the C9 tert-butylglycylamido group is predicted to clash with the C-terminal helix in Tet(50), which is highlighted in yellow, preventing turnover of tigecycline by Tet(50). **5** and **6**, which have similar bulky groups at the C9 position of their minocycline cores, would likely make similar steric clashes with Tet(50), preventing binding. Superimposition of structures was performed using PyMOL v2.5.0 (Schrödinger, LLC).

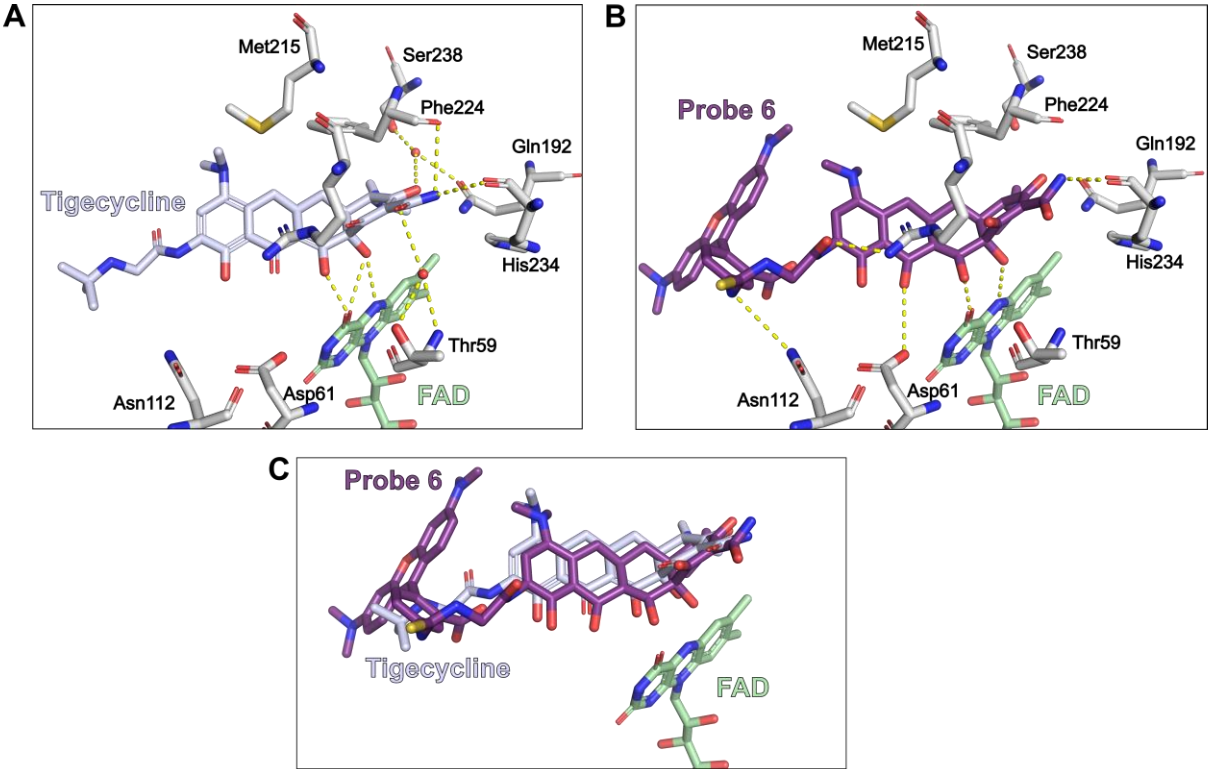

Figure S10 | Comparison of the docking pose of probe 6 with the binding mode of tigecycline in the Tet(X4) active site. (A) Active site view of the tigecycline-Tet(X4) complex (PDB: 7EPW).[5] (B) Docked complex of **6** with Tet(X4). Note that water molecules were removed from the protein during docking. The binding energy value calculated was -7.1 kcal mol^{-1}. (C) Overlay comparing the binding conformation of tigecycline with the docking pose of **6**. See Materials and Methods for detailed docking methodology.; the literature tigecycline-Tet(X4) complex was used as the receptor. Docking was performed in AutoDock Vina (v.1.2.0).[4] Visualisation and superimposition of structures was performed using PyMOL v2.5.0 (Schrödinger, LLC).

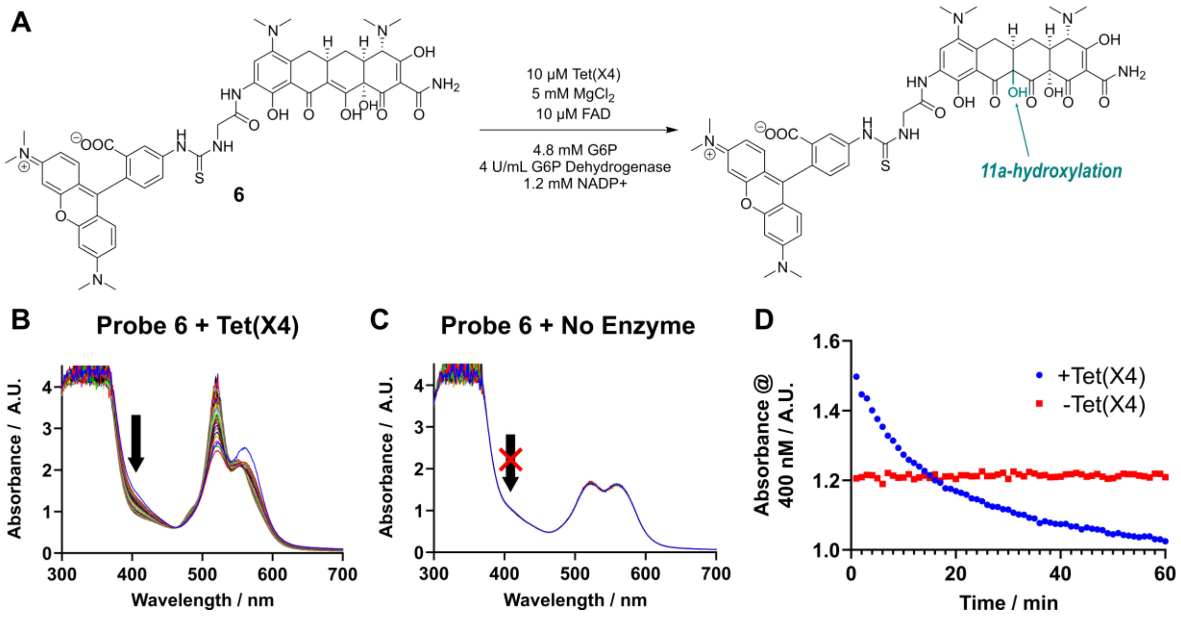

Figure S11 | Turnover of probe 6 by Tet(X4) as monitored by an absorbance-based assay. (A) Conversion of **6** to the proposed 11a-hydroxylated product. (B) Absorbance spectra as a function of time for the Tet(X4)-catalysed degradation of **6**. A time-dependent decrease in absorbance of the shoulder peak at 400 nm corresponding to the conjugated keto-enol form of the minocycline core is observed, indicating of turnover. (C) Absorbance spectra taken as a function of time for a no enzyme control of the same reaction. No change in absorbance for the shoulder peak at 400 nm is observed, indicating turnover is enzyme catalysed. (D) Plots of absorbance at 400 nM over time, derived from spectra shown in B and C.

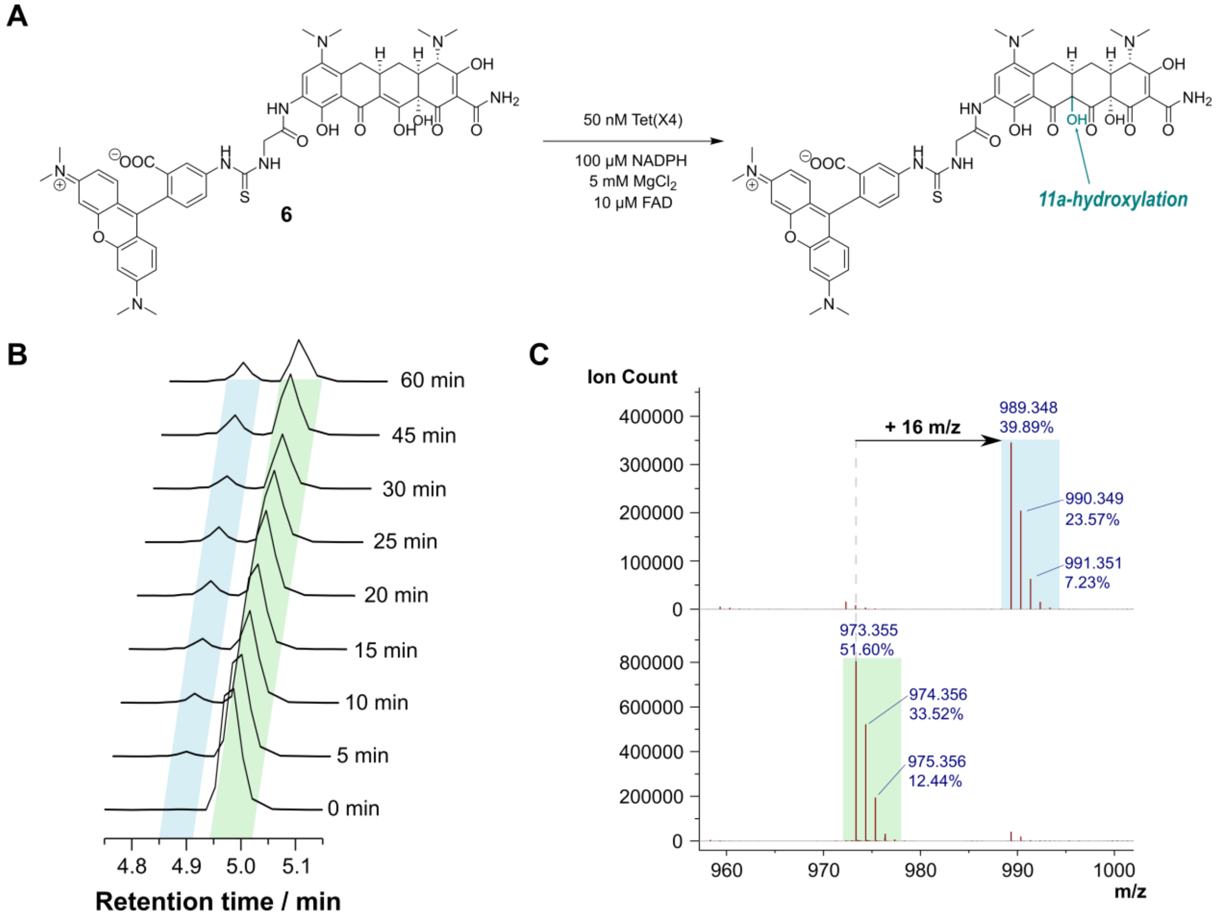

Figure S12 | Turnover of probe 6 by Tet(X4)as monitored by LC-MS. (A) Scheme showing turnover of **6**, with the site of hydroxylation predicted by analogy to reported results of Tet(X) reactions.[20,21] (B) Extracted ion chromatograms (EICs) showing depletion of the starting material peak and appearance of a peak corresponding to the anticipated product. The reaction was quenched by the addition of $1\%_{v/v}$ formic acid solution at the given timepoints. EICs in all cases are the sum of the 973.356, 487.181, 989.350, 495.179 ± 20 ppm mass peak areas, representing the $[M+H]^+$ and $[M+2H]^{2+}$ peaks for the starting material and product. (C) Mass spectrum of probe **6** and the proposed 11a-hydroxylated product (expected masses of 973.356 Da and 989.350 Da, respectively, giving mass deviations of 0.5 and 2.4 ppm from expected). Together with the absorbance assay results (Figure S11), these data imply turnover of **6** is catalysed by Tet(X4), suggesting it binds to the active site in a similar manner to studied tetracyclines antibiotics.

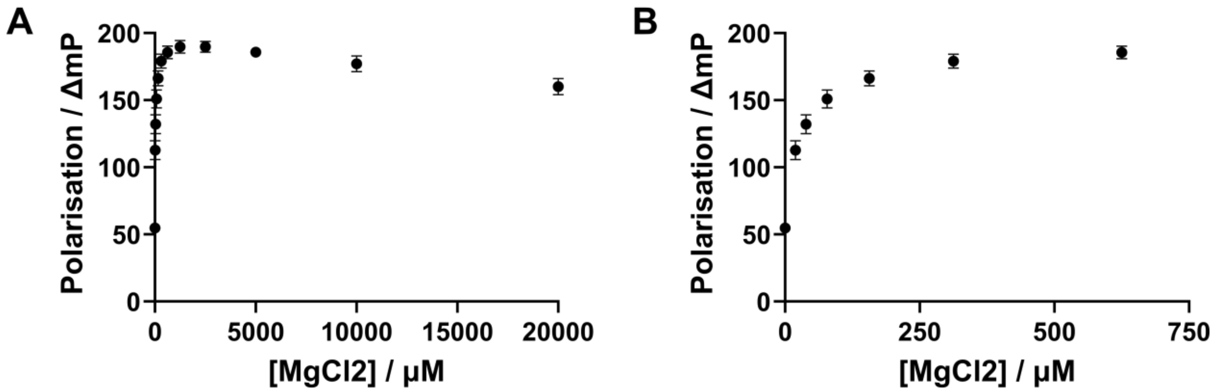

Figure S13 | Addition of $MgCl_2$ to the fluorescence polarisation assay improves binding of probe 6 to Tet(X4). (A) Dose-response of the polarisation reading with respect to $MgCl_2$ concentration. (B) As in (A), with the x-axis trimmed to highlight the lowest concentrations of $MgCl_2$. Experiments contained 4 technical replicates for each datapoint in the assay plate, and error bars represent standard deviations derived from three independent assays.

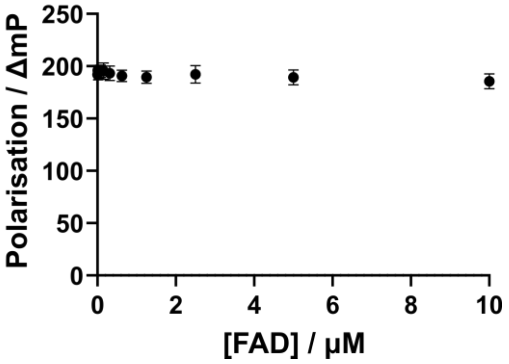

Figure S14 | Dose-response of the polarisation reading with respect to FAD concentration. The results reveal that the presence of excess FAD, in addition to that co-purifying with the protein, does not significantly affect the polarisation response. Conditions: 25 nM **6**, 40 nM Tet(X4) and 5 mM $MgCl_2$ in 100 mM Tris (pH 7.0) with $0.01\%_{v/v}$ Triton X-100. Each experiment contained 4 technical replicates for each datapoint in the assay plate, and error bars represent standard deviations derived from three independent assays.

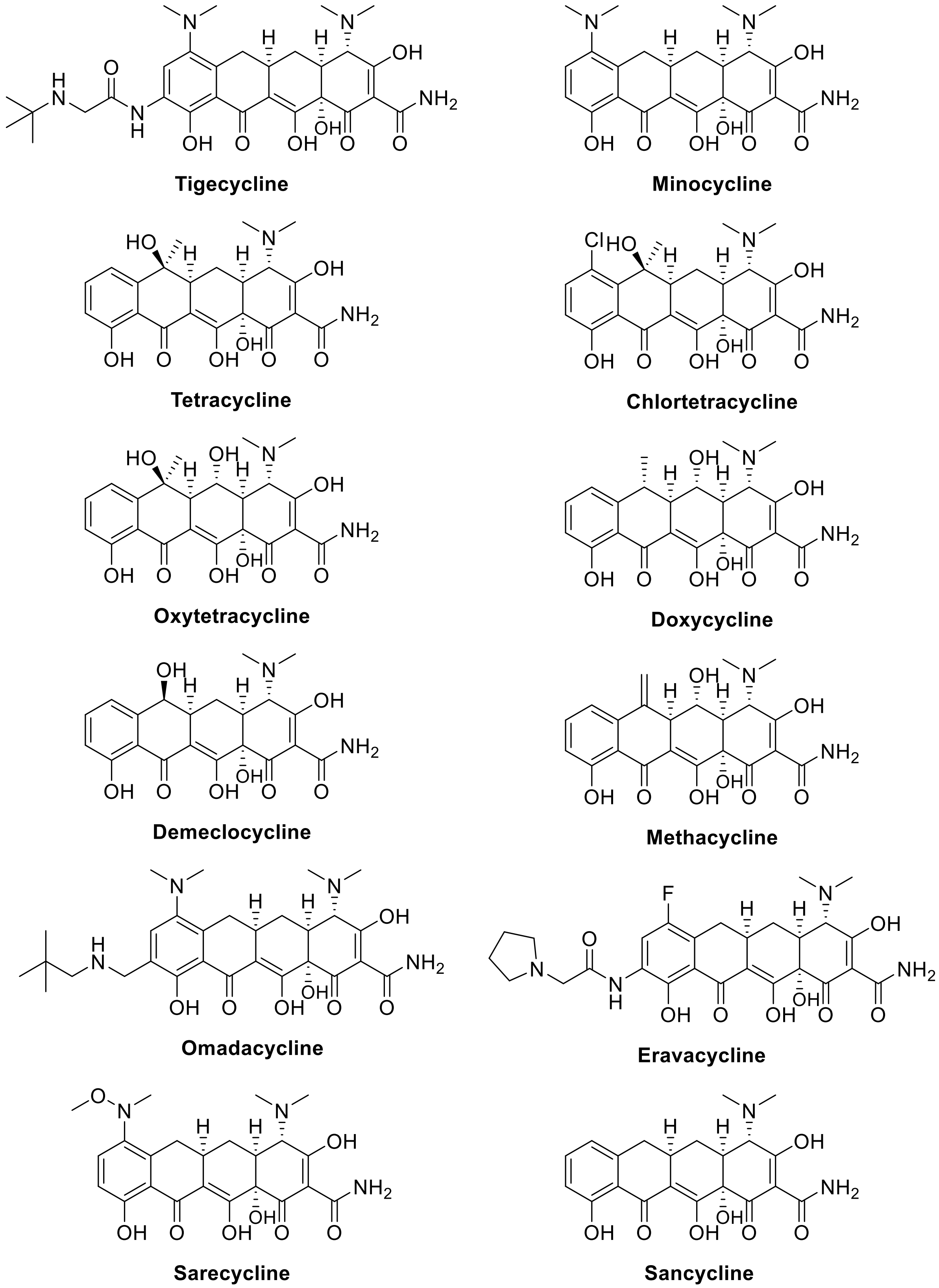

Figure S15 | Structures of the tetracycline antibiotic substrates tested in this study.

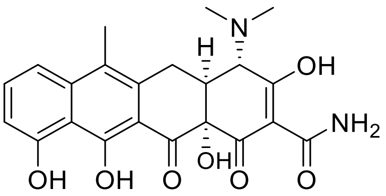

Anhydrotetracycline (aTC)

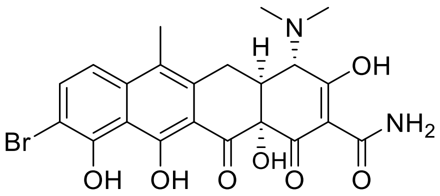

9-Bromo-anhydrotetracycline (9-Br-aTC)

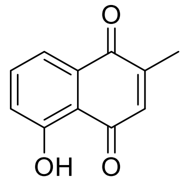

Plumbagin

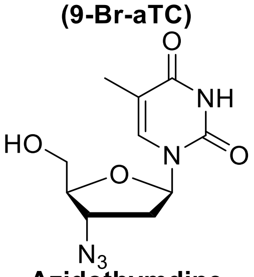

Azidothymdine (AZT)

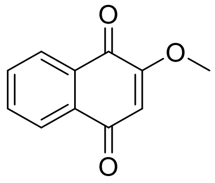

2-Methoxy-1,4-naphthoquinone (2-MNQ)

Figure S16 | Structures of reported Tet(X) inhibitors tested in this study.[2,22–24]

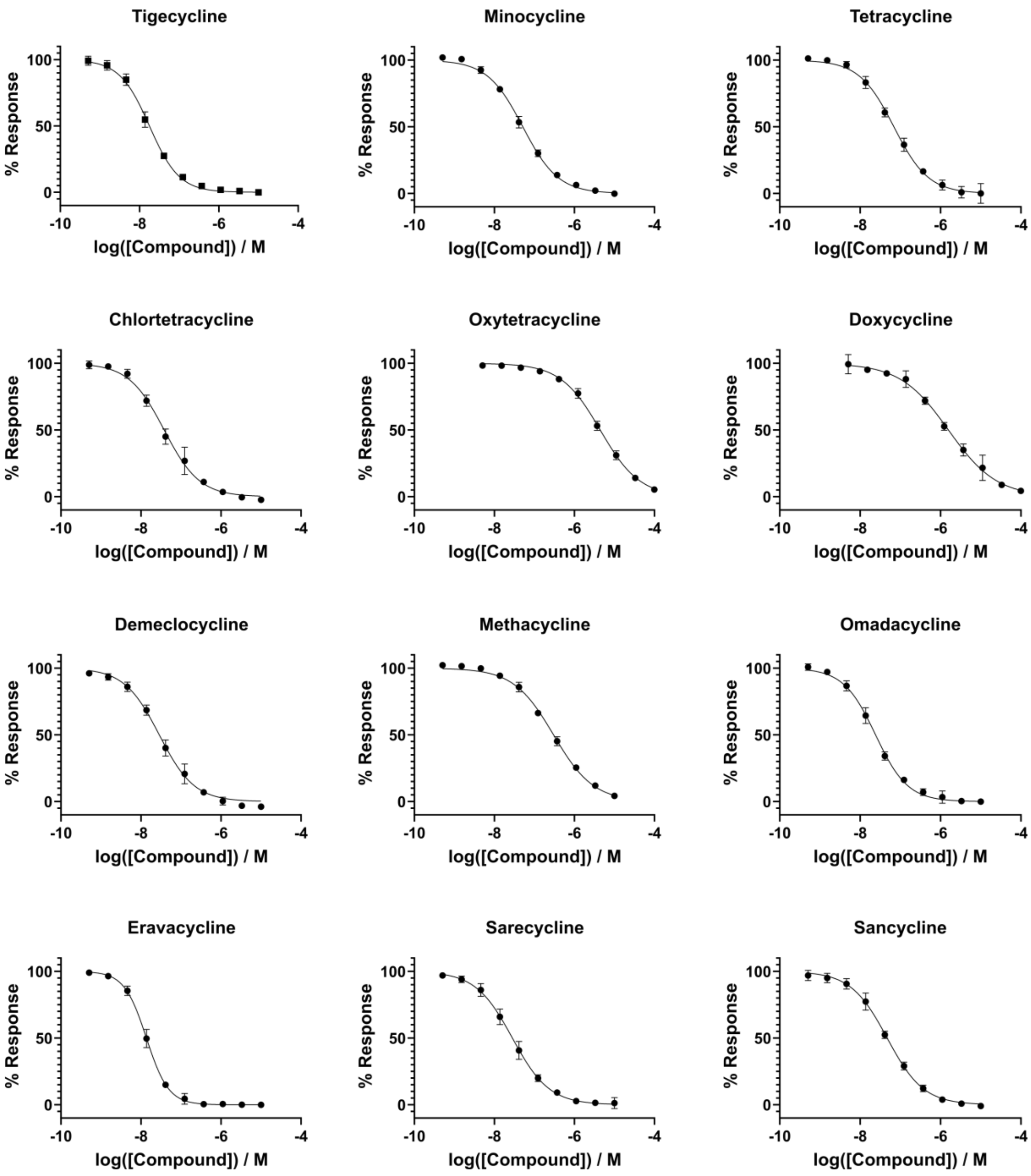
Tigecycline
Minocycline
Tetracycline
Chlortetracycline
Oxytetracycline
Doxycycline
Demeclocycline
Methacycline
Omadacycline
Eravacycline
Sarecycline
Sancycline
% Response
log([Compound]) / M
100
50
0
-10
-8
-6
-4

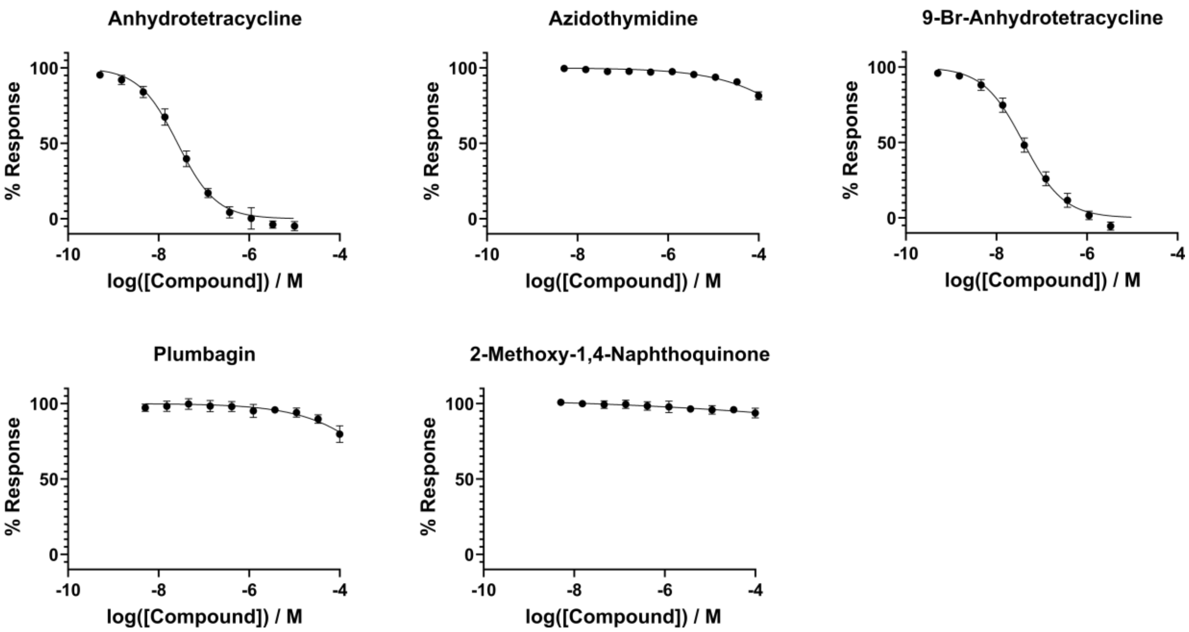

Figure S17 | Dose-response curves for the competitive binding of selected tetracyclines and previously identified Tet(X) inhibitors with probe 6 and Tet(X4). In all experiments, the conditions were: 25 nM **6**, 40 nM Tet(X4), 1 µM FAD and 5 mM $MgCl_2$ in buffer containing 100 mM Tris (pH 7.0) and 0.01%$_{v/v}$ Triton X-100. All experiments were conducted in triplicate on separate days with freshly prepared reagent solutions, with technical quadruplicates in each experiment. Error bars represent the standard deviation for 12 measurements.

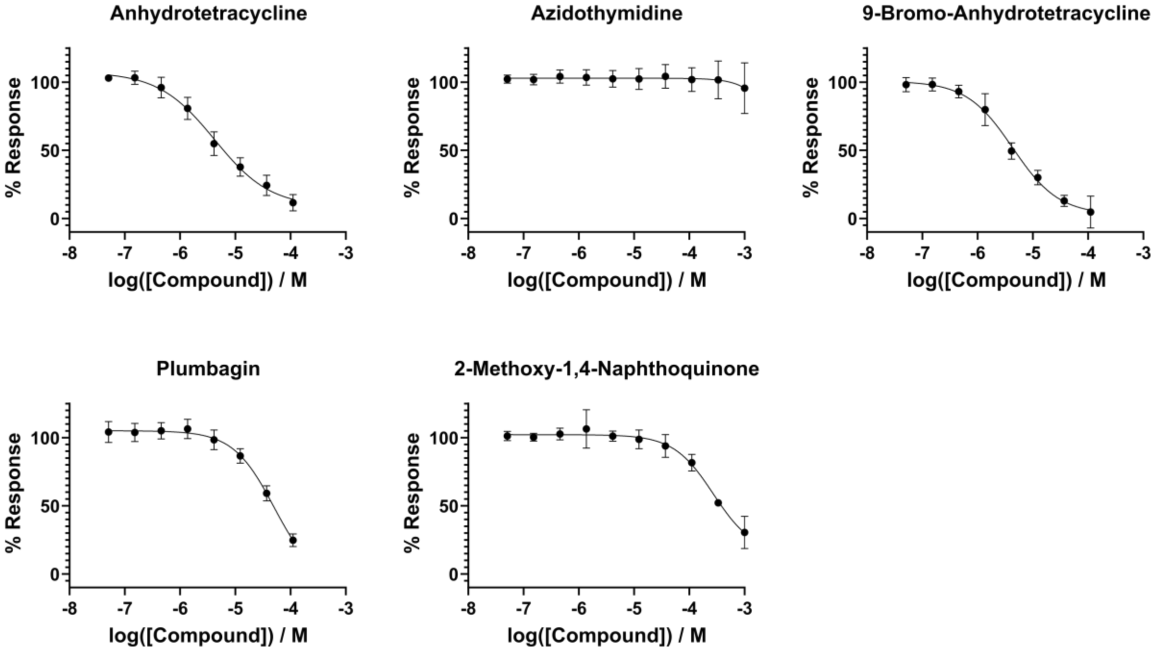

Figure S18 | Dose-response curves for the inhibition of tigecycline turnover to 11a-hydroxytigecycline by Tet(X4) by selected potential inhibitors, monitored by change in absorbance.[2,22–24] In all experiments, conditions were: 25 μM tigecycline, 50 nM Tet(X4), 10 μM FAD, 5mM $MgCl_2$ and 250 μM NADPH in 100 mM TAPS buffer (pH 8.5) with a final concentration of $2\%_{v/v}$ DMSO (100 μL per well in a 384-well clear plate). All assays were conducted in triplicate on separate days with freshly prepared reagent solutions, with technical quadruplicates in each experiment. Error bars represent the standard deviation for 12 measurements.

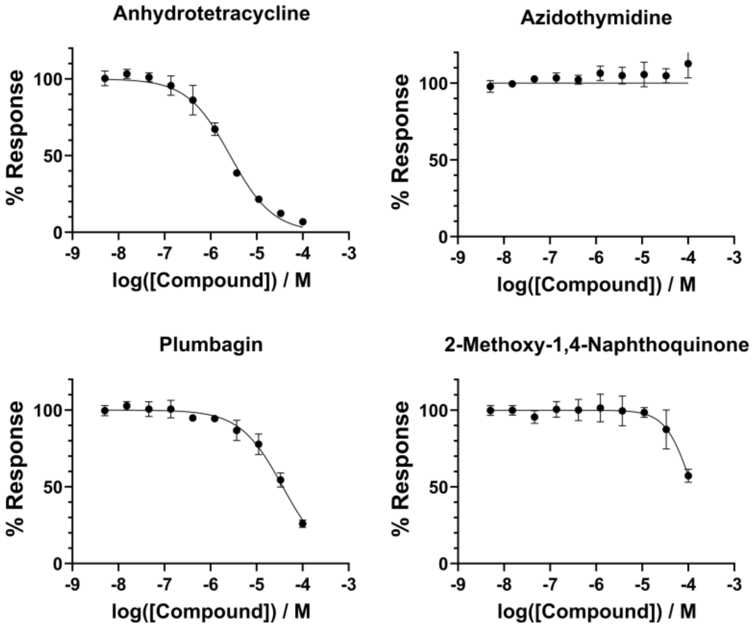

Figure S19 | Dose-response curves for inhibition of tigecycline turnover to 11a-hydroxytigecycline by Tet(X4) by selected potential inhibitors, monitored by UPLC. The integrals of peaks at 254 nm from UPLC analysis were measured to calculate turnover. In all experiments, conditions were: 20 μM tigecycline, 50 nM Tet(X4), 10 μM FAD, 5mM $MgCl_2$ and 100 μM NADPH in 100 mM TAPS buffer (pH 8.5) with a final concentration of 1% DMSO (100 μL per well in a 96-well plate). Reactions were initiated by simultaneous addition of tigecycline and NADPH to the other reagents. Assay plates were incubated (20 minutes, 30 °C), then quenched by addition of a final concentration of 1%$_{v/v}$ formic acid. Error bars represent the standard deviation for three independent measurements conducted on separate days, except for anhydrotetracycline, for which six independent measurements were taken.

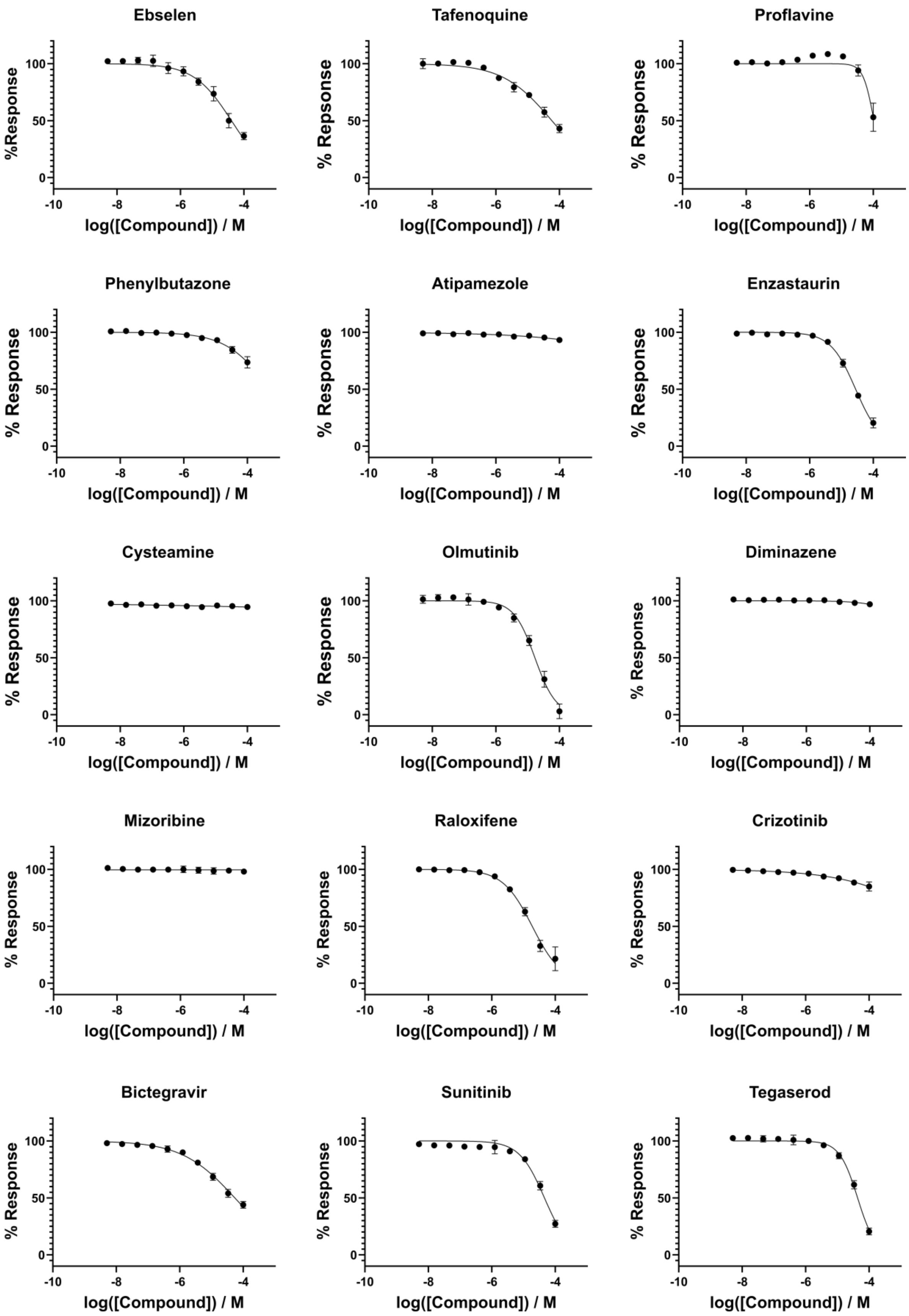

Ebselen
%Response
log([Compound]) / M
Tafenoquine
% Repsonse
log([Compound]) / M
Proflavine
% Response
log([Compound]) / M
Phenylbutazone
% Response
log([Compound]) / M
Atipamezole
% Response
log([Compound]) / M
Enzastaurin
% Response
log([Compound]) / M
Cysteamine
% Response
log([Compound]) / M
Olmutinib
% Response
log([Compound]) / M
Diminazene
% Response
log([Compound]) / M
Mizoribine
% Response
log([Compound]) / M
Raloxifene
% Response
log([Compound]) / M
Crizotinib
% Response
log([Compound]) / M
Bictegravir
% Response
log([Compound]) / M
Sunitinib
% Response
log([Compound]) / M
Tegaserod
% Response
log([Compound]) / M

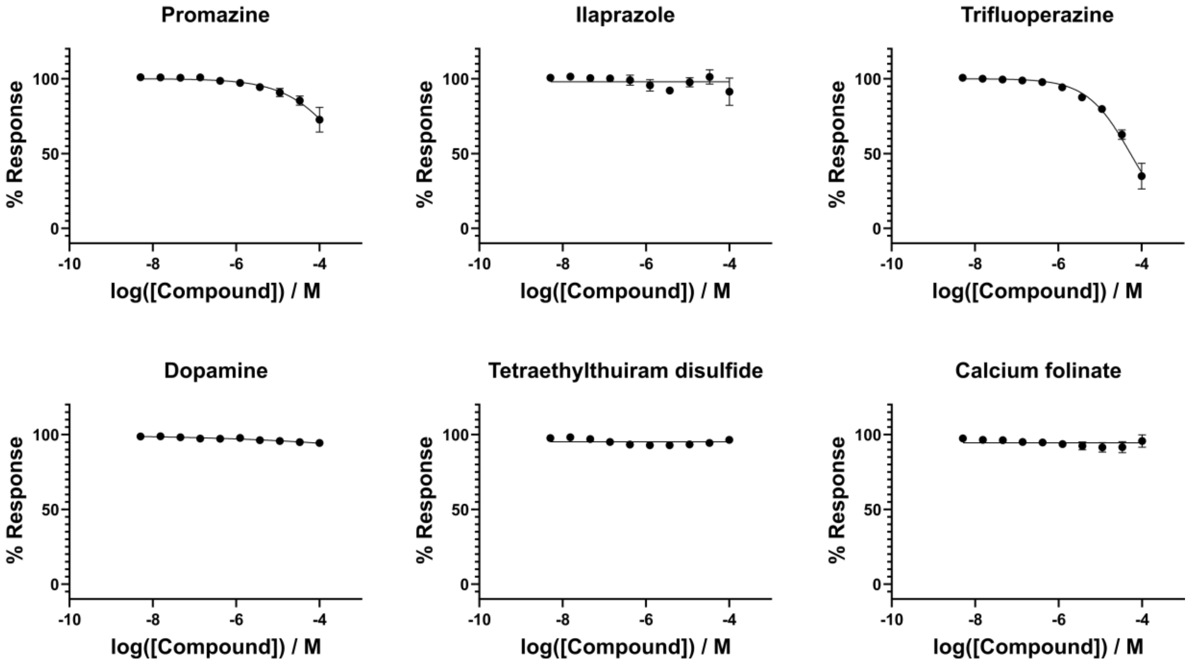

Figure S20 | Hit validation dose-response curves of selected compounds with >15% inhibition in high-throughput screening of the Pharmacopeia drug library, utilising the competitive binding fluorescence polarisation assay with 6 and Tet(X4). In all experiments, conditions were: 25 nM **6**, 40 nM Tet(X4), 1 µM FAD and 5 mM $MgCl_2$ in buffer containing 100 mM Tris (pH 7.0) and 0.01% Triton X-100. All experiments were conducted in triplicate on separate days, with technical quadruplicates in each experiment. Error bars represent the standard deviation for 12 measurements.

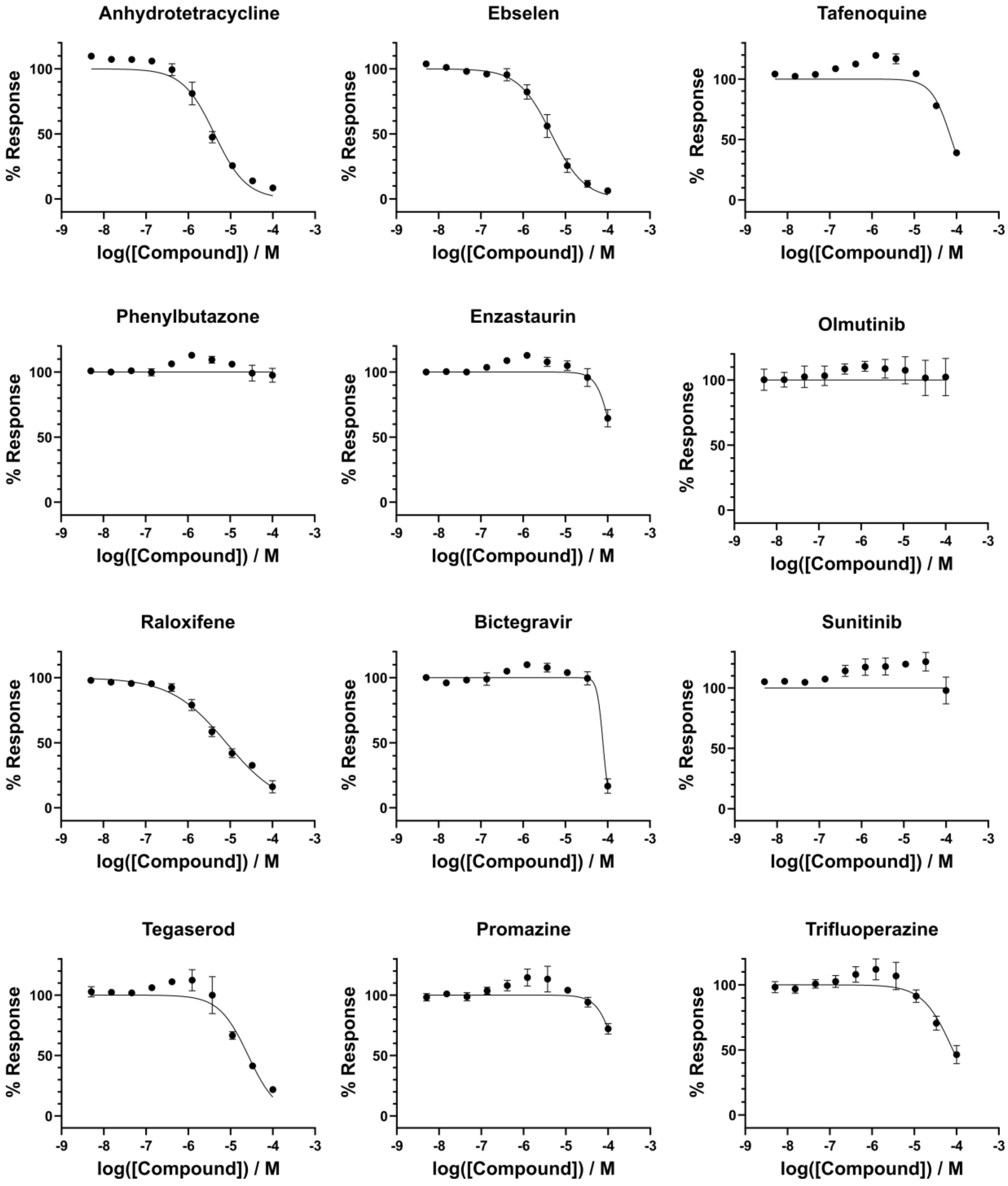

Figure S21 | Hit validation dose-response curves demonstrating selected compounds inhibiting tigecycline turnover to 11a-hydroxytigecycline by Tet(X4), utilising a UPLC -based assay. The integrals of peaks at 254 nm from UPLC analysis were measured to calculate turnover. In all experiments, conditions were: 20 μM tigecycline, 50 nM Tet(X4), 10 μM FAD, 5mM $MgCl_2$ and 100 μM NADPH in 100 mM TAPS buffer (pH 8.5) with a final concentration of 1%$_{v/v}$ DMSO (100 μL per well in a 96-well plate). Reactions were initiated by simultaneous addition of tigecycline and NADPH to the other reagents. Assay plates were incubated (20 minutes, 30 °C), then quenched by addition of a final concentration of 1%$_{v/v}$ formic acid. Error bars represent the standard

deviation for three independent measurements conducted on separate days, except for anhydrotetracycline, for which six independent measurements were taken.

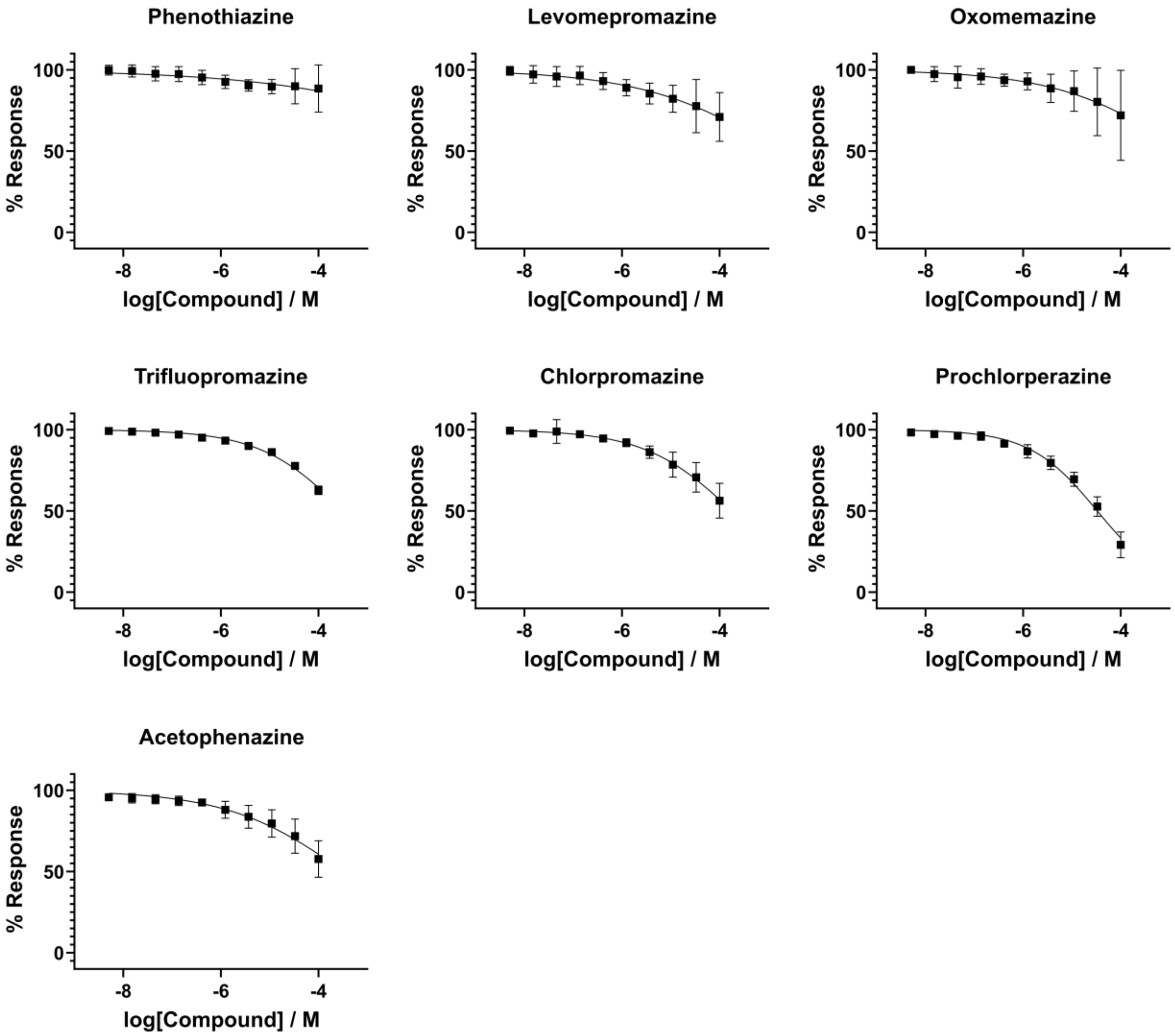

Figure S22 | Dose-response curves demonstrating selected phenothiazine-containing compounds inhibiting tigecycline turnover to 11a-hydroxytigecycline as catalysed by Tet(X4) utilising the competitive binding fluorescence polarisation assay with 6 and Tet(X4). In all experiments, conditions were: 25 nM **6**, 40 nM Tet(X4), 1 µM FAD and 5 mM $MgCl_2$ in buffer containing 100 mM Tris (pH 7.0) and $0.01\%_{v/v}$ Triton X-100. All experiments were conducted in triplicate on separate days, with technical quadruplicates in each experiment. Error bars represent the standard deviation for 12 measurements.

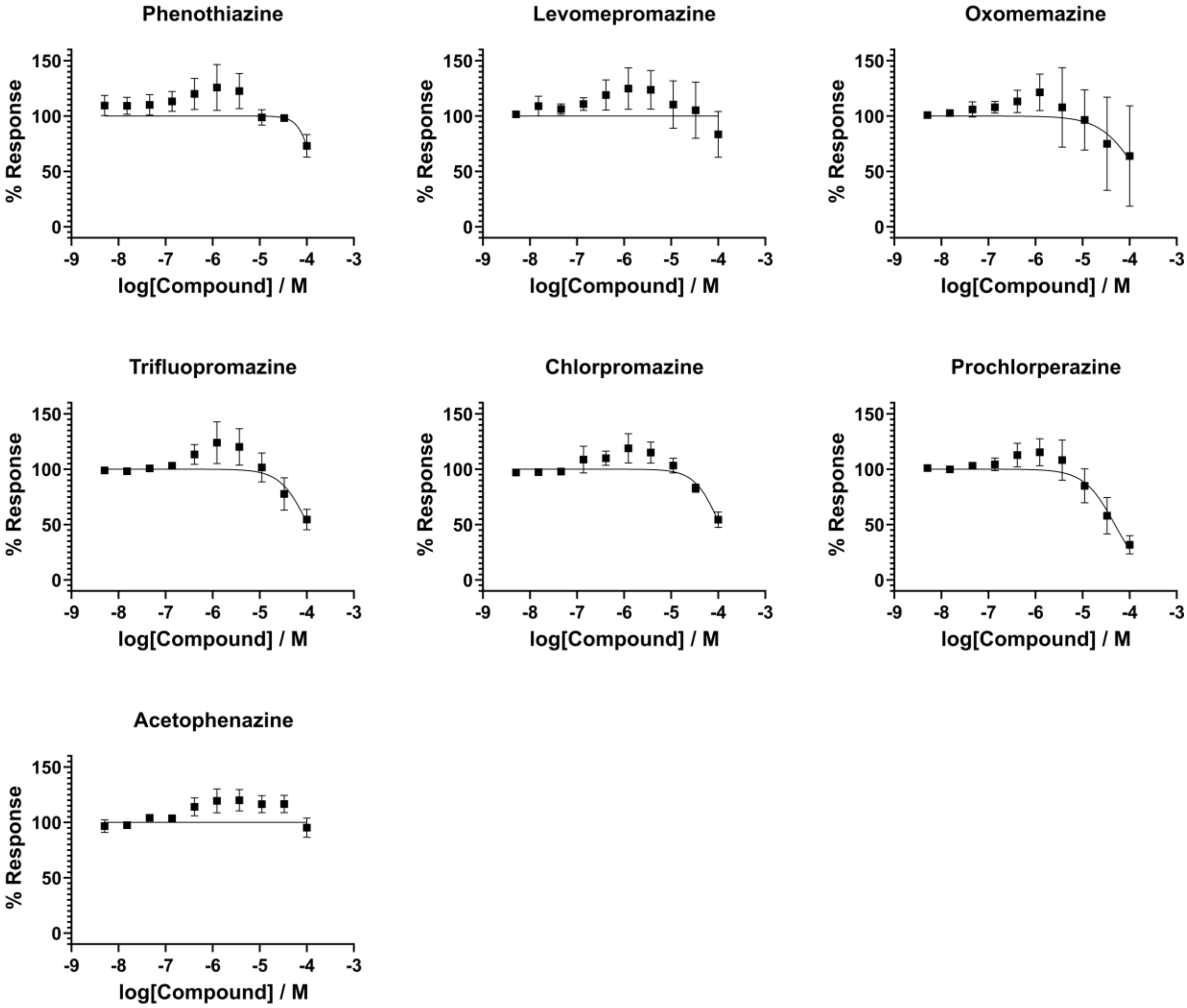

Figure S23 | Dose-response curves demonstrating selected phenothiazine-containing compounds inhibiting tigecycline turnover to 11a-hydroxytigecycline catalysed by Tet(X4), as monitored by measuring peak integrals from UPLC analysis. The integrals of peaks at 254 nm from UPLC analysis were measured to calculate turnover. In all experiments, conditions were: 20 µM tigecycline, 50 nM Tet(X4), 10 µM FAD, 5mM $MgCl_2$ and 100 µM NADPH in 100 mM TAPS buffer (pH 8.5) with a final concentration of 1% DMSO (100 µL per well in a 96-well plate). Reactions were initiated by simultaneous addition of tigecycline and NADPH to the other reagents. Assay plates were incubated (20 minutes, 30 °C), then quenched by addition of a final concentration of $1\%_{v/v}$ formic acid. Error bars represent the standard deviation for three independent measurements conducted on separate days, except for anhydrotetracycline, for which six independent measurements were taken.

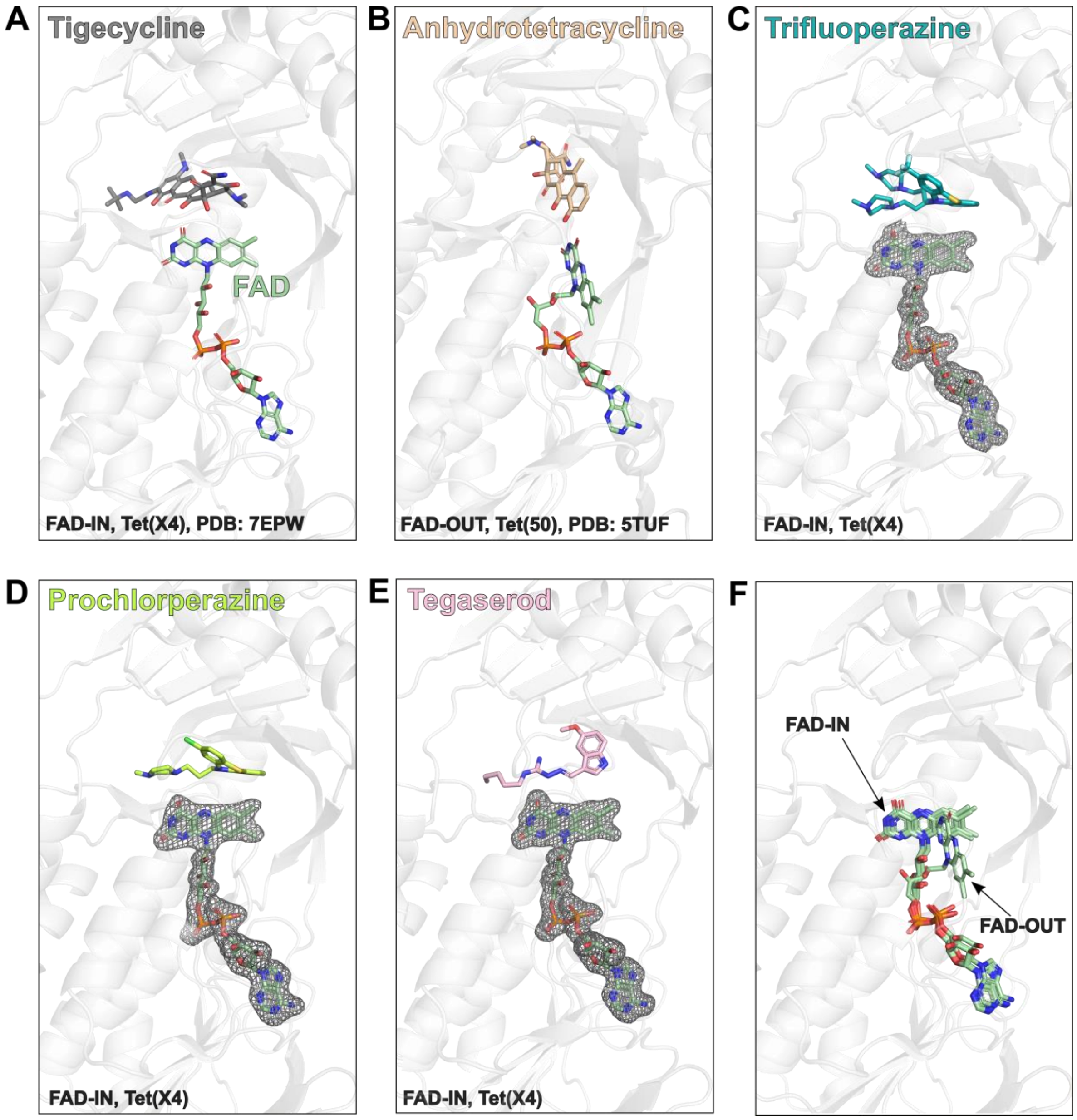

Figure S24 | Complexes of Tet(X4) with trifluoperazine, prochlorperazine and tegaserod contain FAD with the isoalloxazine group directed towards the substrate binding pocket (FAD-IN conformation), which is conserved across type 1 TDase crystal structures.[25] (A) The FAD-IN conformation of the FAD cofactor in the structure of Tet(X4) in complex with tigecycline (PDB: 7EPW).[5] (B) The FAD-OUT conformation of the cofactor in the structure of Tet(50) with the inhibitor anhydrotetracycline (PDB: 5TUF)[18], where the isoalloxazine ring is directed away from the substrate binding pocket and towards the NADPH binding site. This is the only known structure of a tetracycline destructase with the cofactor in the FAD-OUT conformation. (C) The FAD-IN conformation of the FAD cofactor in the structure of Tet(X4) in complex with trifluoperazine. (PDB: 9HKE) (D) FAD-IN conformation of the FAD cofactor in the structure of Tet(X4) in complex with prochlorperazine (PDB: 9HJV). (E) FAD-IN conformation of the FAD cofactor in the structure of Tet(X4) in complex with tegaserod

(PDB: 9HJW). (F) Superimposition of all structures highlighting the change in orientation of the FAD isoalloxazine ring in the FAD-IN and FAD-OUT conformations. Grey mesh in (C)-(E) represents $mF_{obs} - DF_{model}$ polder OMIT maps contoured to 3 σ and carved around the FAD cofactor at 1.8 Å.[26] Superimposition of structures was performed in PyMOL v2.5.0 (Schrödinger, LLC).

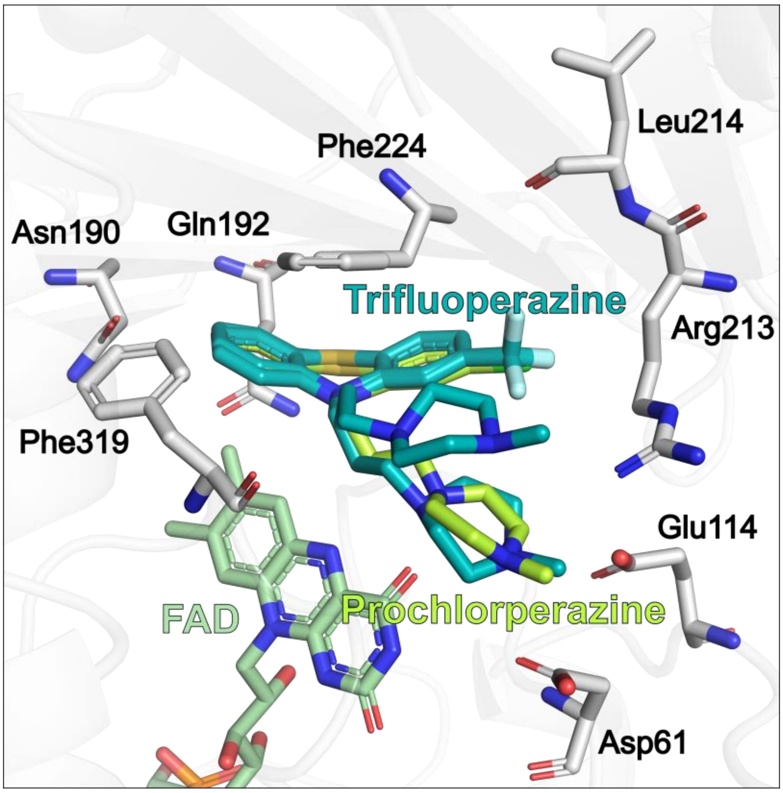

Figure S25 | Comparison of the binding modes of prochlorperazine (PDB: 9HJV) with two potential conformations of trifluoperazine (PDB: 9HKE) in complex with Tet(X4). Superimposition of the structures was performed using PyMOL v2.5.0 (Schrödinger, LLC).

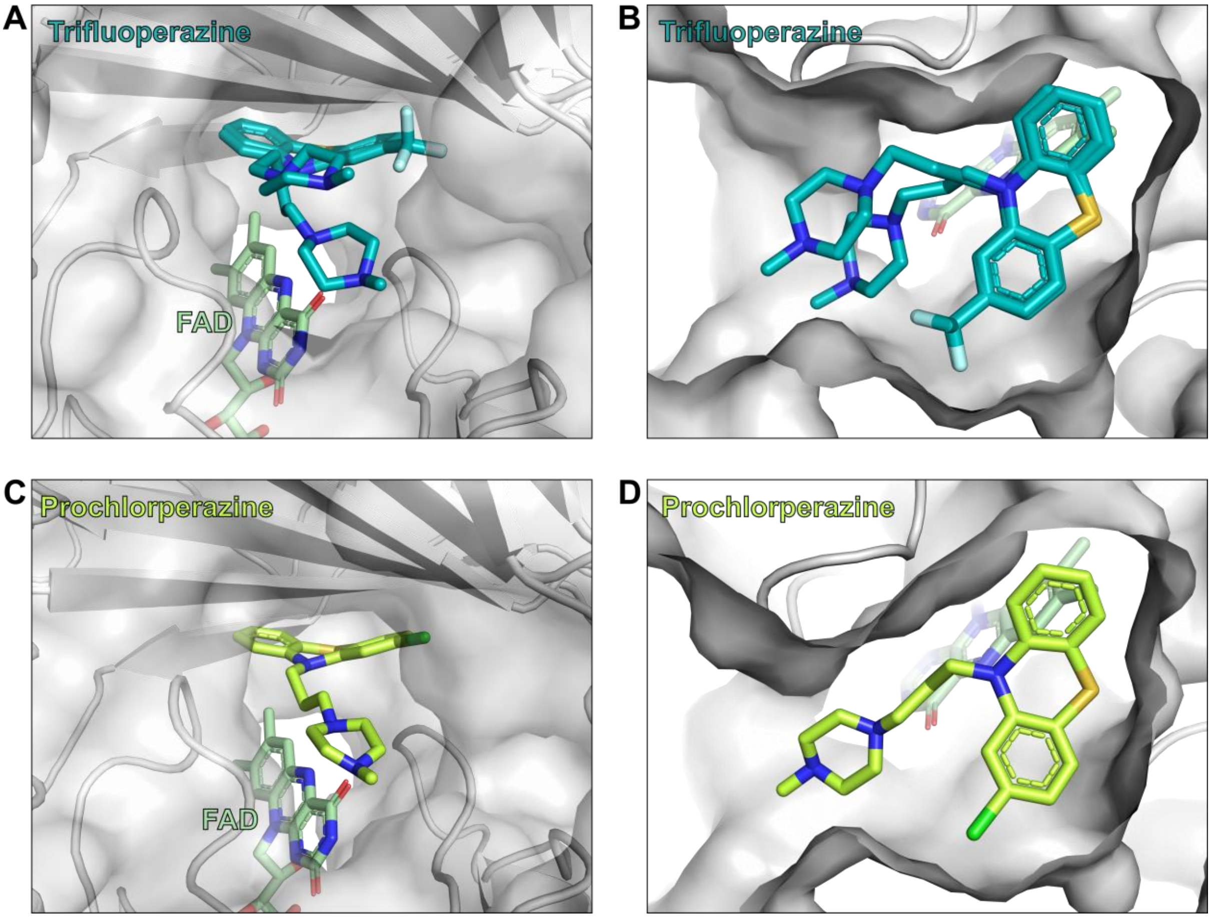

Figure S26 | Phenothiazine derivatives have high shape complementarity for the Tet(X4) active site topology, in particularly around their phenothiazine ring. (A) View of the active site of the trifluoperazine-Tet(X4) complex (PDB: 9HKE). (B) Alternative view of the active site of the trifluoperazine-Tet(X4) complex (PDB: 9HKE). (C) View of the active site of the prochlorperazine-Tet(X4) complex (PDB: 9HJV). (D) Alternative view of the active site of the prochlorperazine-Tet(X4) complex (PDB: 9HJV). Connolly surface representations[19] in all cases were generated by PyMOL v2.5.0 (Schrödinger, LLC).

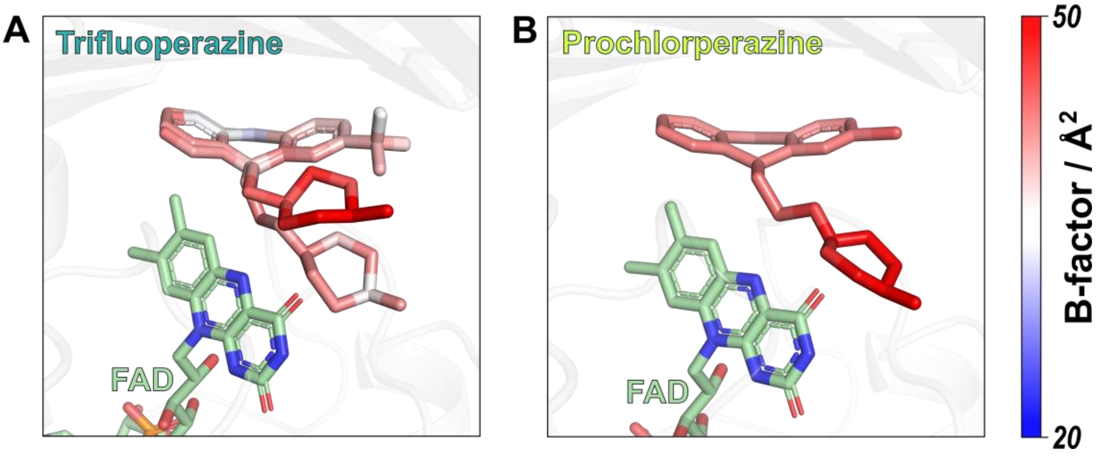

Figure S27 | B-factor analysis of the phenothiazine inhibitors suggests flexibility in the 3-(4-methylpiperazin-1-yl)propan-1-amine side chains of both molecules. (A) B-factor analysis of the two conformations of trifluoperazine in complex with Tet(X4) (PDB: 9HKE). (B) B-factor analysis of the prochlorperazine in complex with Tet(X4) (PDB: 9HJV). B-factors were calculated during structure refinement in Phenix[27] and atoms were coloured by B-factor in PyMOL v2.5.0 (Schrödinger, LLC).

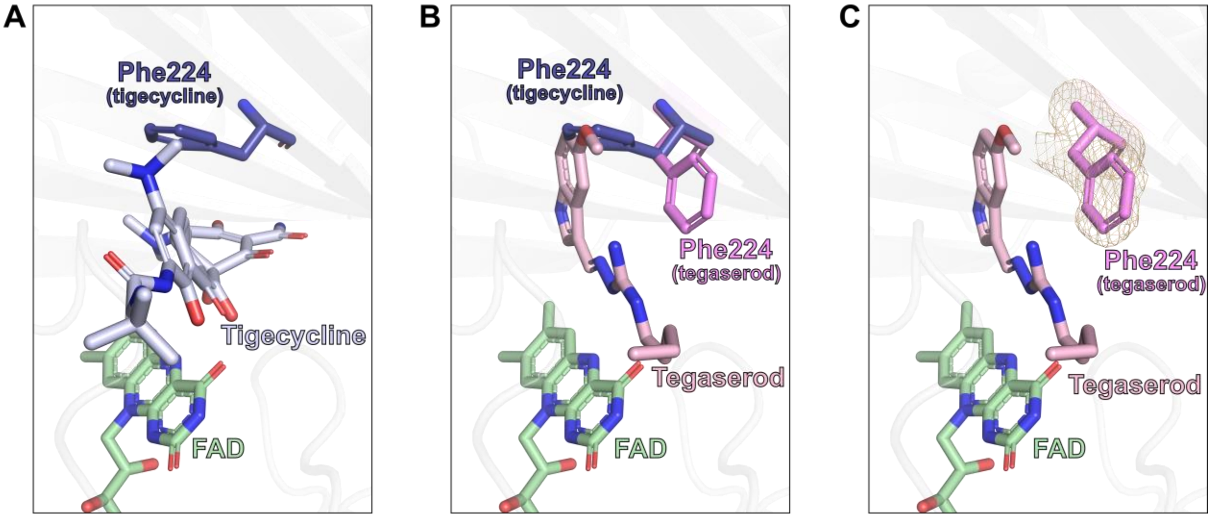

Figure S28 | In the tegaserod-Tet(X4) complex Phe224 is rotated about its α-β bond relative to its position in the tigecycline-Tet(X4) complex to avoid a steric clash with the tegaserod 5-methoxyindole moiety. (A) View of the active site of the tigecycline-Tet(X4) complex (PDB: 7EPW)[5] illustrating the conformation of Phe224. (B) View of the active site of the tegaserod-Tet(X4) (PDB: 9HJW) complex highlighting the rotation of Phe224 compared to that observed when tigecycline is bound. (C) Electron density for Phe224 in the tegaserod-Tet(X4) complex. Orange mesh represents $mF_{obs} - DF_{model}$ polder OMIT maps[26] contoured to 4 σ and carved around the residue at 1.8 Å. The Phe224 conformation in the complex of tigecycline with Tet(X4) is identical to that found in the *apo*-Tet(X4) and phenothiazine-Tet(X4) structures, indicating the conformation observed with tegaserod is unusual.

Table S1 | Half maximal inhibitory concentration (IC_{50}) values measured for hit validation of selected compounds with >15% inhibition from high-throughput screening of the Pharmacopeia drug library. See Materials and Methods section for detailed methods for both assays.

**Compound** (Therapeutic Indication[28] / Bioactivity)	**Structure**	**IC_{50}^{app}, Tet(X4) (Polarization) / µM (n = 3)**	**IC_{50}, Tet(X4) (UPLC) / µM (n = 3)**
Tigecycline	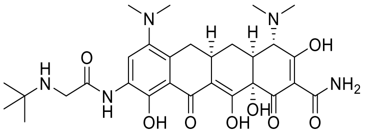	0.022 ± 0.002*	-
Anhydrotetracycline	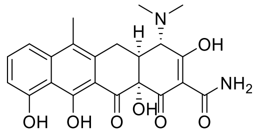	-	4.2 ± 0.7▼
Ebselen (Cysteine-reactive covalent inhibitor)	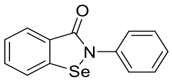	40.9 ± 11.4	4.7 ± 1.5
Tafenoquine (Antimalarial)	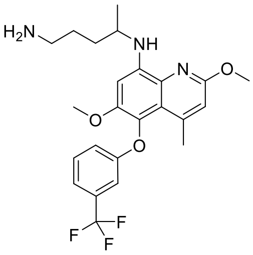	94.9 ± 64.8	76.0 ± 2.4
Proflavine (Antiseptic)	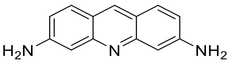	>100	-
Phenylbutazone (Anti-inflammatory)	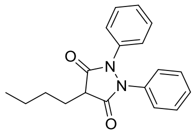	>100	>100
Atipamezole (α_2 adrenoceptor antagonist)	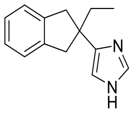	>100	-
Enzastaurin (Glioblastoma multiforme)	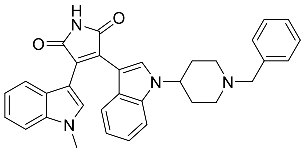	27.8 ± 1.5	>100
Cysteamine (Cystinosis)	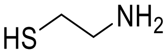	>100	-

Olmutinib<br>(T790M mutation positive non-small cell lung cancer)	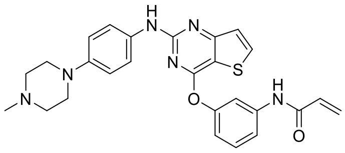	17.2 ± 3.4	>100
Diminazene<br>(Trypanocidal antiprotozoal)	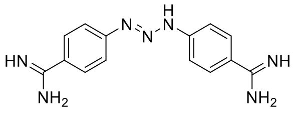	>100	-
Mizoribine<br>(Inosine-5'-monophosphate dehydrogenase 1 inhibitor)	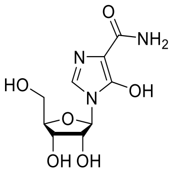	>100	-
Raloxifene<br>(Prvention of postmenopausal osteoporosis and breast cancer)	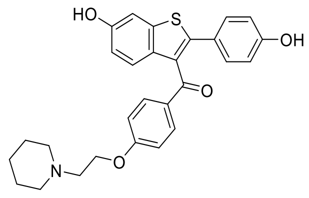	19.8 ± 5.4	8.3 ± 2.1
Crizotinib<br>(Lung cancer, lymphoma)	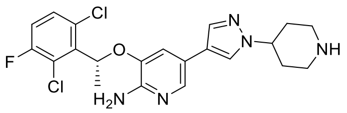	>100	-
Bictegravir<br>(HIV antiviral)	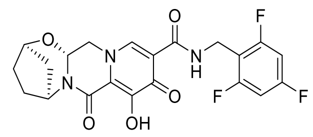	54.8 ± 10.6	76.0 ± 14.0
Sunitinib<br>(Pancreatic/renal/gastrointestinal cancer)	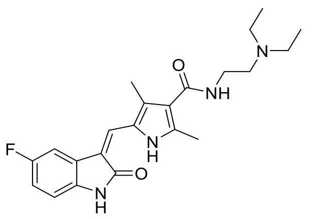	45.6 ± 4.0	>100
Tegaserod<br>(Irritable Bowel Syndrome)	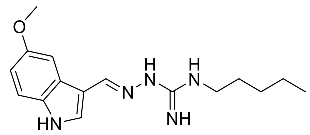	43.2 ± 2.2	25.9 ± 2.4
Promazine<br>(Schizophrenia, Psychosis, Psychomotor agitation)	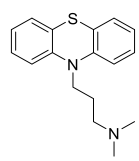	>100	>100
Ilaprazole<br>(Indigestion/Peptic Ulcers)	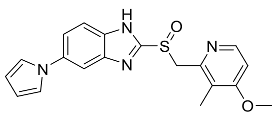	>100	-

Trifluoperazine<br>(Schizophrenia, Psychosis, Psychomotor agitation)	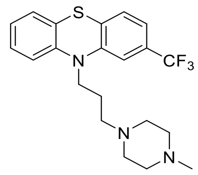	55.6 ± 14.2	83.8 ± 21.4
Dopamine<br>(Arrhythmia, circulatory shock)	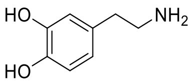	>100	-
Disulfiram<br>(Chronic alcoholism)	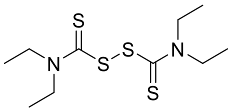	>100	-
Calcium folinate<br>(Anaemia w/ Fe^{3+}, Methotrexate toxicity)	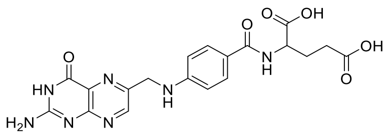	>100	--

*n = 9, ▼n = 6.

Table S2 | Half maximal inhibitory concentration (IC_{50}) values measured for selected phenothiazine-containing bioactive molecules / drugs.

**Compound** (Therapeutic Indication[28])	**Structure**	**IC_{50}, Tet(X4) (Polarization) / µM (n = 3)**	**IC_{50}, Tet(X4) (UPLC) / µM (n = 3)**
Tigecycline	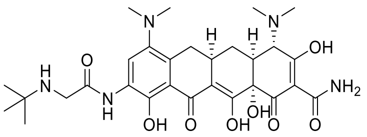	0.019 ± 0.007	-
Anhydrotetracycline	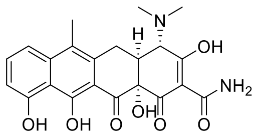	-	3.8 ± 1.1
Promazine■ (Schizophrenia, Psychosis, Psychomotor agitation)	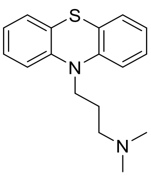	>100	>100
Trifluoperazine■ (Schizophrenia, Psychosis, Psychomotor agitation)	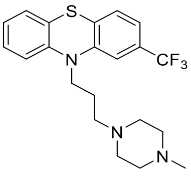	55.6 ± 14.2	83.8 ± 21.4
Phenothiazine (N/A)	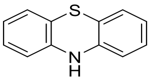	>100	128 ± 13
Levomepromazine (Schizophrenia, Amnesia, Psychomotor agitation)	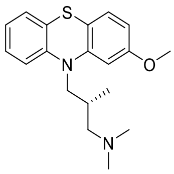	>100	>100
Oxomemazine (Antihistamine, Cough/Cold)	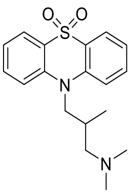	>100	>100
Triflupromazine (Psychosis, Nausea)	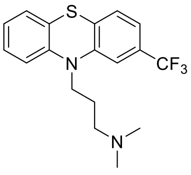	>100	102 ± 29

Chlorpromazine<br>(Schizophrenia, Mania, Nausea)	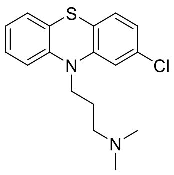	>100	110 ± 19
Prochlorperazine<br>(Schizophrenia, Nausea, Migraines)	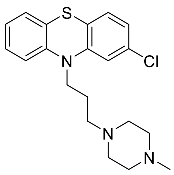	39.9 ± 7.0	49.5 ± 24.9
Acetophenazine<br>(Schizophrenia)	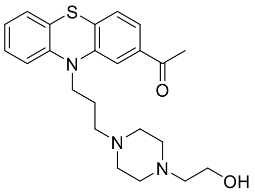	>100	>100

■ Measured previously, see Supplementary Table 1.

Table S3 | Crystallographic data collection and refinement statistics for trifluoperazine-, prochlorperazine- and tegaserod-Tet(X4) complex structures.

Parameter[a]	Ligand complexed with Tet(X4)		
	**Trifluoperazine**	**Prochlorperazine**	**Tegaserod**
**Data set**			
Wavelength / Å	0.97626	0.97626	0.97626
Resolution range / Å	48.82 – 1.90	84.52 – 2.20	59.42 – 1.90
Unique reflections	38081 (2390)	24742 (2107)	37807 (2389)
Completeness / %	100 (100)	99.8 (99.7)	100 (99.9)
*R*merge	0.130 (1.307)	0.293 (1.302)	0.154 (1.523)
*R*pim	0.036 (0.357)	0.088 (0.414)	0.034 (0.341)
CC(1/2)	0.999 (0.843)	0.991 (0.776)	0.999 (0.852)
Multiplicity	26.4 (27.3)	22.0 (20.2)	39.2 (39.5)
I/σI	19.1 (3.0)	8.1 (2.5)	16.9 (2.2)
Space group	$P6_522$	$P6_522$	$P6_522$
Unit cell parameters	a = 97.66 Å,<br>b = 97.66 Å,<br>c = 168.02 Å	a = 97.59 Å,<br>b = 97.59 Å,<br>c = 168.28 Å	a = 97.29 Å,<br>b = 97.29 Å,<br>c = 167.67 Å
**Model refinement**			
Resolution range /Å	46.70 – 1.90	75.53 – 2.20	59.43 – 1.90
No. of reflections (working/free)	37996 (1911)	24692 (1270)	37715 (1900)
No. of residues	A, 12 – 382	A, 12 - 382	A, 13 - 382
No. of water, ligand molecules	436	312	373
*R*work/*R*free / %	0.185 / 0.232	0.174 / 0.222	0.179 / 0.209
B average	26.61 Å	27.90 Å	30.16 Å
Geometry bonds / angles	0.008 Å,<br>0.99 °	0.007 Å,<br>0.88 °	0.007 Å,<br>0.945 °
Ramachandran	96.4 % / 0.0 %	96.5 % / 0.0 %	97.5 % / 0.0 %
PDB ID	**9HKE**.pdb	**9HJV**.pdb	**9HJW**.pdb

[a]Statistics of the highest resolution shell are provided in brackets where applicable.

Table S4 | Minimum inhibitory concentration (MIC) values for *E. coli* TOP10 cells containing a pBAD-TOPO-Tet(X4) plasmid in the presence of increasing amounts of the inducer, L-arabinose. See methods for experimental details.

Strain	L-arabinose concentration / $\%_{w/v}$	Tigecycline MIC / µg mL^{-1}
E. coli TOP10 + pBAD-TOPO-Tet(X4)	0	0.25
	0.0002	0.25
	0.002	0.5
	0.02	2
	0.2	8
	0.4	8

Table S5 | Broth microdilution checkerboard assay results for selected Pharmacopeia active compounds and tigecycline against *E. coli* TOP10 cells containing a pBAD-TOPO-Tet(X4) plasmid without L-arabinose induction. FICI indices of compounds tested were higher than when 0.2%$_{w/v}$ L-arabinose was added to induce Tet(X) expression, indicating that Tet(X) inhibition could potentially be a mechanism for changes in tigecycline MICs observed in combination with the inhibitors. See methods section for experimental details.

**Antibiotic MIC / µg mL^{-1}**		**Tet(X) Inhibitor MIC / µg mL^{-1}**		**Combination[a] (Antibiotic/Inhibitor) MIC / µg mL^{-1}**	**FICI[b]**	**Outcome[c]**
Tigecycline	0.25	Anhydrotetracycline	8	0.25/8	2	indifferent
		Prochlorperazine	64	0.13/32	1	indifferent
		Raloxifene	>128	0.25/>128	2	indifferent
		Tegaserod	16	0.25/16	2	indifferent
		Trifluoperazine	64	0.13/32	1	indifferent
		Tafenoquine	16	0.25/16	2	indifferent

[a]Combination MICs reported are the combinations which give the lowest FICI value. [b]FICI was calculated as $FIC_{antibiotic}$ + $FIC_{inhibitor}$, where each FIC = $MIC_{combination}$ / MIC_{alone}. [c]Outcomes are defined by the FICI value as follows: synergy (< 0.5), indifferent (0.5-4.0) or antagonistic (≥ 4.0).[29]

Table S6 | Broth microdilution checkerboard assay results for selected Pharmacopeia active compounds and tigecycline against sampled isolates possessing *tet(X)* resistance genes. See methods section for experimental details.

**Antibiotic**	**MIC / µg mL^{-1}**	**Tet(X) Inhibitor**	**MIC / µg mL^{-1}**	**Combination (Antibiotic/Inhibitor) MICs[a] / µg mL^{-1}**	**FICI[b]**	**Outcome[c]**
Salmonella enterica **T-2a –** ***tet(X4)*****[d]**						
Tigecycline	32	Anhydrotetracycline	32	8/16	0.8	indifferent
		Drosperinone	>128	16/128	1.5	indifferent
		Prochlorperazine	>128	16/64	1	indifferent
		Raloxifene	>128	16/128	1.5	indifferent
		Tegaserod	32	8/8	0.5	indifferent
		Trifluoperazine	>128	16/64	1	indifferent
Escherichia coli **T-24a –** ***tet(X4)*****[d]**						
Tigecycline	16	Anhydrotetracycline	64	4/32	0.8	indifferent
		Drosperinone	256	8/128	1	indifferent
		Prochlorperazine	256	8/32	0.6	indifferent
		Raloxifene	256	8/128	1	indifferent
		Tegaserod	16	4/8	0.8	indifferent
		Trifluoperazine	256	8/32	0.6	indifferent
Acinetobacter spp. **NT3-1 –** ***tet(X3)*** **[e]**						
Tigecycline	4	Anhydrotetracycline	2	0.5/0.25	0.3	synergy
		Drosperinone	128	4/128	2	indifferent
		Prochlorperazine	8	4/8	2	indifferent
		Raloxifene	4	2/2	1	indifferent
		Tegaserod	4	0.5/2	0.6	indifferent
		Trifluoperazine	16	4/16	2	indifferent
Acinetobacter baumannii **NT5-1 –** ***tet(X5)*** **[e]**						
Tigecycline	8	Anhydrotetracycline	16	2/4	0.5	indifferent
		Drosperinone	>128	4/128	1.5	indifferent
		Prochlorperazine	128	1/64	0.6	indifferent
		Raloxifene	>128	2/128	1.3	indifferent
		Tegaserod	32	1/16	0.6	indifferent
		Trifluoperazine	128	1/64	0.6	indifferent
Proteus penneri **NT6-2 –** ***tet(X6)*** **[e]**						
Tigecycline	8	Anhydrotetracycline	4	4/1	0.8	indifferent
		Drosperinone	>128	8/128	2	indifferent
		Prochlorperazine	>128	4/128	2	indifferent
		Raloxifene	>128	8/>128	2	indifferent
		Tegaserod	64	4/16	0.8	indifferent
		Trifluoperazine	>128	4/128	1.5	indifferent

[a]Combination MICs reported are those which gave the lowest FICI value.
[b]FICI was calculated as $FIC_{antibiotic} + FIC_{inhibitor}$, where each $FIC = MIC_{combination} / MIC_{alone}$.
[c]Outcomes are defined by the FICI value as follows: synergy (< 0.5), indifferent (0.5-4.0) or antagonistic (≥ 4.0).[29]
[d]Strains collected in Thailand as part of the CUT-SEC project (UKRI, BB/R012776/1) between 2018-2021.
[e]Strains provided by Prof. Yang Wang (China Agricultural 630 University).

Table S7 | Broth microdilution checkerboard assay results for tegaserod in combination with tetracycline and doxycycline against sampled isolates possessing *tet(X)* resistance genes. See methods section for experimental details.

**Antibiotic MIC / µg mL^{-1}**		**Tet(X) Inhibitor MIC / µg mL^{-1}**		**Combination (Antibiotic/Inhibitor) MICs[a] / µg mL^{-1}**	**FICI[b]**	**Outcome[c]**
Escherichia coli **TOP10 pBAD-TOPO-Tet(X4) – no L-arabinose**						
Tetracycline	4	Tegaserod	16	16/4	2.0	indifferent
Doxycycline	1	Tegaserod	16	8/0.5	1.0	indifferent
Escherichia coli **TOP10 pBAD-TOPO-Tet(X4) – 0.2%$_{w/v}$ L-arabinose**						
Tetracycline	128	Tegaserod	16	64/8	1.0	indifferent
Doxycycline	32	Tegaserod	16	16/4	0.8	indifferent
Salmonella enterica **T-2a –** ***tet(X4)*****[d]**						
Tetracycline	128	Tegaserod	32	32/16	0.8	indifferent
Doxycycline	32	Tegaserod	32	8/16	0.8	indifferent
Escherichia coli **T-24a –** ***tet(X4)*****[d]**						
Tetracycline	128	Tegaserod	32	32/16	0.8	indifferent
Doxycycline	32	Tegaserod	32	4/16	0.6	indifferent
Acinetobacter spp. **NT3-1 –** ***tet(X3)*** **[e]**						
Tetracycline	64	Tegaserod	8	16/4	0.8	indifferent
Doxycycline	8	Tegaserod	8	4/4	1.0	indifferent
Acinetobacter baumannii **NT5-1 –** ***tet(X5)*****[e]**						
Tetracycline	128	Tegaserod	32	128/8	1.3	indifferent
Doxycycline	32	Tegaserod	32	16/4	0.6	indifferent
Proteus penneri **NT6-2 –** ***tet(X6)*****[e]**						
Tetracycline	32	Tegaserod	64	16/32	1.0	indifferent
Doxycycline	16	Tegaserod	64	8/32	1.0	indifferent

[a]Combination MICs reported are those which gave the lowest FICI value.
[b]FICI was calculated as $FIC_{antibiotic} + FIC_{inhibitor}$, where each $FIC = MIC_{combination} / MIC_{alone}$.
[c]Outcomes are defined by the FICI value as follows: synergy (< 0.5), indifferent (0.5-4.0) or antagonistic (≥ 4.0).[29]
[d]Strains collected in Thailand as part of the CUT-SEC project (UKRI, BB/R012776/1) between 2018-2021.
[e]Strains provided by Prof. Yang Wang, (China Agricultural 630 University).

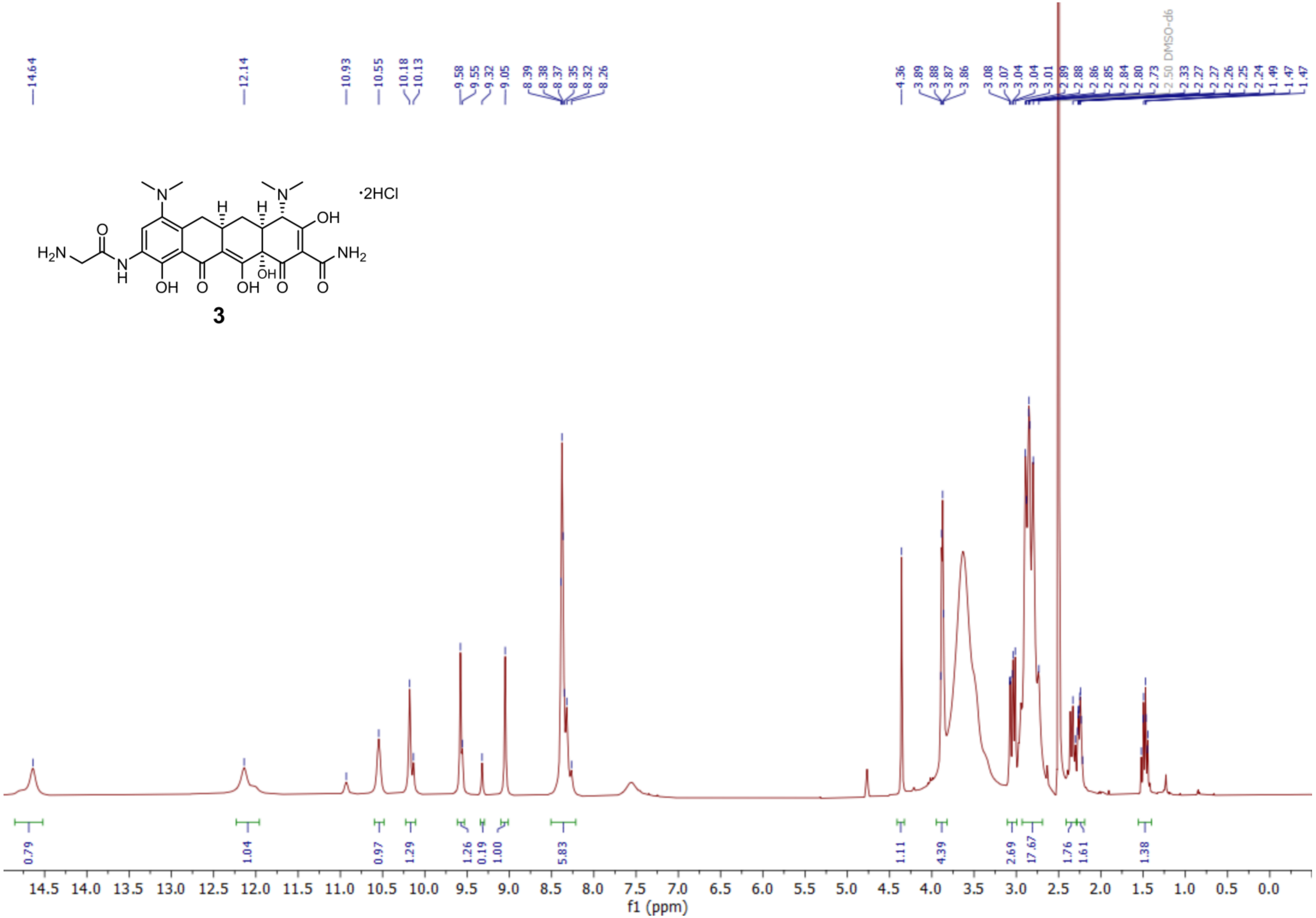

^{1}H NMR (500 MHz, DMSO-d_6) spectrum of 9-glycylamido-minocycline 3.

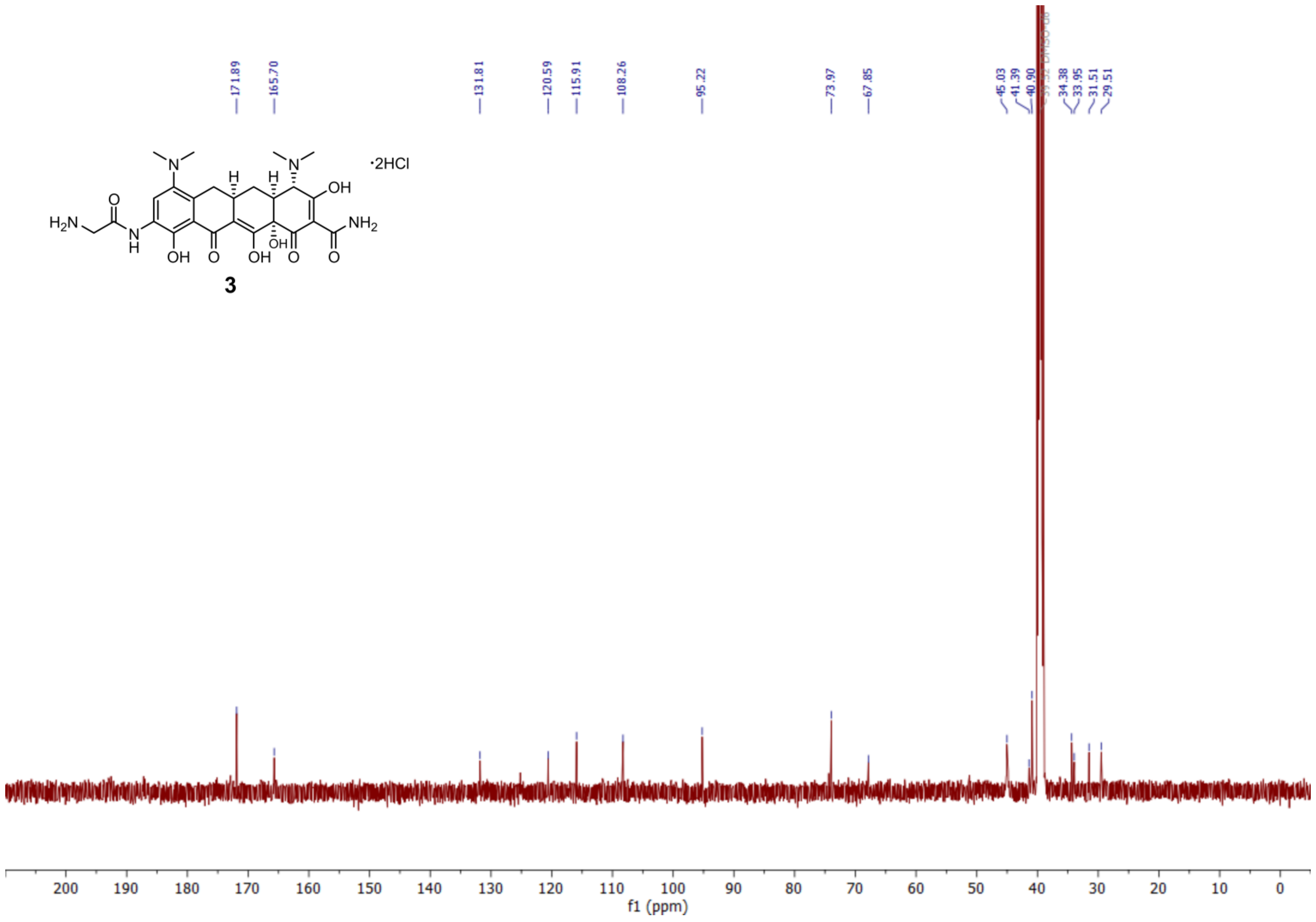

^{13}C NMR (126 MHz, DMSO-*d*$_6$) spectrum of 9-glycylamido-minocycline 3.

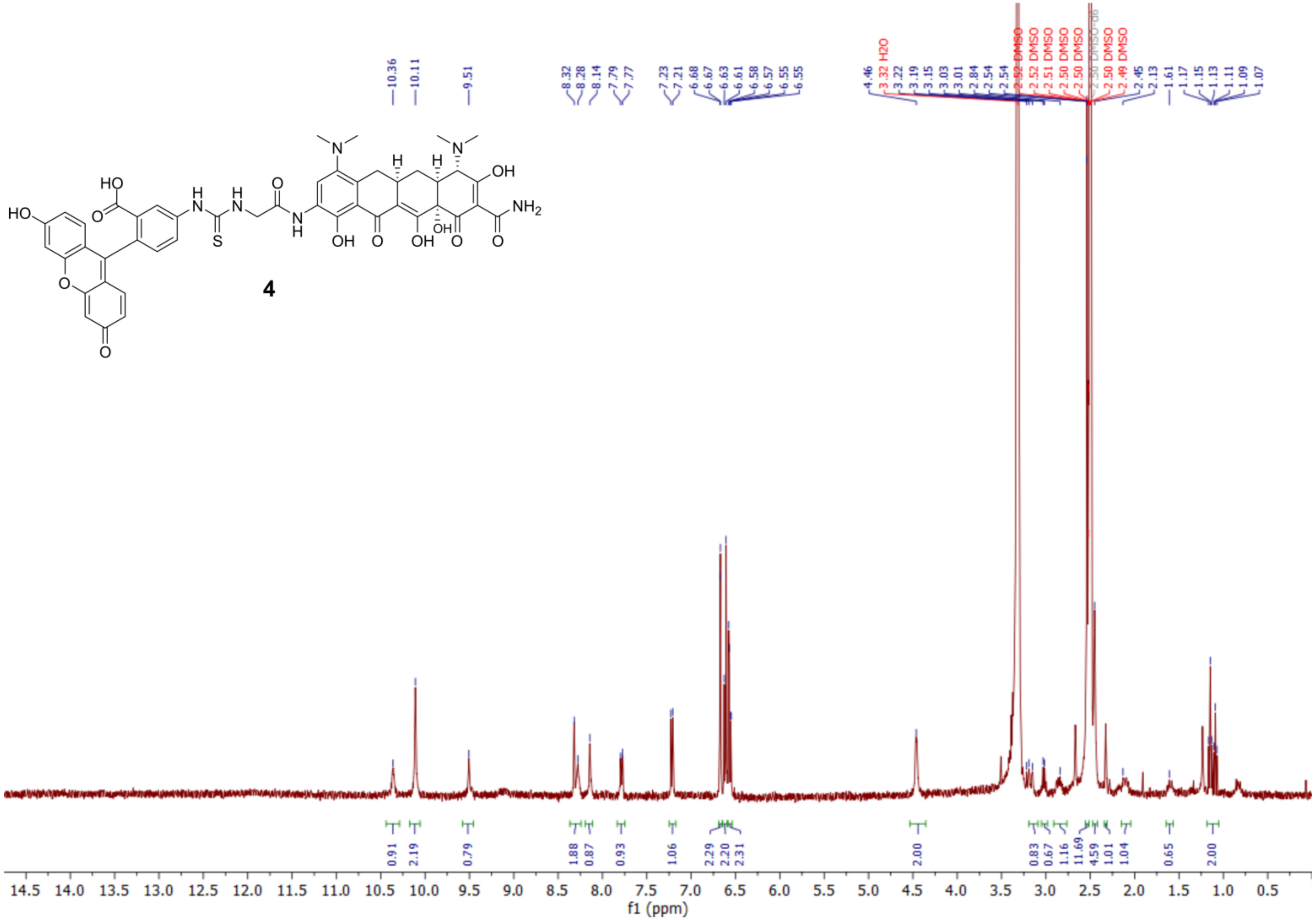

^{1}H NMR (400 MHz, DMSO-d_6) of the FITC-glycylamido-minocycline probe 4.

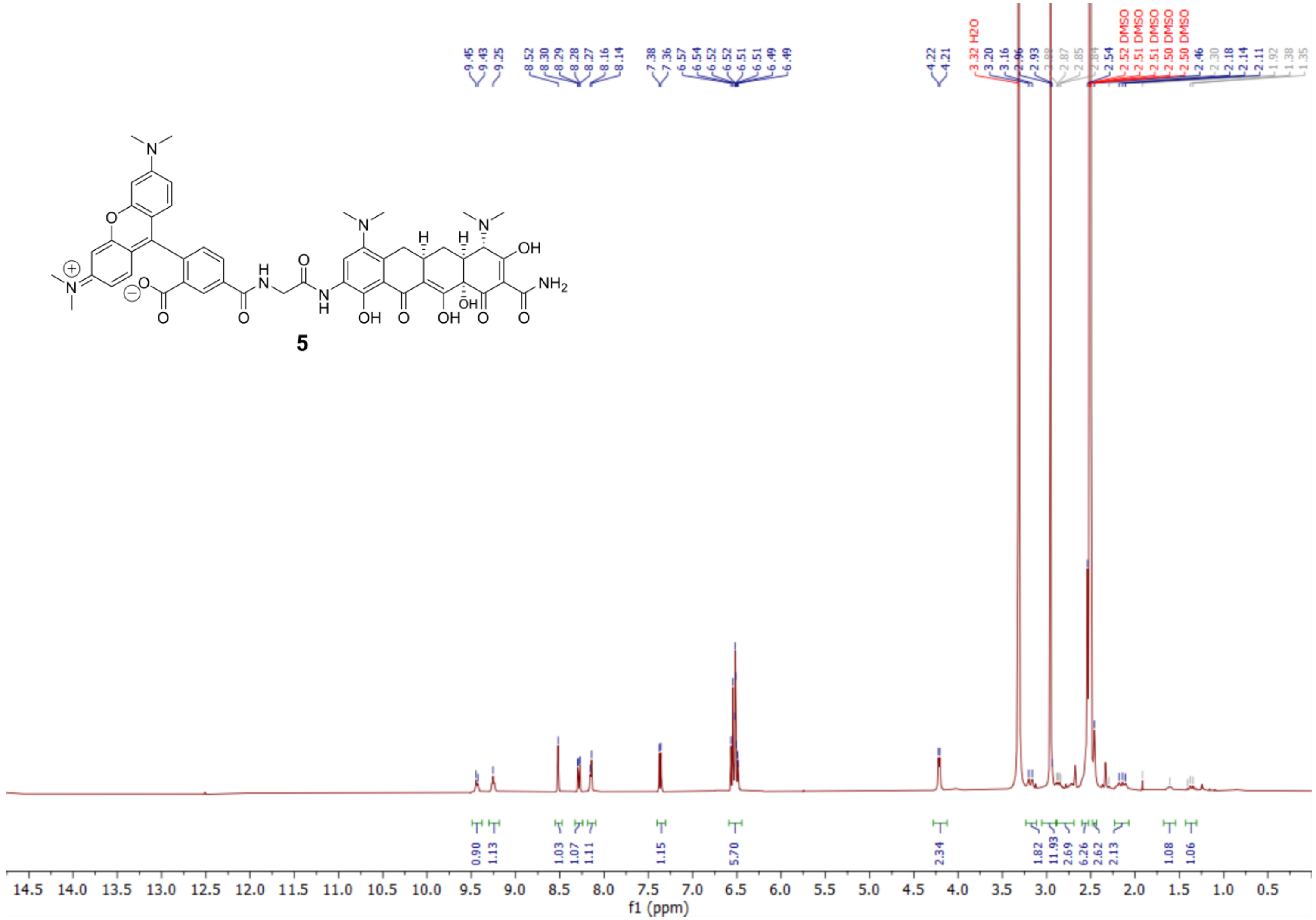

^{1}H NMR (400 MHz, DMSO-*d*$_6$) spectrum of the 5-TAMRA-glycylamido-minocycline probe 5.

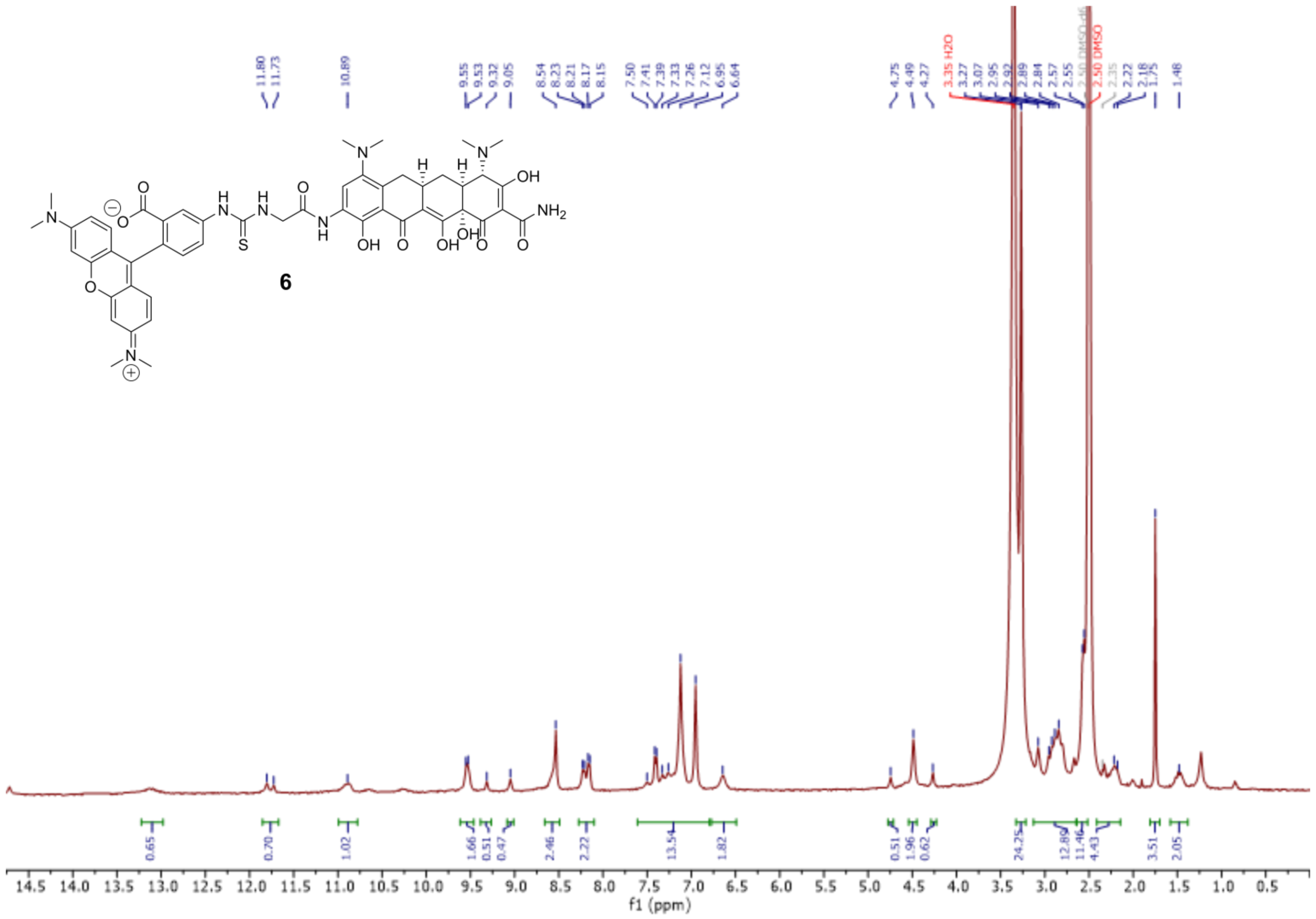

^{1}H NMR (400 MHz, DMSO-*d_6*) spectrum of the 5-TRITC-glycylamidominocycline probe 6.

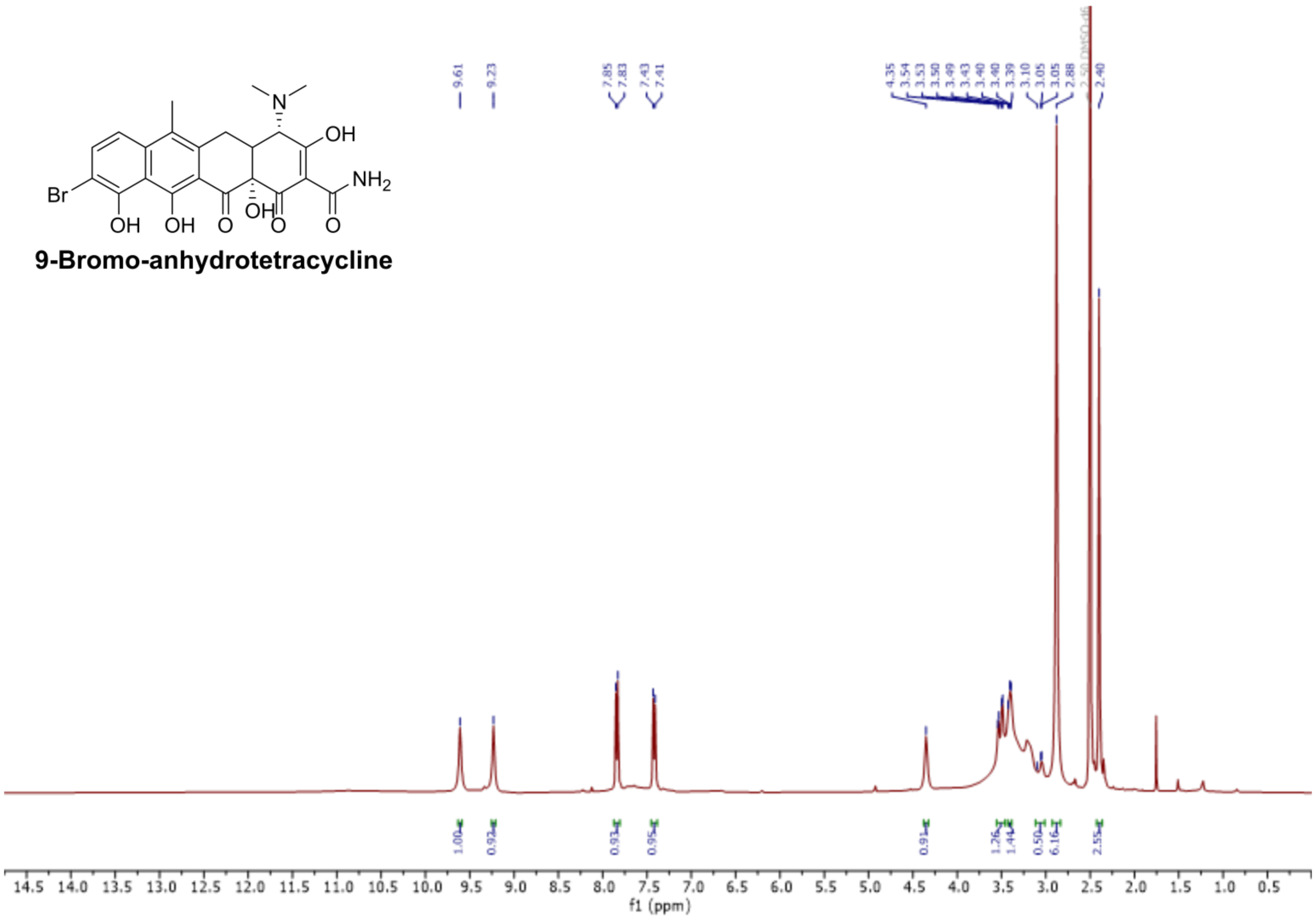

^{1}H NMR (400 MHz, DMSO-d_6) spectrum of 9-bromo-anhydrotetracycline.

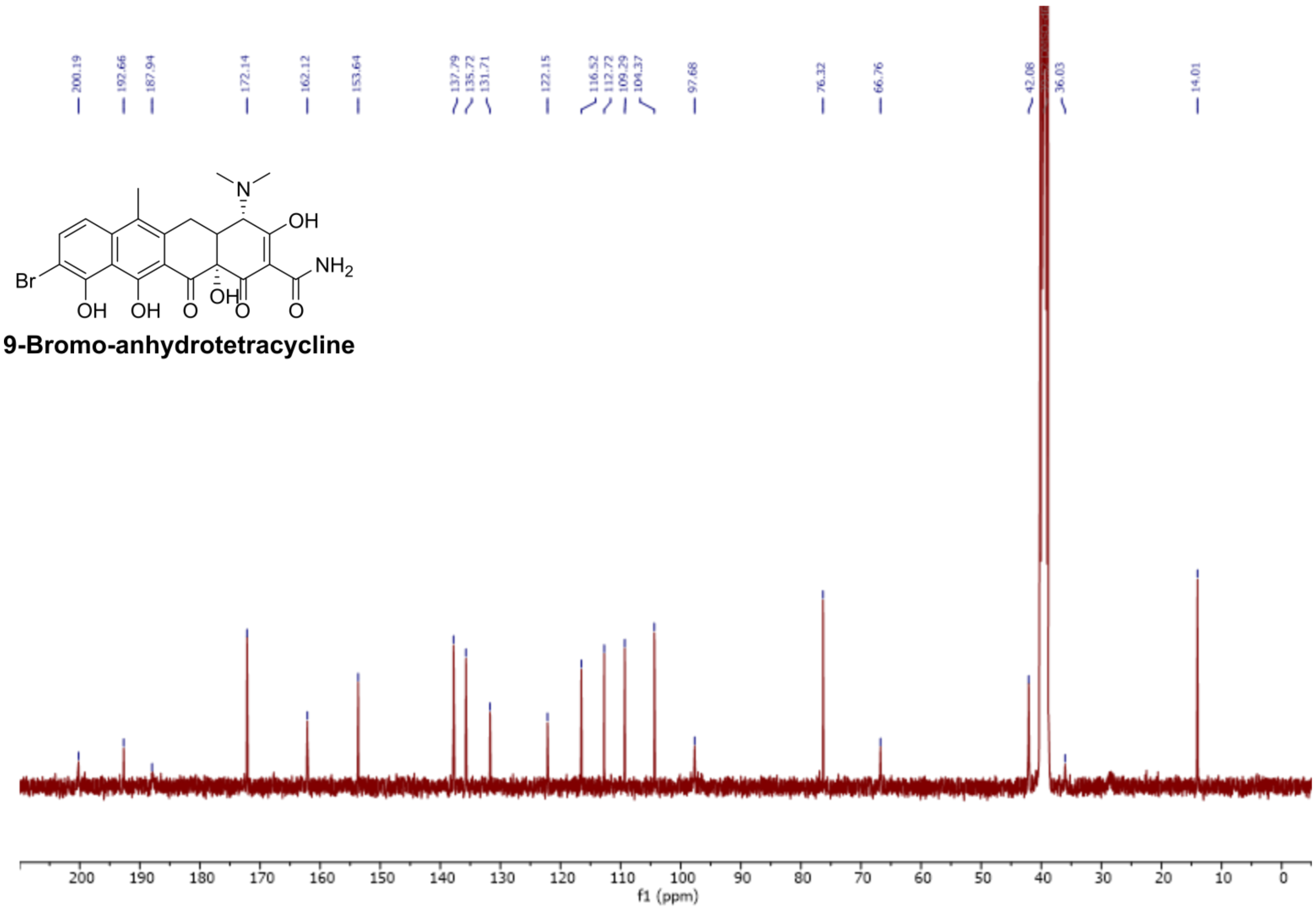

^{13}C NMR (101 MHz, DMSO-*d*$_6$) spectrum of 9-bromo-anhydrotetracycline.

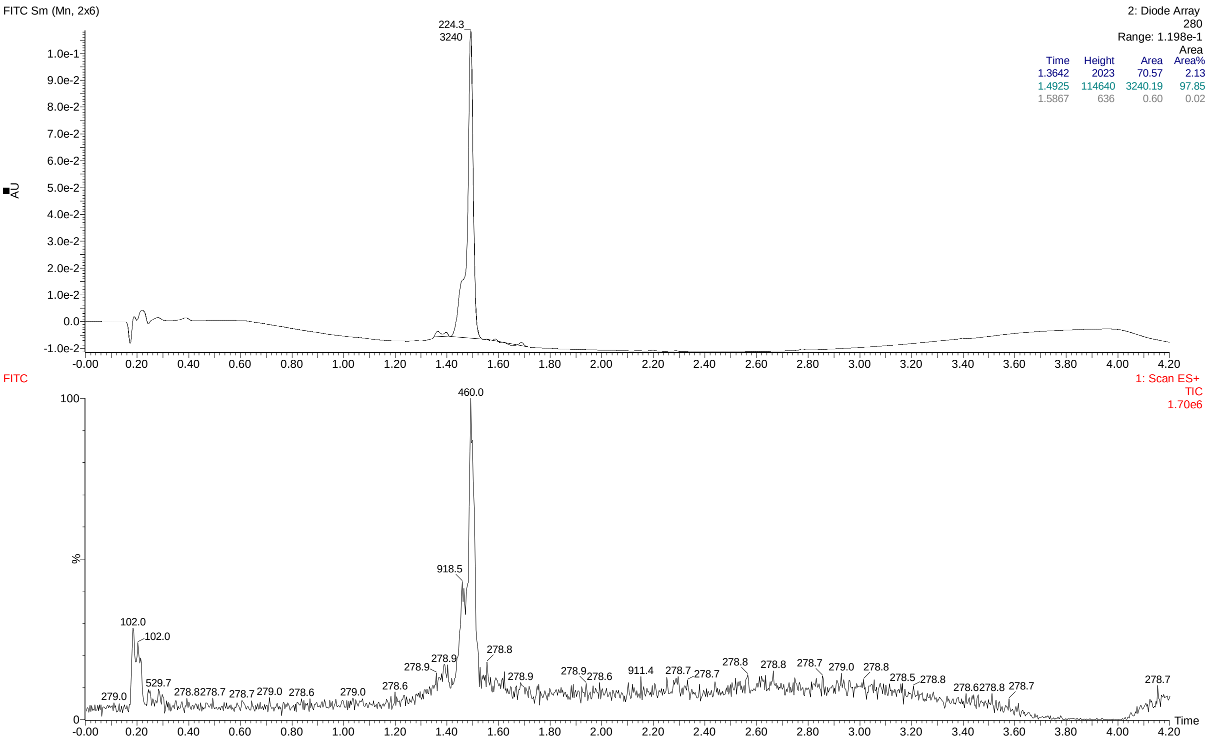

UPLC-MS chromatograms investigating purity of the FITC-glycylamido-minocycline probe 4. Chromatograms show the absorbance at 280 nm (*top*) and total ion chromatogram (*bottom*).

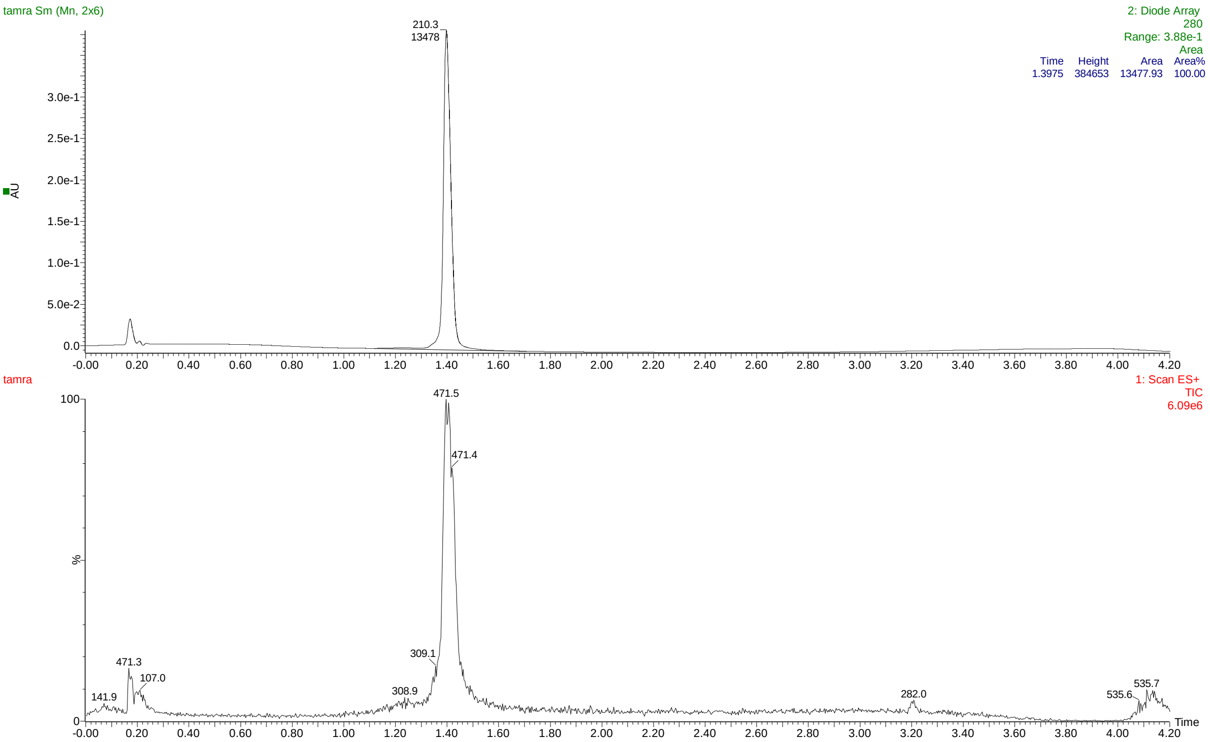

UPLC-MS chromatograms investigating purity of the TAMRA-glycylamido-minocycline probe 5. Chromatograms show the absorbance at 280 nm (*top*) and total ion chromatogram (*bottom*).

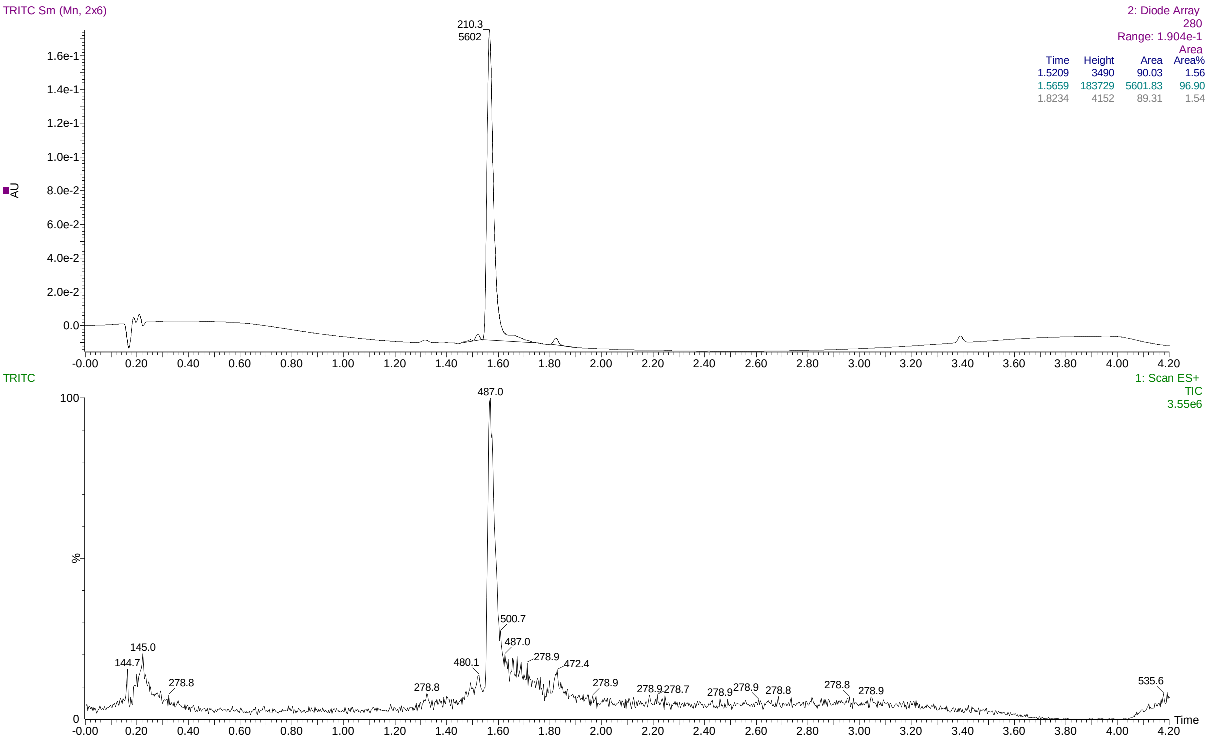

UPLC-MS chromatograms investigating purity of the TRITC-glycylamido-minocycline probe 6. Chromatograms show the absorbance at 280 nm (*top*) and total ion chromatogram (*bottom*).

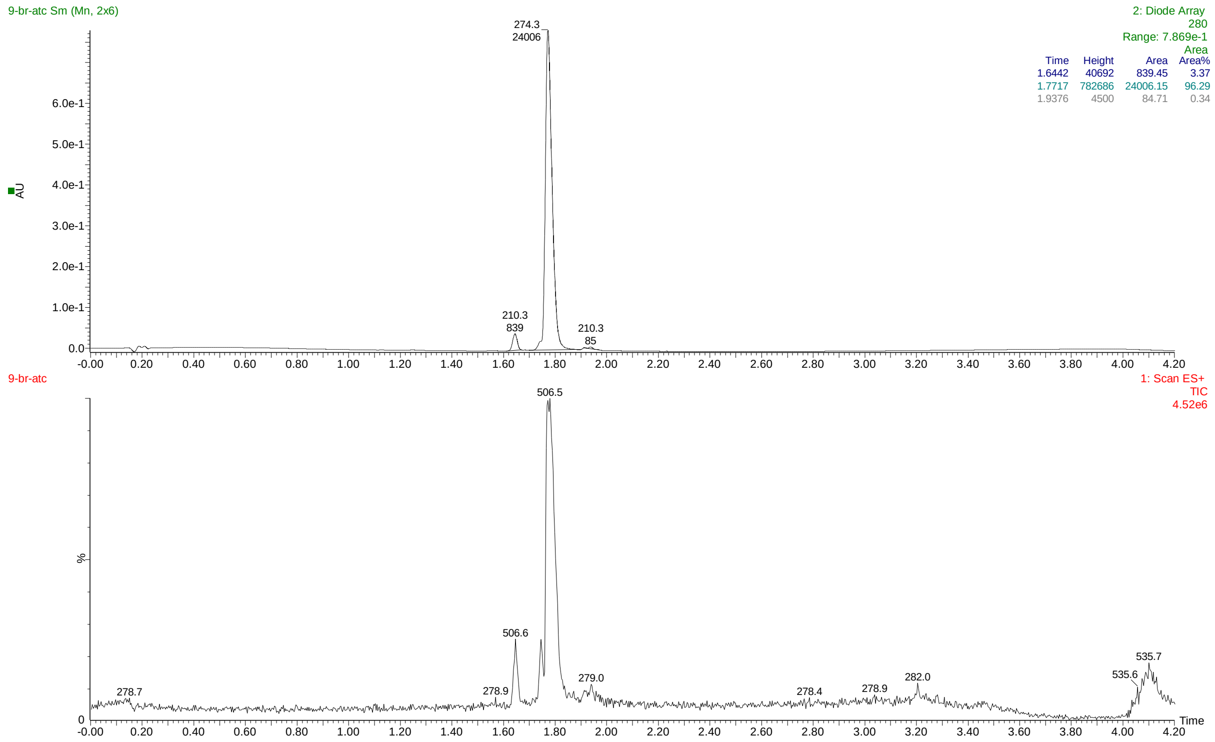

UPLC-MS chromatograms investigating purity of 9-bromo-anhydrotetracycline. Chromatograms show the absorbance at 280 nm (*top*) and total ion chromatogram (*bottom*).

References

1 C. Chen, A. R. Zeiger and E. Wickstrom, *Bioorg. Med. Chem. Lett.*, 2007, **17**, 6558–6562.
2 J. L. Markley, L. Fang, A. J. Gasparrini, C. T. Symister, H. Kumar, N. H. Tolia, G. Dantas and T. A. Wencewicz, *ACS Infect. Dis.*, 2019, **5**, 618–633.
3 D. Cappel, S. Jerome, G. Hessler and H. Matter, *J. Chem. Inf. Model.*, 2020, **60**, 1432–1444.
4 O. Trott and A. J. Olson, *J. Comput. Chem.*, 2010, **31**, 455–461.
5 Q. Cheng, Y. Cheung, C. Liu, Q. Xiao, B. Sun, J. Zhou, E. W. C. Chan, R. Zhang and S. Chen, *BMC Biol.*, 2021, **19**, 262.
6 N. Eswar, B. Webb, M. A. Marti-Renom, M. s. Madhusudhan, D. Eramian, M. Shen, U. Pieper and A. Sali, *Curr. Protoc. Bioinformatics*, 2006, **15**, 5.6.1-5.6.30.
7 E. F. Pettersen, T. D. Goddard, C. C. Huang, G. S. Couch, D. M. Greenblatt, E. C. Meng and T. E. Ferrin, *J. Comput. Chem.*, 2004, **25**, 1605–1612.
8 C. J. Williams, J. J. Headd, N. W. Moriarty, M. G. Prisant, L. L. Videau, L. N. Deis, V. Verma, D. A. Keedy, B. J. Hintze, V. B. Chen, S. Jain, S. M. Lewis, W. B. Arendall, J. Snoeyink, P. D. Adams, S. C. Lovell, J. S. Richardson and D. C. Richardson, *Protein Sci.*, 2018, **27**, 293–315.
9 J. C. Gordon, J. B. Myers, T. Folta, V. Shoja, L. S. Heath and A. Onufriev, *Nucleic Acids Res.*, 2005, **33**, W368–W371.
10 RDKit: Open-source cheminformatics http://www.rdkit.org.
11 P. J. Ropp, J. C. Kaminsky, S. Yablonski and J. D. Durrant, *J. Cheminform.*, 2019, **11**, 14.
12 M. J. Frisch, G. W. Trucks, H. B. Schlegel, G. E. Scuseria, M. A. Robb, J. R. Cheeseman, G. Scalmani, V. Barone, G. A. Petersson, H. Nakatsuji, X. Li, M. Caricato, A. V. Marenich, J. Bloino, B. G. Janesko, R. Gomperts, B. Mennucci, H. P. Hratchian, J. V. Ortiz, A. F. Izmaylov, J. L. Sonnenberg, Williams, F. Ding, F. Lipparini, F. Egidi, J. Goings, B. Peng, A. Petrone, T. Henderson, D. Ranasinghe, V. G. Zakrzewski, J. Gao, N. Rega, G. Zheng, W. Liang, M. Hada, M. Ehara, K. Toyota, R. Fukuda, J. Hasegawa, M. Ishida, T. Nakajima, Y. Honda, O. Kitao, H. Nakai, T. Vreven, K. Throssell, J. A. Montgomery Jr., J. E. Peralta, F. Ogliaro, M. J. Bearpark, J. J. Heyd, E. N. Brothers, K. N. Kudin, V. N. Staroverov, T. A. Keith, R. Kobayashi, J. Normand, K. Raghavachari, A. P. Rendell, J. C. Burant, S. S. Iyengar, J. Tomasi, M. Cossi, J. M. Millam, M. Klene, C. Adamo, R. Cammi, J. W. Ochterski, R. L. Martin, K. Morokuma, O. Farkas, J. B. Foresman and D. J. Fox, Gaussian 16 Rev. C.01 2016.
13 G. M. Morris, R. Huey, W. Lindstrom, M. F. Sanner, R. K. Belew, D. S. Goodsell and A. J. Olson, *J. Comput. Chem.*, 2009, **30**, 2785–2791.
14 F. Madeira, N. Madhusoodanan, J. Lee, A. Eusebi, A. Niewielska, A. R. N. Tivey, R. Lopez and S. Butcher, *Nucleic Acids Res.*, 2024, **52**, W521–W525.
15 R. C. Edgar, *Nucleic Acids Res.*, 2004, **32**, 1792–1797.
16 A. M. Waterhouse, J. B. Procter, D. M. A. Martin, M. Clamp and G. J. Barton, *Bioinformatics*, 2009, **25**, 1189–1191.
17 G. Volkers, J. M. Damas, G. J. Palm, S. Panjikar, C. M. Soares and W. Hinrichs, *Acta Crystallogr. D Biol. Crystallogr.*, 2013, **69**, 1758–1767.
18 J. Park, A. J. Gasparrini, M. R. Reck, C. T. Symister, J. L. Elliott, J. P. Vogel, T. A. Wencewicz, G. Dantas and N. H. Tolia, *Nat. Chem. Biol.*, 2017, **13**, 730–736.
19 M. L. Connolly, *J. Appl. Crystallogr.*, 1983, **16**, 548–558.
20 W. Yang, I. F. Moore, K. P. Koteva, D. C. Bareich, D. W. Hughes and G. D. Wright, *J. Biol. Chem.*, 2004, **279**, 52346–52352.
21 I. F. Moore, D. W. Hughes and G. D. Wright, *Biochemistry*, 2005, **44**, 11829–11835.
22 L. Xu, Y. Zhou, S. Niu, Z. Liu, Y. Zou, Y. Yang, H. Feng, D. Liu, X. Niu, X. Deng, Y. Wang and J. Wang, *eBioMedicine*, 2022, **78**, 103943.
23 Y. Liu, Y. Jia, K. Yang, R. Li, X. Xiao and Z. Wang, *Commun. Biol.*, 2020, **3**, 1–10.
24 L. Xu, Y. Zhou, D. Ou, H. Yang, H. Feng, H. Song, N. Xie, X. Niu, X. Deng, M. Sun, P. Zhang, D. Liu and J. Wang, *One Health Adv.*, 2024, **2**, 1.
25 J. L. Markley and T. A. Wencewicz, *Front. Microbiol.*, 2018, **9**, 1058.
26 D. Liebschner, P. V. Afonine, N. W. Moriarty, B. K. Poon, O. V. Sobolev, T. C. Terwilliger and P. D. Adams, *Acta Crystallogr. D Biol. Crystallogr.*, 2017, **73**, 148–157.

27 D. Liebschner, P. V. Afonine, M. L. Baker, G. Bunkóczi, V. B. Chen, T. I. Croll, B. Hintze, L.-W. Hung, S. Jain, A. J. McCoy, N. W. Moriarty, R. D. Oeffner, B. K. Poon, M. G. Prisant, R. J. Read, J. S. Richardson, D. C. Richardson, M. D. Sammito, O. V. Sobolev, D. H. Stockwell, T. C. Terwilliger, A. G. Urzhumtsev, L. L. Videau, C. J. Williams and P. D. Adams, *Acta Crystallogr. D Biol. Crystallogr.*, 2019, **75**, 861–877.
28 C. Knox, M. Wilson, C. M. Klinger, M. Franklin, E. Oler, A. Wilson, A. Pon, J. Cox, N. E. (Lucy) Chin, S. A. Strawbridge, M. Garcia-Patino, R. Kruger, A. Sivakumaran, S. Sanford, R. Doshi, N. Khetarpal, O. Fatokun, D. Doucet, A. Zubkowski, D. Y. Rayat, H. Jackson, K. Harford, A. Anjum, M. Zakir, F. Wang, S. Tian, B. Lee, J. Liigand, H. Peters, R. Q. (Rachel) Wang, T. Nguyen, D. So, M. Sharp, R. da Silva, C. Gabriel, J. Scantlebury, M. Jasinski, D. Ackerman, T. Jewison, T. Sajed, V. Gautam and D. S. Wishart, *Nucleic Acids Res.*, 2024, **52**, D1265–D1275.
29 F. C. Odds, *Journal of Antimicrobial Chemotherapy*, 2003, **52**, 1.
